# Observations on an Open‐Label Phase 1/2 Dopamine Gene Therapy Trial (OXB‐102/Axo‐Lenti‐PD) in People with Parkinson's Disease

**DOI:** 10.1002/mds.70378

**Published:** 2026-05-28

**Authors:** Simon Rowe, Amy Evans, Saeed Kayhanian, Philip C. Buttery, Robert Morris, Katie Andresen, Laura Sherlock, Samuel Hewitt, Tagore Nakornchai, Marwan Hariz, Kyriacos Mitrophanous, Ludvic Zrinzo, Matthew Appleby, Phil Woodgate, Seema Maru, Patricia Limousin, Christine Girges, Thomas Foltynie, Stephane Palfi, Roger A. Barker

**Affiliations:** ^1^ Faculty of Medicine and Health University of New South Wales Sydney Australia; ^2^ Department of Clinical Neurosciences and Cambridge Stem Cell Institute University of Cambridge Cambridge United Kingdom; ^3^ Department of Neurosurgery Cambridge University Hospitals Cambridge United Kingdom; ^4^ UCL Institute of Neurology and The National Hospital for Neurology and Neurosurgery London United Kingdom; ^5^ Deparment of Clinical Neuroscience Umeå University Umeå Sweden; ^6^ Oxford Biomedica (UK) Limited Oxford United Kingdom; ^7^ Department of Neurosurgery, Henri Mondor Academic Hospital Université Paris Est Créteil, GRCT OPTIMA, INSERM Laboratory U955 of Translational NeuroPsychiatry, Institut Mondor de Recherche Biomédicale Créteil France

**Keywords:** clinical trial, gene therapy, Parkinson's disease

## Abstract

**Background:**

SUNRISE‐PD was a dose‐escalating, phase 1/2 study investigating a second‐generation lentiviral vector gene therapy delivering the genes for dopamine synthesis (OXB‐102) to treat Parkinson's disease (PD). The trial was prematurely terminated due to insolvency of the sponsor.

**Objectives:**

The aim was to provide an investigator‐led description of the clinical course for the 6 patients enrolled in the OXB‐102 trial.

**Methods:**

Individual patient data were extracted, compiled, and summarized from investigator records.

**Results:**

Six patients received a low (n = 2) and a higher (n = 4) dose of the OXB‐102 gene therapy. There were eight serious adverse events (SAE), of which only one was considered definitely related to the intervention. All SAEs were transient and resolved without sequelae. Efficacy data were limited, but some stabilization of motor function was observed in most of the patients.

**Conclusion:**

This novel dopamine gene therapy appears safe in patients with moderate‐to‐severe PD, with some possible stabilizing effects on their clinical course. © 2026 The Author(s). *Movement Disorders* published by Wiley Periodicals LLC on behalf of International Parkinson and Movement Disorder Society.

Parkinson's disease (PD) is a progressive neurodegenerative disorder characterized by the loss of dopaminergic neurons in the substantia nigra pars compacta and their projection to the striatum, which leads to the hallmark motor features of bradykinesia and rigidity.^1^ These features emerge when dopaminergic neuronal loss exceeds 50% and can be ameliorated in early‐stage disease using drugs that replace this lost input, such as levodopa (l‐dopa) and dopamine agonists.^2^, ^3^ As PD progresses, escalating doses of l‐dopa lead to excessive involuntary movements (dyskinesias), whereas the use of dopamine agonists is frequently limited by off‐target dopaminergic effects such as neuropsychiatric and impulse control disorders.^4^

Gene therapies designed to enhance dopaminergic signaling form one of several innovative strategies, alongside continuous dopaminergic infusion therapies and dopamine cell replacement, designed to address the limitations of oral dopaminergic therapies.^5^ Several gene therapy strategies have been investigated to date, particularly in mid‐to‐late disease stages.^6^ One such approach involves viral vector‐mediated gene transfer to enhance dopamine production within the striatum itself, bypassing the lost nigrostriatal pathway and restoring dopaminergic activity at this site.^6^

We previously reported on a first‐generation lentiviral vector, ProSavin, developed by Oxford BioMedica that was shown to be safe and well tolerated, with improvement in motor behavior observed in all patients.^7^ After this, AXO‐Lenti‐PD was developed as a more efficacious second‐generation construct, delivering key genes for dopamine synthesis. This included tyrosine hydroxylase (TH), guanosine triphosphate (GTP)‐cyclohydrolase 1 (CH1), and aromatic l‐amino acid decarboxylase in an optimized gene order, with fusion constructs linking two of the three transgenes (TH:CH1), which achieved at least a fivefold greater dopamine production per transduced cell in preclinical models.^8^

A two‐part, phase 1/2 study (OXB‐102‐01) was designed to evaluate the safety, efficacy, and optimal intrastriatal dosing of AXO‐Lenti‐PD in moderate‐stage PD. Early results of OXB‐102‐01 were announced in 2019 after preliminary analysis of the 6‐month endpoints in the first 2 enrolled participants.^9^ The trial was subsequently terminated in 2022 due to sponsor insolvency.^10^

At the time of trial termination, 6 participants had received treatment. Two received the lowest dose; 4 received the second of three planned doses. They have since been followed up as part of investigator due diligence and routine clinical care. We report their clinical course and outcomes. No formal statistical analysis was undertaken due to the small, open‐label design and premature termination of the study, recruiting only 25% of the planned sample. Despite these limitations, we believe reporting these data is important given the novel intervention and our ethical obligation to these participants who volunteered for this first‐in‐human study.

## Patients and Methods

SUNRISE‐PD (OXB‐102‐01, NCT03720418) was designed as a two‐part study of OXB‐102 (AXO‐Lenti‐PD), a lentiviral vector gene therapy product, delivered by stereotactic neurosurgical injection into the putamen of people with PD aged between 48 and 70 (Full Trial Protocol in Supplementary Appendix S1). Part A consisted of an open‐label dose‐escalation phase, intending to recruit up to 30 individuals, to be followed by part B, a randomized, double‐blind phase including a control arm with imitation surgery. The study sponsor was Sio Gene Therapies, formerly known as Axovant.

The primary outcome of this study was safety at 3 months post‐treatment, and secondary outcomes were measures of clinical efficacy, including changes in the Unified Parkinson's Disease Rating Scale (UPDRS) after 6 months. Detailed methods, including inclusion/exclusion criteria, details of the intervention, and planned assessments of safety, efficacy, and exploratory endpoints, are included in Supplementary Appendix S1.

Longitudinal follow‐up occurred every 6 months for 2 years and thereafter annually for 15 years postintervention. A full medication review was performed at each time point, and a levodopa equivalent daily dose (LEDD) was calculated.^11^ Adverse events were classified using MedDRA preferred terms and graded by Common Terminology Criteria for Adverse Events (CTCAE) criteria.

The trial was conducted between 2018 and 2022. At termination of the trial, 6 patients had been treated in part A: 4 at Addenbrooke's Hospital in Cambridge, UK, and 2 at the National Hospital for Neurology and Neurosurgery, in London, UK. Data regarding adverse events, efficacy, and medication use were collected from both study sites and collated manually, as access to the original database was lost once the company became insolvent.

## Results

Patients A to F were recruited sequentially, with A and B receiving the lowest dose (2.64 × 10^6^ TU in 600 μL total infusion volume) and C to F receiving a higher dose (9.0 × 10^6^ TU in 600 μL total infusion volume). The cohort's baseline characteristics are summarized in Table 1.

**TABLE 1:** Baseline characteristics of cohort

	Patient A	Patient B	Patient C	Patient D	Patient E	Patient F
Sex (M/F)	F	M	M	M	M	M
Age (yr)	56	50	51	60	64	52
Years since diagnosis	13	16	7	13	20	14
H&Y stage (*off*)	4	3	3	4	3	3
MDS‐UPDRS Part III (*off*)	60	58	50	55	45	53
MDS‐UPDRS total (*off*)	107	91	69	78	80	87
LEDD (mg)	1042	1125	1340	2565	1613	1384

Abbreviations: H&Y, Hoehn and Yahr; MDS‐UPDRS, Movement Disorder Society‐Unified Parkinson's Disease Rating Scale; LEDD, levodopa equivalent daily dose.

### Primary Outcome

Eight serious adverse events occurred in 3 patients. One was considered definitely related to the study intervention (surgical site infection), and three were deemed possibly related to the intervention (headache, confusion, anxiety). Four events were considered unrelated to the intervention (gastrointestinal hemorrhage, fall, delirium, psychotic depression).

One hundred and thirty mild or moderate adverse events were recorded. The most common of these were autonomic, including constipation, postural hypotension, and hyperhidrosis (n = 16); motoric, including dyskinesias or on–off motor fluctuations (n = 12); and neuropsychiatric such as worsening anxiety or suicidal ideation (n = 10).

The postoperative magnetic resonance imaging (MRI) follow‐up indicated that in all cases the delivery was correctly located within the posterior putamen.

No unexplained abnormalities on brain MRI, laboratory parameters, vital signs, or physical examination were observed.

### Secondary Outcomes

The completeness of secondary endpoint measurement and data collection was adversely affected by several factors. This included restrictions due to the COVID‐19 pandemic, affecting 3 patients for four visits. One participant declined to have *off* medication assessments after the third study visit. The study was also terminated before any patients were enrolled in the third (highest) planned dose level due to the company becoming insolvent.

At the 6‐month visit, 5 of 6 participants experienced a reduction in their LEDD of between −8% and −30%. Of the 5 participants who consented to ongoing *off* assessments, all had improvements in the MDS‐UPDRS Part III score, summarized in Table 2. There was no apparent difference in the two dosed groups.

**TABLE 2:** The changes in clinical measures in the cohort at 6 months after treatment

	Patient A	Patient B	Patient C	Patient D	Patient E	Patient F
Hoehn and Yahr stage in the OFF off state	0	0	–1	N/A	0	–1
LEDD, mg/day (%)	−132 (−13)	−150 (−13)	−100 (−8)	−700 (−27)	−488 (−30)	+75 (+5)
MDS‐UPDRS *off* Part III (%)	−20 (−33)	−14 (−24)	−22 (−44)	N/A	−10 (−22)	−19 (−36)
MDS‐UPDRS *off* total (%)	−48 (−45)	−33 (−36)	−32 (−46)	N/A	0 (0)	−35 (−40)
Rush Dyskinesia Rating Scale in *on* state	+2	−4	0	−2	+1	0
Average % of awake time in the *off* state	+15	−2	−3	+2	−20	−18
PDQ‐39 score	0	−37	+16	+3	+4	+2
EQ‐VAS	−10	+20	+21	−5	+12	+3

*Note*: N/A: measure not available (patient declined “OFF” testing).

Abbreviations: LEDD, levodopa equivalent daily dose; MDS‐UPDRS, Movement Disorder Society‐Unified Parkinson's Disease Rating Scale; PDQ‐39, Parkinson's Disease Questionnaire‐39; EQ‐VAS, EuroQol Visual Analogue Scale.

**FIG. 1:**
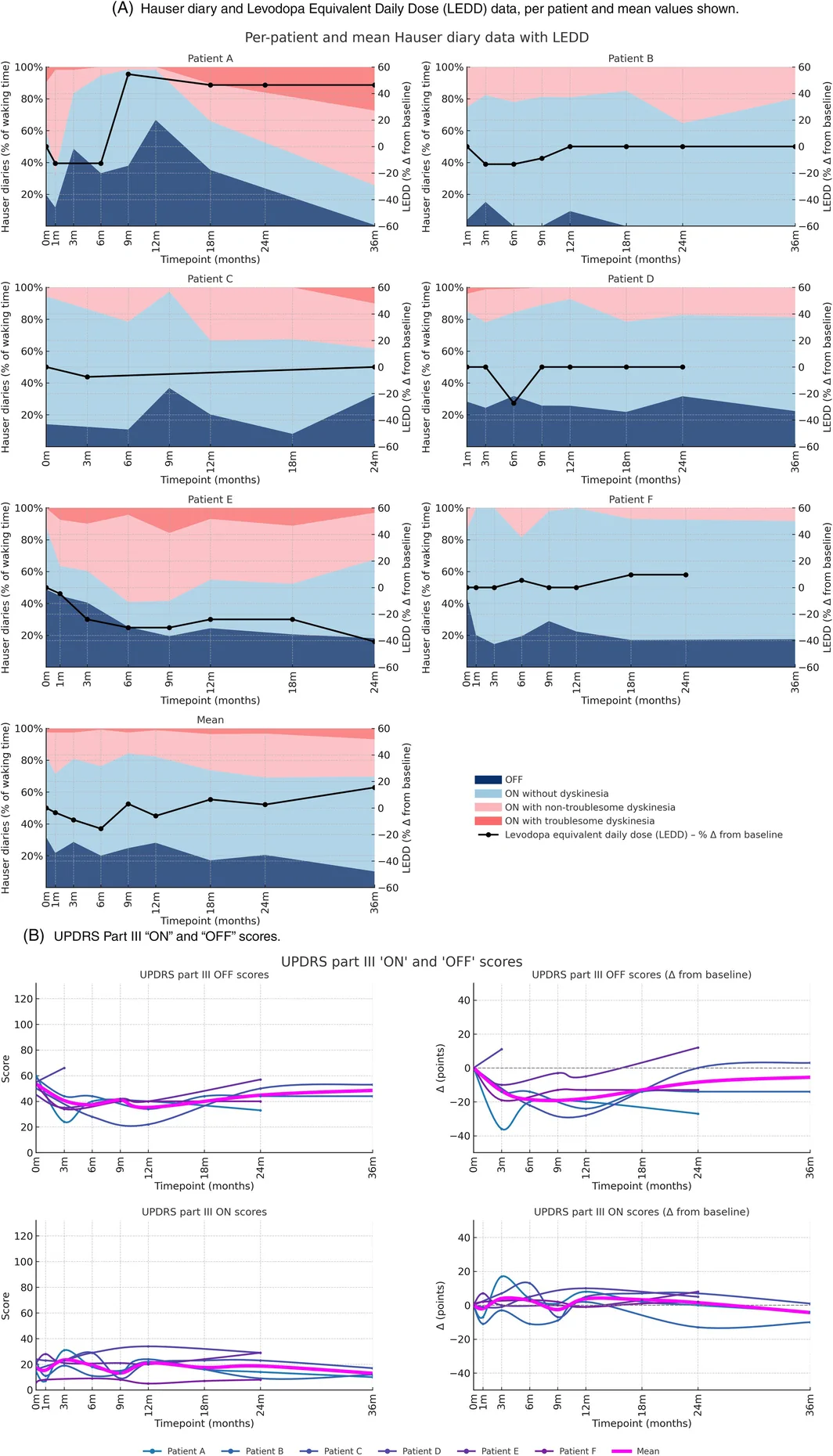
**(A)** Hauser diary and levodopa equivalent daily dose (LEDD) data, per patient, and mean values shown. **(B)** UPDRS Part III *on* and *off* scores. [Color figure can be viewed at wileyonlinelibrary.com]

At the 2‐ and 3‐year follow‐up visits, the LEDD was unchanged in 3 patients (B, C, and D), increased in 2 (A and F), and reduced due to neuropsychiatric side effects in 1 (E), summarized alongside Hauser diary reports in Figure 1A. The UPDRS Part III scores (Fig. 1B) showed persistent improvement in 3 patients (A, B, and F) and worsening in 2 (C and E) and were not assessed in 1 (D). There was again no apparent difference in effect between the differently dosed cohorts.

## Discussion

Anatomically targeted dopamine gene therapy provides a biologically attractive strategy in PD, particularly in moderate or late stages where conventional options are limited. Despite enrolling a fraction of its intended cohort, OXB‐102‐01 contributes to the growing evidence that striatal gene transfer is technically feasible, generally well tolerated, and potentially capable of offering motor benefits. However, early termination of the trial with limited follow‐up limits the conclusions that we can draw about its utility in PD.

### Safety and Tolerability

Despite premature discontinuation, the study provides reassuring safety data for putamenal dopaminergic gene therapy in moderate‐to‐severe PD. Early complications were minimal and transient, with no sustained or emergent clinically significant adverse events, with up to 3 years of follow‐up. These findings align with the emerging safety record for intracerebral gene‐ and cell‐based therapies. The durability of the safety signal over extended follow‐up provides confidence that the approach does not lead to any delayed neurotoxicity or aberrant biological activity or cause other latent complications that have historically been of concern in the field.^12^

### Signals of Efficacy

The limited data suggest a possible therapeutic signal in some participants. Reductions in mean *off* time in Hauser diary records and mean LEDD (3 of 6 patients) were observed within 12 months, alongside improvements in UPDRS Part III *off* scores in 4 of 6 patients.^13^ These findings support further evaluation, including dose finding, to determine clinical relevance.

### Limitations

This prematurely terminated study has significant limitations. Clearly, the termination prior to planned endpoints limits interpretation around efficacy data. This was further limited by the impact of the COVID‐19 pandemic on data completeness. The original dose‐finding objective was not completed, so true relationships between dose, effect, and toxicity remain incompletely defined. This limits any interpretation one could draw about the differences in dose–response, especially as biologically pertinent endpoints, such as biodistribution of the lentiviral vector in blood and urine and its target engagement using positron emission tomography imaging, were not completed. Despite these limitations, we are publishing the data available to us, due to the unique nature of the intervention as well as the commitment and dedication of the patients receiving treatment. Indeed, this study highlights the dangers of small biotech companies undertaking trials that then become insolvent, a phenomenon seen more extensively in the devices field and termed “neuro‐abandonment.”^14^

## Conclusion

The OXB‐102 trial provides further evidence to support the safety of striatal “dopamine” gene therapies for PD, with possible signals of motor stabilization, though efficacy and dose–response remain unclear due to the trial's early termination and thus incomplete data collection. Future controlled trials that are adequately powered are therefore needed to determine the true therapeutic potential of this approach. Finally, the circumstances underlying the premature discontinuation of this trial also highlight the need for frameworks that ensure both continuity of care and preservation of scientific value when sponsor support fails.

## Author Roles

(1) Research Project: A. Conception, B. Organization, C. Execution; (2) Statistical Analysis: A. Design, B. Execution, C. Review and Critique; (3) Manuscript Preparation: A. Writing of the First Draft, B. Review and Critique.

S.R.: 1C, 2B, 3A.

A.E.: 1C, 2B, 3B.

S.K.: 2B, 3A, 3B.

P.C.B.: 1C, 3B.

R.M.: 1C, 3B.

K.A.: 1C, 3B.

L.S.: 1C, 3B.

S.H.: 1C, 3B.

T.N.: 1C, 3B.

M.H.: 1C, 3B.

K.M.: 1A, 2A, 3B.

L.Z.: 1C, 3B.

M.A.: 1C, 3B.

P.W.: 1C, 3B.

S.M.: 1C, 3B.

P.L.: 1C, 3B.

C.G.: 1C, 3B.

T.F.: 1C, 2C, 3B.

S.P.: 1A, 1C, 2C, 3B.

R.B.: 1A, 1B, 1C, 2A, 2C, 3B.

## Financial Disclosures and Conflicts of Interest

Author disclosures are available in the Supporting Information.

## Supporting information


**Data S1.** Supporting Information.

## Data Availability

The data that support the findings of this study are available on request from the corresponding author. The data are not publicly available due to privacy or ethical restrictions.
