# Supplementary material for: Observations on an Open‐Label Phase 1/2 Dopamine Gene Therapy Trial (OXB‐102/Axo‐Lenti‐PD) in People with Parkinson's Disease
Source: Mov Disord. 2026 May 28;41(9):2490–5. doi: 10.1002/mds.70378 (PMC13602281; doi:10.1002/mds.70378)
Supplement: Supplementary file 1 [file MDS-41-2490-s001.pdf]

CLINICAL RESEARCH PROTOCOL

Protocol Number OXB-102-01

A Phase I/II Safety and Dose Evaluation Study of OXB-102 (AXO-Lenti-PD) in Patients with Bilateral Idiopathic Parkinson's Disease (SUNRISE-PD)

Protocol History:
Original Protocol: 07 March 2016
Protocol Amendment 1: 25 April 2016
Protocol Amendment 2: 06 November 2017
Protocol Amendment 3: 20 April 2018
Protocol Amendment 4: 13 December 2018
Protocol Amendment 5: 04 March 2019
Protocol Amendment 6: 03 September 2019
Protocol Amendment 7: 08 April 2020
Protocol Amendment 8: 03Jun2020

Application Number:
IND number: Not applicable
EudraCT number: 2015-002717-29

**Sponsor**	**Medical Monitor**
**Axovant Sciences**<br>Viaduktstrasse 8<br>4051 Basel<br>Switzerland<br>Tel: +41 43 215 59 99	**Dr Gosse Bruinsma**<br>Axovant Sciences, Inc.<br>640 8th Ave<br>New York, NY 10036<br>USA<br>Mobile: +1 613-866-9609<br>Email: gosse.bruinsma@axovant.com

This confidential document is the property of Axovant Sciences. No unpublished information contained herein may be disclosed without the prior written approval of Axovant Sciences.

PROTOCOL AMENDMENT CHANGE HISTORY

SUMMARY OF MAIN CHANGES

**V9.0 (Amendment 8) 03 June 2020**	
**Summary and Justification of Change**	**Sections Changed**
Title page	The medical monitor information was updated
Dose escalation procedure description was updated to further clarify it and to better align with the SMC charter. The number of subjects in Cohort 3 was updated to 2 to 6 subjects.<br>It was clarified that the Sponsor will determine the optimal safe dose suitable for further evaluation in Part B by reviewing all available data for safety and signs of activity.	Section 4 Study flow diagram<br>Section 8 Study Design<br>Table 6 Dosing regimen

**V8.0 (Amendment 7) 08 April 2020**	
**Summary and Justification of Change**	**Sections Changed**
List of abbreviation was updated to reflect protocol changes	List of Abbreviations
Approval signature page signatories and/or titles were updated to reflect current responsible staff and title	Approval signature page
The drug name OXB-102 was replaced with AXO-Lenti-PD, which is the name assigned upon transfer of rights to Axovant Sciences.<br>Minor edits to correct grammar and enhance clarity of text were applied throughout.	Across the protocol text
The title of the Data Monitoring Committee (DMC) was revised to Safety Monitoring Committee (SMC) to better describe the committee responsibility of evaluating ongoing study safety, without evaluating treatment efficacy. The level of responsibility of the Committee, as outlined in the SMC Charter, is not changed.	List of Abbreviations<br>Section 2 Protocol Summary<br>Section 12 Safety Monitoring Committee (SMC)<br>Other occurrences throughout the protocol
UPDRS area under the curve (AUC), Patient Global Impressions (PGI) Scale and non-motor symptom assessments (NMSS) were added as exploratory evaluations to better understand the potential impact of AXO-Lenti-PD.	List of Abbreviations<br>Section 2 Protocol Summary<br>Section 5.6.3 Rationale for Key Endpoints<br>Section 7.4 Exploratory Endpoints

The use of movement recording device and associated exploratory endpoint were removed due to limited usefulness of data and high patient burden.<br>Secondary endpoints order was revised to better reflect data priorities	Section 8.4.9.4 Dose of L-DOPA for Area Under the Curve (AUC) assessments (at participating sites)<br>Section 9 Study Procedures<br>Section 13.8.3 Exploratory Endpoints<br>Table 1 Schedule of Evaluations
Dose level groupings were clarified, streamlined and are now referred to as 'cohorts'. Low dose, or Dose Level 1 (DL1), is now referred to as Cohort 1; Medium dose, or Dose Level 2 (DL2), is now referred to as Cohort 2; High dose, or Dose Level 3 (DL3), is now referred to as Cohort 3.	Section 2 Protocol Summary<br>Section 4 Study flow diagram<br>Section 5.6.4 Rationale for Dose<br>Section 8 Study Design<br>Section 10 Investigational Medicinal Product and Treatment
Previous target doses that were based on target drug titers are now replaced with actual doses that are based on measured titers. In addition, the infusion volumes are now clearly specified. In order to provide greater putamenal coverage, and potentially greater vector incorporation and, in turn, to achieve better efficacy, the high dose (Cohort 3) total infusion volume was increased from 600 µL (i.e. 100 µl per each of the six infusion sites) to 1800 µL (i.e. 300 µl per each of the six infusion sites). The number of subjects to be treated in Cohort 3 was updated to 2 subjects.	Section 2 Protocol Summary<br>Section 4 Study flow diagram<br>Section 5.6.4 Rationale for Dose<br>Section 5.3.3 Table 2 Composition of AXO-Lenti-PD drug products (all strengths)<br>Section 8 Study Design<br>Table 7 Dosing regimen
The infusion rate for cohort 3 was increased from 3 µL/min to 5 µL/min to reduce operative anaesthesia time.	Table 1 Schedule of Evaluations<br>Section 5.6.5 AXO-Lenti-PD administration<br>Section 8 Study Design<br>Section 10.5 IMP administration for Part A and Part B
Nonclinical study information and data was added to provide rationale for a higher flow rate and volume	Section 5.4 Summary of nonclinical studies
Added vector shedding analysis and endpoint to better characterize the effect of vector administration	Section 13.8 Other analyses
Inclusion criteria –<br>Details of concordance testing criteria was added to previously documented Hauser Diary training procedure in order to ensure that subjects are able to properly identify their on/off state.<br>Added the requirement for clearance by the Enrollment Authorization committee (EAC)	Section 2 Protocol Summary<br>Section 8.3.1 Inclusion Criteria<br>Section 9.2.4 Hauser patient diary training completion and concordance training<br>Section 9 Study Procedures

Exclusion criteria –<br>Exclusion criterion 2 was adjusted to exclude patients who are found to be unwilling to adhere to their prescribed medication requirement<br>History of malignancy exclusion was clarified to except in-situ non-melanoma skin cancer<br>A note was added to the exclusion of disabling dyskinesia, that the criterion should not be shared with the patient during screening	Section 2 Protocol Summary<br>Section 8.3.2 Exclusion Criteria
Study stopping criteria and potentially stopping event handling procedure were clarified	Section 8.4.8 Study stopping criteria
The screening period was extended from 28 to 60 days and was adjusted to better accommodate the time required to schedule the various baseline imaging procedures and to allow the enrollment authorization committee (EAC) sufficient time to review the data. The timing of invasive procedures was adjusted to be performed after EAC clearance.<br>Other minor adjustments were applied to the table of assessments be consistent with the text and to optimize data collection while minimizing burden on the patient	Table 1 Schedule of Evaluations<br>Section 9 Study Procedures
Assessment for long-term follow up years 10 to 15 years were clarified	Table 1 Schedule of Evaluations<br>Section 9 Study Procedures
Handling of changes to concomitant medication for Parkinson’s disease during the study were clarified, to ensure minimal interference with assessments of study drug effect, while ensuring patient safety and comfort	Section 8.4.9.1 Concomitant Treatment with Parkinson’s disease therapies and/or surgical procedures
To ensure patient safety and minimize bleeding during the surgical procedures, a requirement was added that patients must avoid blood thinning medication, including Aspirin, prior to the procedure.	Section 10.5.1 Hospitalisation
Added the specific causal relationship of an adverse event, which was previously not included inadvertently	Section 11.5 Relationship of an adverse event
Deleted redundant section on safety measures, since they are covered by the stopping criteria	Section 11.9 Reporting a serious adverse event
Deleted Appendix 3 of protocol amendment changes to minimize redundancy with previously provided amendment information	Not applicable
Adjusted the reference list to reflect changes to the text	Section 18

Amendment 6, 03 September 2019	
**Summary and Justification of Change**	**Sections Changed**
Sexual abstinence as an acceptable contraceptive method has been clarified according to the MHRA feedback.	Section 2 Protocol Summary<br>Section 8.3.1 Inclusion Criteria
An Enrolment Authorisation Committee (EAC) has been added to review and approve each subject prior to OXB-102 administration or ISP, ensuring that each subject meets entry criteria, has the correct diagnosis, and in addition, has data that are consistent and is a suitable candidate for the study.	8.3 Patient Selection
The protocol has been clarified that the “OFF” time inclusion criterion must NOT be shared with patients or their caregivers prior to and during Screening.	Section 2 Protocol Summary<br>Section 8.3.1 Inclusion Criteria
For women of childbearing potential participating in the PET sub-study, urine pregnancy test has been added to each PET visit at the PET centre as it is a Standard Operating Procedure to ensure that pregnant women will not be scanned.	Section 3 Study Schedule<br>Section 9.1.1 Informed Consent and Screening<br>Section 9.1.3.4 Follow-up at Months 3, 6 and 12 post-surgery<br>Section 9.2.9.1 Striatal dopaminergic changes assessed using PET
The indicated number of sites has been removed including details of investigators	Cover page<br>Section 2 Protocol Summary
Medical Monitor has been changed for the study	Cover Page

Amendment 5, 04 March 2019	
**Summary and Justification of Change**	**Sections Changed**
Inclusion Criteria have been changed as follows to select a wider range of Parkinson’s disease patients who are likely to show benefits in outcome measures assessed with the Hauser patient diary:<br>• Added OFF time of at least 2.5 hours for each day confirmed by the Hauser patient diary and required	Section 2 Protocol Summary<br>Section 8.3.1 Inclusion Criteria

Amendment 5, 04 March 2019	
**Summary and Justification of Change**	**Sections Changed**
patients be able to accurately recognise their "ON" and "OFF" states and be compliant<br>• Reduced L-DOPA Equivalent Daily Dose (LEDD) threshold from 1100 mg to 900 mg<br>• Removed requirement of disease duration of bilateral idiopathic PD for at least 5 years<br>• Reduced lower age limit from 48 to 30<br>• Included women with childbearing potential if they are not pregnant and agree to practice two effective methods of birth control<br>• Reduced threshold of positive response to dopaminergic therapy from 50% to 40% in improvement in UPDRS Part III between the "OFF" and "ON" medication states	
The exploratory endpoint evaluating the percentage change from baseline in the dopamine receptor numbers/activity using PET with [^{11}C]-Raclopride has been revised to be performed with a different PET radioligand, [^{11}C]-(+)-PHNO, because it is available in the UK and has higher sensitivity to changes in dopamine levels than [^{11}C]-Raclopride.	Section 2 Protocol Summary<br>Section 3 Study Schedule<br>Section 5.6.3 Rationale for Key Endpoints<br>Section 5.6.7 Summary of Overall Risks and Benefits<br>Section 6.3 Exploratory Objectives<br>Section 7.4 Exploratory Endpoints<br>Section 8.4.9.2 Withdrawal of PD Medications for "OFF"/"ON" Clinical and PET Assessments<br>Section 9.1.1 Informed Consent and Screening<br>Section 9.1.3.4 Follow-up at Months 3, 6 and 12 post-surgery<br>Section 9.2.9.1 Striatal dopaminergic changes assessed using PET<br>Section 9.2.9.2 MRI / CT scanning at Investigational Sites<br>Section 13.8.3 Exploratory Analyses<br>Appendix 2 Positron Emission Tomography scans<br>Section 18 References

Amendment 5, 04 March 2019	
**Summary and Justification of Change**	**Sections Changed**
Description of Investigational Medicinal Product (IMP) packaging and labelling has been modified to reflect recent changes and simplified for clarity.	Section 8.4.2 Blinding for Part B only<br>Section 10.1 OXB-102 Supply and Labelling<br>Section 10.2 Storage and Dispensing of Study Medication<br>Section 10.5.5 OXB-102 Preparation
The protocol has been amended to allow dilution of high dose IMP on site to create medium or low doses if necessary.	Section 10.5.5 OXB-102 Preparation
Enrolment of patients in the Medium Dose cohort and High Dose cohort has been changed to require the first patient be observed for at least 28 days prior to enrolling the remaining patients of the cohort in parallel to facilitate enrolment with acceptable short-term safety and tolerability in one patient.	Section 8.1 Overview of Study Design
For the Medium Dose cohort and High Dose cohort, minimum data requirement for Data Monitoring Committee (DMC) review has been change to 3 months data for all patients at that dose level only without the requirement of 6 months data for the first three patients to facilitate more timely safety review.	Section 8.1 Overview of Study Design
Collection duration of Hauser patient diary for a visit has been modified to 3 consecutive days from 2 concurrent days and collection windows have been defined more clearly to ensure high data quality for important outcome measures assessed by the diary.	Section 3 Study Schedule<br>Section 9.1.1 Informed Consent and Screening<br>Section 9.1.3 Post-Surgery Follow-up Period<br>Section 9.2.4 Hauser Patient Diary Completion and Review
Overall observation period has been changed from 10 years to 15 years to comply with the US Food and Drug Administration (FDA) guidance.	Section 2 Protocol Summary<br>Section 3 Study Schedule<br>Section 4 Study Flow Diagram<br>Section 5.6.6 Rationale for Study Duration<br>Section 8.1 Overview of Study Design<br>Section 8.2 Schedule of Assessments

Amendment 5, 04 March 2019	
**Summary and Justification of Change**	**Sections Changed**
	and Study Duration<br>Section 8.4.9 Concomitant Medication or Treatment (including any surgical procedures)<br>Section 13.9 Timing Analysis
The Columbia Suicide Severity Rating Scale (C-SSRS) has been added to prospectively assess suicidality (suicidal ideation and behaviour) in accordance with the US FDA guidance. And significant suicide risk has been added to Exclusion Criteria to establish appropriate baseline for assessing suicidality post treatment.	Section 2 Protocol Summary<br>Section 3 Study Schedule<br>Section 8.3.2 Exclusion Criteria<br>Section 9.1.1 Informed Consent and Screening<br>Section 9.1.3 Post-Surgery Follow-up Period<br>Section 9.2.10 Other Measures<br>Section 11.2 Procedures for Recording Adverse Events
Visit windows for the Month 6 and Month 9 visits have been changed from ± 7 days to ± 14 days to improve feasibility and compliance in study protocol. And visit window for Screening was clarified.	Section 3 Study Schedule
Urine pregnancy test has been added for women of childbearing potential to assess eligibility and pregnancy post treatment.	Section 3 Study Schedule<br>Section 9.2.1 Clinical Laboratory Tests
Some DMC tasks that should belong to the sponsor have been removed to lessen the burden on the DMC. In addition, the DMC role of making recommendation to the study has been clarified.	Section 2 Protocol Summary<br>Section 4 Study Flow Diagram<br>Section 8.1 Overview of Study Design<br>Section 12 Data Monitoring Committee (DMC)<br>Section 13.11 Data Monitoring Committee Data Review
The current code name of the IMP, AXO-Lenti-PD, has been added to the protocol.	Section 2 Protocol Summary<br>Section 10.1 OXB-102 Supply and Labelling
Title of the study has been updated to include the current code name of the IMP, AXO-Lenti-PD, and the study	Cover Page

Amendment 5, 04 March 2019	
**Summary and Justification of Change**	**Sections Changed**
brand name, SUNRISE-PD.	Section 2 Protocol Summary
Safety data of ProSavin® have been updated with data cut-off date of 11th December 2018 from Study PS1/001/09.	Section 5.4.6 Effects in Humans<br>Section 5.6.7 Summary of Overall Risks and Benefits
Re-screening condition for patients who screen fail due to criteria related to Hauser patient diary has been defined.	Section 8.4.4 Replacement/Re-screening of Patients
Optional video recording of patients for UPDRS Part III and Dyskinesia Scale has been indicated for additional visits at Month 3 and Month 9. And language regarding how the video will be used has been removed as it may not be accurate.	Section 3 Study Schedule<br>Section 9.1.3 Post-Surgery Follow-up Period<br>Section 9.2.6 Questionnaires<br>Section 13.1 Statistical Analysis Plans

Amendment 4, 14 December 2018	
**Summary and Justification of Change**	**Sections Changed**
Change of sponsorship from Oxford Biomedica (UK) Ltd. to Axovant Sciences	Cover Page Sponsor and Confidentiality Statement<br>Section 2 Protocol Summary and throughout as necessary

Amendment 3, 20 April 2018	
**Summary and Justification of Change**	**Sections Changed**
The exploratory endpoint evaluating the percentage change from baseline in the dopamine receptor numbers/activity using PET with ^{11}C-Raclopride has been revised to be performed only on patients participating in France due to issues with availability of the radioligand in the UK.	Section 2 Protocol Summary<br>Section 3 Study Schedule<br>Section 5.6.3 Rationale for Key Endpoints<br>Section 6.3 Exploratory Objectives<br>Section 7.4 Exploratory Endpoints<br>Section 9.1.1 Informed Consent and Screening<br>Section 9.1.3.4 Follow-up at Months 3, 6 and 12 post-surgery<br>Section 9.2.9.1 Striatal dopaminergic changes assessed using PET<br>Appendix 2 Positron Emission Tomography scans
Update of cumulative safety data on ProSavin® clinical studies in line with DSUR dated 25 January 2018:	Section 5.4.6 Effects in Humans<br>Section 5.4.6.1.1 Safety<br>Section 5.6.7 Summary of Overall Risks and Benefits
A revision of the calculation transposing non-clinical doses in the non-human primate (NHP) to clinical doses taking into account a recent independant review on appropriate allometric scaling. This change affords a larger safety margin for the proposed clinical doses which remain unchanged.	Section 5.4.2.1 Evaluation of Efficacy<br>Section 5.4.4 Nonclinical Toxicology<br>Section 5.6.4 Rationale for Dose<br>Section 5.6.7 Summary of Overall Risks and Benefits

Clarification of the anticipated potency of OXB-102 in the clinic.	Section 5.4.1 Summary of Nonclinical Studies<br>Section 5.6.4 Rationale for Dose<br>Table 2 Dose Escalation in OXB-102-01<br>Section 5.6.7 Summary of Overall Risks and Benefits
Additional flexibility with patient recruitment during the low dose cohort in part A of the study from 'up to 6 patients (minimum 4 patients)' to reduce the minimum number of patients to 2 patients. Additionally, the DMC recommendations on this low dose cohort will be based on review of the primary safety endpoint data (3 months minimum follow up data) plus all available activity data at that point (rather than 6 months activity data). This has been aligned in accordance with the clarification of anticipated potency in the rationale for dose of OXB-102 in Section 5.6.4 to avoid unnecessary patients being treated with potentially sub-optimal or sub-therapeutic doses.	Section 4 Study Flow Diagram<br>Section 5.6.7 Summary of Overall Risks and Benefits<br>Section 8.1 Overview of Study Design<br>Table 3 Dosing Regimen
Update to IMP labelling to include an expiry date on secondary and tertiary packaging instead of a retest date.	Section 10.1 OXB-102 Supply and Labelling

Amendment 2, 03 November 2017	
**Summary and Justification of Change**	**Sections Changed**
Change in administration details:<br>• Addition of investigative site: Principal Investigator: Prof. Thomas Foltynie, The National Hospital for Neurology and Neurosurgery, London, UK<br>• Change in Sponsor Statistician: Lothar Tremmel replaced Peter Treasure	Title Page<br>Section 2 Protocol Summary<br>Approval Signature Page
Update to details of IMP:<br>• Appearance of IMP inserted<br>• Further details added to rationale for dose to link to first generation product (ProSavin®) dosing<br>• New details for labelling, packaging and update to the number of IMP vials required per patient due to the change in the administration system<br>• Removal of need to place IMP on cold blocks during surgical procedure due to new stability data	Section 5.2.2 Description<br>Section 5.5.4 Rationale for Dose<br>Section 10.1 OXB-102 Supply and Labelling<br>Section 10.5.5 OXB-102 Preparation<br>Section 10.5.7 OXB-102 Administration System
Update of cumulative safety data on ProSavin® clinical studies in line with DSUR dated 25 January 2017:	Section 5.3.6 Effects in Humans<br>Section 5.3.6.1.1 Safety<br>Section 5.5.7 Summary of Overall Risks and Benefits
Inclusion of details on the OXB-102 Administration System including:<br>• Rationale for use including the regulatory guidance, regulations and ISO standards being followed specifically for the administration system<br>• Update of Study Stopping Criteria to include safety events specifically related to the administration system<br>• Update of surgical procedures using the new administration system<br>• Safety reporting details for administration system including reporting of device deficiencies (including management of safety information for the system during the blinded part B of the study).	Section 5.5.5 Rationale for OXB-102 Administration System<br>Section 5.5.7 Summary of Overall Risks and Benefits<br>Section 15.1 Good Clinical Practice<br>Section 8.4.8 Study Stopping Criteria<br>Section 10.5.7 OXB-102 Administration System<br>Section 8.4.2.4 Unblinding of AEs concerning the OXB-102 Administration System<br>Section 11 Assessment of Safety (throughout)

Flexibility with patient recruitment during dose escalation part A of the study from 6 patients per dose cohort to 'up to 6 patients' (minimum 4 patients); to be determined by the DMC to avoid unnecessary patients being treated should data on reduced patient numbers be clear for DMC recommendations.	Section 2 Protocol Summary<br>Section 4 Study Flow Diagram<br>Section 8.1 Overview of Study Design<br>Table 3 Dosing Regimen

Amendment 1, 25 April 2016	
**Summary and Justification of Change**	**Sections Changed**
Modifications made in response to the MHRA during their initial review of the protocol regarding:<br>a) clarification and addition of further study stopping criteria	Section 8.1 Overview of Study Design<br><br>Section 8.4.8 Study Stopping Criteria
b) description of the control of patient registration into Part A of the study across participating sites	Section 8.4.1 Treatment Assignment<br><br>Section 9.1.1 Informed Consent and Screening<br><br>Section 10.1 OXB-102 Supply and Labelling<br><br>Section 10.2 Storage and Dispensing of Study Medication<br><br>Section 10.5.5 OXB-102 Preparation
c) description of how safety data is disseminated to all participating sites	Section 8.1 Overview of Study Design<br><br>Section 11.3 Severe Adverse Events
Clarification of DMC responsibilities.	Section 12 Data Monitoring Committee (DMC)

APPROVAL SIGNATURE PAGE

DocuSigned by:

Gavin Corcoran

Signer Name: Gavin Corcoran
Signing Reason: I approve this document
Signing Time: 09-Jun-2020 | 13:42 EDT
8DE3971851864B488366B8F381E6712C

09-Jun-2020 | 13:42 EDT

Gavin Corcoran, MD — Date

Chief R&D Officer

Axovant Sciences

DocuSigned by:

Elimor Brand-Schieber

Signer Name: Elimor Brand-Schieber
Signing Reason: I approve this document
Signing Time: 09-Jun-2020 | 13:47 EDT
29A381C35D9E417187DD058527FAC2CE

09-Jun-2020 | 13:47 EDT

Elimor Brand-Schieber, PhD — Date

Senior Director, Clinical Research

Axovant Sciences

TABLE OF CONTENTS

LIST OF TABLES 20
LIST OF ABBREVIATIONS 21
1 INVESTIGATOR STATEMENT 26
2 PROTOCOL SUMMARY 27
3 STUDY SCHEDULE 33
4 STUDY FLOW DIAGRAM 39
5 INTRODUCTION AND RATIONALE 40
5.1 Background to Parkinson's Disease 40
5.2 Background to AXO-Lenti-PD 41
5.3 Description of Product 42
5.3.1 Introduction 42
5.3.2 Description 43
5.3.3 Composition of the Medicinal Product 43
5.4 Summary of Pre-Clinical Studies 44
5.4.1 Summary of Nonclinical Studies 44
5.4.2 Nonclinical Pharmacology 46
5.4.3 Nonclinical Pharmacokinetics 46
5.4.4 Nonclinical Toxicology 47
5.4.5 Carcinogenicity 48
5.4.6 Effects in Humans 50
5.5 Rationale for Study Conduct 52
5.6 Rationale for Study Design, Doses, and Control Groups 52
5.6.1 Rationale for Study Design 52
5.6.2 Rationale for Study Population and Disease Stage 53
5.6.3 Rationale for Key Endpoints 54
5.6.4 Rationale for Dose 55
5.6.5 AXO-Lenti-PD Administration 57
5.6.6 Rationale for Study Duration 58
5.6.7 Summary of Overall Risks and Benefits 58
6 STUDY OBJECTIVES 62
6.1 Primary Objectives 62

6.2 Secondary Objectives 62
6.3 Exploratory Objectives 62
7 STUDY ENDPOINTS 63
7.1 Primary Endpoint 63
7.2 Secondary Endpoints 63
7.3 Biodistribution Endpoint 63
7.4 Exploratory Endpoints 64
8 STUDY DESIGN 65
8.1 Overview of Study Design 65
8.2 Schedule of Assessments and Study Duration 69
8.2.1 Part A 69
8.2.2 Part B 69
8.3 Patient Selection 70
8.3.1 Inclusion Criteria 70
8.3.2 Exclusion Criteria 72
8.4 Patient Enrolment and Randomisation 74
8.4.1 Treatment Assignment 74
8.4.2 Blinding for Part B only 75
8.4.3 Withdrawal Criteria 76
8.4.4 Replacement/Re-screening of Patients 77
8.4.5 Patient Completion 77
8.4.6 End of the Study 77
8.4.7 Annual Follow-up for Survival 78
8.4.8 Study Stopping Criteria 78
8.4.9 Concomitant Medication or Treatment (including any surgical procedures) 78
9 STUDY PROCEDURES 81
9.1 Schedule of Study Procedures 81
9.1.1 Informed Consent and Screening 81
9.1.2 Treatment Period 84
9.1.3 Post-Surgery Follow-up Period 85
9.2 Details of Specific Procedures 93
9.2.1 Clinical Laboratory Tests 93
9.2.2 Samples for Biodistribution, Immunology, Biomarkers and Pharmacogenomics 94

9.2.3 Physical Examination, Vital Signs and Other Observations related to Safety 95
9.2.4 Hauser Patient Diary Training, Completion and Concordance Testing .. 96
9.2.5 Questionnaires 96
9.2.6 Neurological Examination 99
9.2.7 L-DOPA and PD therapy 99
9.2.8 Imaging 99
9.2.9 Other Measures 101
10 INVESTIGATIONAL MEDICINAL PRODUCT AND TREATMENT 102
10.1 AXO-Lenti-PD Supply and Labelling 102
10.2 Storage and Dispensing of Study Medication 103
10.3 IMP Accountability and Destruction 104
10.4 Treatment Regimen 104
10.5 IMP Administration for Part A and Part B 104
10.5.1 Hospitalisation 104
10.5.2 General Anaesthesia 105
10.5.3 Attachment of Stereotactic Head Frame 105
10.5.4 Preparation of Surgical Site 105
10.5.5 AXO-Lenti-PD Preparation 105
10.5.6 Locating Sites for AXO-Lenti-PD Administration 106
10.5.7 AXO-Lenti-PD Administration System 106
10.5.8 Post-Surgical Monitoring 108
10.6 Differences in Procedures for Part B Patients 109
11 ASSESSMENT OF SAFETY 110
11.1 Adverse Event Definition 110
11.1.1 For IMP 110
11.1.2 For AXO-Lenti-PD Administration System 110
11.2 Procedures for Recording Adverse Events 110
11.3 Abnormal Laboratory Values 111
11.4 Abnormal Vital Sign Values 111
11.5 Relationship of an Adverse Event 112
11.5.1 For IMP 112
11.5.2 For AXO-Lenti-PD Administration System 112
11.5.3 For Surgical or Study Procedures 112
11.6 Severity of an Adverse Event 113

11.7 Serious Adverse Event Definition 113
11.7.1 For IMP, Surgical or Study Procedures 113
11.7.2 For AXO-Lenti-PD Administration System 114
11.8 Elective Procedures 114
11.9 Reporting a Serious Adverse Event 114
11.10 Reporting Device Deficiencies 115
11.11 Adverse Events of Special Interest (AESIs) 116
11.12 Biopsies 116
12 SAFETY MONITORING COMMITTEE (SMC) 117
13 STATISTICAL CONSIDERATIONS 118
13.1 Statistical Analysis Plans 118
13.2 Data Listings and Summaries 118
13.2.1 Conduct of Analysis 119
13.3 Analysis Populations 119
13.3.1 Safety Analysis Set 119
13.3.2 Activity Evaluable Analysis Set 119
13.4 Missing Data 119
13.5 Study Conduct 119
13.6 Analysis of Safety 119
13.6.1 Safety Endpoints 119
13.6.2 Safety Summaries 120
13.6.3 Adverse Events 120
13.6.4 Duration of Survival 120
13.7 Analysis of Activity Endpoints 120
13.7.1 Activity Endpoints 120
13.7.2 Activity Summaries 121
13.8 Other Analyses 121
13.8.1 Biodistribution and Vector Shedding Analysis 121
13.8.2 Immunology Analysis 122
13.8.3 Exploratory Endpoints 122
13.8.4 Exploratory Analyses 122
13.9 Timing Analysis 122
13.10 Sample Size 123
13.11 Safety Monitoring Committee Data Review 123
13.12 Deviations from the Planned Statistical Analysis 123

14 QUALITY CONTROL AND QUALITY ASSURANCE 124
14.1 Data Collection and Source Documents 124
14.2 Deviations from Protocol and GCP 124
14.3 Study Monitoring and Access to Source Documents 124
14.4 Audit and Inspection of the Study 125
15 REGULATORY AND ETHICAL CONSIDERATIONS 126
15.1 Good Clinical Practice 126
15.2 Investigator Responsibilities 126
15.3 Patient Information and Consent 126
15.4 Confidentiality 127
15.5 Protocol Amendments 127
15.6 Urgent Safety Measures 127
15.7 Trial Approval 128
15.8 Trial Closure 129
15.9 Financial Disclosure 129
16 INSURANCE INDEMNITY 130
17 PUBLICATION POLICY 130
Appendix 1 World Medical Association Declaration of Helsinki 131
Appendix 2 Positron Emission Tomography Scans 131
18 REFERENCES 133

LIST OF TABLES

Table 1: Schedule of Evaluations 33
Table 2 Composition of AXO-Lenti-PD Drug Products (all strengths) 43
Table 3 AXO-Lenti-PD Dosing Parameters 55
Table 4 Scaled Doses and Safety Margins based on NHP Studies 57
Table 5 Scaled Volumes of Infusion (Vi) and Safety Margins based on NHP studies 57
Table 6 Dosing Regimen 68

LIST OF ABBREVIATIONS

Abbreviation or Specialized Term	Definition
AADC	Aromatic L-Amino-acid Decarboxylase
AC	Anterior Commissure
ADL	Activities of Daily Living
AE	Adverse Event
AESI	Adverse Event of Special Interest
AFS	Absorbable Fixation System
ALT	Alanine Aminotransferase
AP	Alkaline Phosphatase
AST	Aspartate Aminotransferase
ATMP	Advanced Therapy Medicinal Product
AUC	Area Under the Curve
BP	Blood Pressure
BUN	Blood Urea Nitrogen
Ca	Calcium
CDS	Continuous Dopamine Stimulation
CFR	Code of Federal Regulations
CGD	Chronic Granulomatous Disease
CGI	Clinical Global Impression
CH1	GTP-Cyclohydrolase
CHOL	Cholesterol
CNS	Central Nervous System
CPK	Creatine Phosphokinase
eCRF	Electronic Case Report Form
CRO	Contract Research Organisation
CRP	C-Reactive Protein
CSF	Cerebrospinal Fluid
C-SSRS	Columbia Suicide Severity Rating Scale
CT	Computer Tomography
CTCAE	Common Terminology Criteria for Adverse Events
DBS	Deep Brain Stimulation

Abbreviation or Specialized Term	Definition
DL	Dose Level
SMC	Data Monitoring Committee
DNA	Deoxyribonucleic Acid
DRS-2	Mattis Dementia Rating Scale–2™
DSUR	Development Safety Update Report
EAC	Enrollment Authorization Committee
EC	Ethics Committee
ECG	Electrocardiogram
EDC	Electronic Data Capture
EIAV	Equine Infectious Anaemia Virus
EMA	European Medicines Agency
EQ-5D-5L	EuroQOL Five Dimensions Questionnaire
ESR	Erythrocyte Sedimentation Rate
EU	European Union
EURO-QOL	EuroQol Group EQ-5D™
EudraCT	European Clinical Trial Database
FBC	Full Blood Count
FDA	U.S. Food and Drug Administration
^{18}F-FMT	6-[^{18}F]Fluoro-L-*m*-tyrosine
g	Gram
GCP	Good Clinical Practice
GLP	Good Laboratory Practice
GGT	Gamma Glutamic Transpeptidase
GPi	Globus Pallidus Internal
HADS	Hospital Anxiety and Depression Scale
hCG	human Chorionic Gonadotropin
HCT	Haematocrit
HDL	High-density Lipoprotein Cholesterol
HGB	Haemoglobin
HIPAA	Health Insurance Portability and Accountability Act
H&Y	Hoehn and Yahr
HR	Heart Rate

Abbreviation or Specialized Term	Definition
DIBD	Development International Birth Date
IB	Investigator Brochure
ICD	Impulse Control Disorder
ICH	International Conference on Harmonization
IND	Investigational New Drug
IEC	Independent Ethics Committee
IMP	Investigational Medicinal Product
IRB	Institutional Review Board
ISP	Imitation Surgical Procedure
IxRS	Interactive Voice and Web Response System
LDH	Lactase Dehydrogenase
LDL	Low-density Lipoprotein Cholesterol
L-DOPA	L-Dihydroxyphenylalanine
LEDD	L-DOPA Equivalent Daily Dose
MCH	Mean Corpuscular Haemoglobin
MCV	Mean Corpuscular Volume
MedDRA	Medical Dictionary for Regulatory Activities
Mg	Milligram
MHRA	Medicines and Healthcare Products Regulatory Agency
μL	Microlitre
mL	Millilitre
mm	Millimetre
μM	Micromolar
MoCA	Montreal Cognitive Assessment
MPTP	N-Methyl-4-Phenyl-1,2,3,4-Tetrahydropyridine
MRI	Magnetic Resonance Imaging
NHP	Non-Human Primate
NMSS	Non-Motor Symptom Scale
6-OHDA	6-Hydroxydopamine
OXB	Oxford BioMedica (UK) Ltd
PC	Posterior Commissure
PCR	Polymerase Chain Reaction

Abbreviation or Specialized Term	Definition
PD	Parkinson's disease
PDQ-39	Parkinson's Disease Questionnaire 39
PDS	Parkinson's Disease Society
PET	Positron Emission Tomography
PGI	Patient Global Impression
PHNO	4-propyl-9-hydroxynaphthoxazine
PI	Principal Investigator
PRO	Patient Reported Outcome
PT	Prothrombin Time
PTT	Partial Thromboplastin Time
qPCR	Quantitative Polymerase Chain Reaction
RTqPCR	Reverse Transcriptase Quantitative Polymerase Chain Reaction
QUIP-RS	Questionnaire for Impulsive-Compulsive Disorders in Parkinson's Disease – Rating Scale
RBC	Red Blood Cells
RCL	Replication Competent Lentivirus
RDRS	Rush Dyskinesia Rating Scale
RDW	Red Cell Distribution Width
RNA	Ribonucleic Acid
SADE	Serious Adverse Device Effect
SAE	Serious Adverse Event
SAP	Statistical Analysis Plan
SAR	Serious Adverse Reaction
SMC	Safety Monitoring Committee
SOC	System Organ Class
STN	Subthalamic Nucleus
SUSAR	Suspected Unexpected Serious Adverse Reaction
TBIL	Total Bilirubin
TH	Tyrosine Hydroxylase
TP	Total Protein
TSH	Thyroid Stimulating Hormone
TSSM	Tromethamine, Sodium chloride, Sucrose and Mannitol

**Abbreviation or Specialized Term**	**Definition**
TU	Transducing Units
UDS	Urine Drug Screen
UK	United Kingdom
UPDRS	Unified Parkinson's Disease Rating Scale
USA	United States of America
Vi	Volume of infusion
VSV-G	Vesicular Stomatitis Virus Glycoprotein
WBC	White Blood Cell
X-SCID	X-Linked Severe Combined Immunodeficiency

1 INVESTIGATOR STATEMENT

I am aware of my responsibilities as an investigator under the guidelines of ICH Good Clinical Practice, The Declaration of Helsinki, EU Directives 2001/20/EC and 2005/28/EC, all applicable laws and regulations, including but without limitation, those for data privacy and Advanced Therapy Medicinal Products. I agree to conduct the study according to these requirements and those of the study protocol and to appropriately direct and assist the staff under my control who will be involved in the study, ensuring they have access to the study protocol and any amendments, the Investigator Brochure and other relevant information and are aware of their obligations.

I, the undersigned, have read, understood and agree with protocol OXB-102-01.

Signed ____

Name ____

Hospital ____

Date ____

2 PROTOCOL SUMMARY

**NAME OF SPONSOR**<br>Axovant Sciences	**EudraCT No.:**<br>2015-002717-29	*IND No.: Not applicable*

INVESTIGATIONAL MEDICINAL PRODUCT (IMP):
AXO-Lenti-PD (OXB-102)

TITLE OF STUDY:
A Phase I/II Safety and Dose Evaluation Study of OXB-102 (AXO-Lenti-PD) in Patients with Bilateral Idiopathic Parkinson's Disease (SUNRISE-PD)

**STUDY NUMBER:**<br>OXB-102-01	**PHASE OF STUDY:**<br>I/II

STUDY DESIGN:
The study design is a two-part, phase I/II study, with the first part (Part A) comprising open label, non-randomised dose escalation of three potential dose levels of AXO-Lenti-PD. The second part of the study (Part B) is a double-blind design with patients randomised either to an active group, receiving the optimal dose from Part A, or a control group, receiving an imitation surgical procedure (ISP).

STUDY OBJECTIVES:
Primary:
- To evaluate the safety and tolerability of AXO-Lenti-PD treatment in patients with bilateral, idiopathic, advanced stage Parkinson's disease (PD)

Secondary:
- To determine the optimal dose of AXO-Lenti-PD
- To evaluate any signs of activity of AXO-Lenti-PD
- To evaluate the long term safety of AXO-Lenti-PD

Exploratory:
- To evaluate any changes in dopamine levels with the use of [^{11}C]-(+)-4-propyl-9-hydroxynaphthoxazine ([^{11}C]-(+)-PHNO) Positron Emission Tomography (PET) non-displaceable binding potential (BP_{ND}) and their correlation with changes in clinical assessments
- To conduct exploratory research on optional blood samples for PD-related research, including but not limited to genomic analysis
- To explore treatment impact on novel questionnaires

PATIENT POPULATION:

Patients aged between 30 and 70 years, with bilateral, idiopathic, advanced stage PD, defined by a Unified Parkinson's Disease Rating Scale (UPDRS) (Part III) score of between 30 and 60 in the "OFF" medication state with motor fluctuations.

NUMBER OF PATIENTS:
Up to 30 (up to 6 per dose level for Cohort 1, Cohort 2, and Cohort 3 in Part A of the study; 12 in Part B, 8 randomised to AXO-Lenti-PD and 4 to ISP).

**DOSE LEVELS:**	**ROUTE OF ADMINISTRATION:**
Patients in Part A will receive one dose of AXO-Lenti-PD at one of the following dose levels (DL):<br>DL1 (Cohort 1): 2.64 x 10^6 TU in 600 µL total infusion volume<br>DL2 (Cohort 2): 9.00 x 10^6 TU in 600 µL total infusion volume<br>DL3 (Cohort 3): 2.70 x 10^7 TU in 1800 µL total infusion volume.<br>Patients randomized to the active group in Part B will receive the optimal dose of AXO-Lenti-PD as determined in Part A of	AXO-Lenti-PD will be infused bilaterally to the posterior putamen of the brain.

the study; those randomized to the control group in Part B will receive an ISP.	
**DURATION OF STUDY / FOLLOW-UP:**<br>Regular assessments during the first 12 months after surgery, every 6 months in the second year, then annual assessments up to 15 years after surgery.	

INCLUSION CRITERIA:

1) Diagnosed with bilateral idiopathic PD, using the UK Parkinson's Disease Society (PDS) Brain Bank Criteria, but without the specific exclusion of patients with more than one affected relative
2) Males/females between 30 and 70 years at the time of surgery
3) Presence of motor fluctuations and/or dyskinetic movement
4) Affiliated with the French, UK or equivalent social security health care system
5) Candidate for surgical intervention because of disabling motor complications and willing to defer this until at least after the 12 month study visit (unless medically recommended)
6) Freely given signed and dated written informed consent obtained from the patient/legal representative prior to performing any protocol-related procedures, including screening evaluations
7) **For Women:** Women of nonchildbearing potential who meet at least 1 of the following criteria:

   a. Achieved postmenopausal status, defined as cessation of regular menses for at least 12 consecutive months with no alternative pathological or physiological cause

   b. Documented hysterectomy with bilateral oophorectomy

   c. Medically confirmed ovarian failure

   Women who are not postmenopausal (as defined above) must have:

   d. Negative pregnancy test and must not be lactating at the Screening and Day -1 visits

   a. Must agree to practice two effective methods of birth control, including at least one barrier method and one highly effective method, and must agree to continue using such contraceptive precautions for at least the first 3 months of the study following AXO-Lenti-PD administration. Highly effective forms of birth control include:

   - Combined (oestrogen and progestogen containing) hormonal contraception associated with inhibition of ovulation: oral, intravaginal, or transdermal
   - Progestogen-only hormonal contraception associated with inhibition of ovulation: oral, injectable, or implantable
   - Intrauterine device (IUD)
   - Intrauterine hormone-releasing system (IUS)
   - Bilateral tubal occlusion
   - Vasectomized partner
   - Sexual abstinence (true abstinence only; periodic abstinence [e.g. calendar, ovulation, symptothermal, post-ovulation methods] and withdrawal are not acceptable)

For Men: Males, unless surgically sterile, must agree in conjunction with their female partner to use two effective methods of birth control if their partner is of childbearing potential, including at least one barrier method, and must agree to continue using such contraceptive precautions for at least the first 3 months of the study following AXO-Lenti-PD administration

8) Willing to be hospitalised and have current PD treatment withdrawn for up to 24 hours (48 hours for long lasting agonists) for each visit where patients need to be assessed in the “OFF” medication state
9) UPDRS Part III score of between 30 and 60 in the “OFF” medication state
10) Positive response to dopaminergic therapy as defined by at least a 40% improvement in UPDRS (Part III) between the “OFF” and “ON” medication states at Screening
11) Hoehn and Yahr (H&Y) Stage 3 or 4 in the “OFF” medication state at Screening
12) Stable dosing of PD medication, including L-DOPA, for four weeks prior to the first day of Screening with the L-DOPA Equivalent Daily Dose (LEDD) being ≥ 900 mg
13) Experiencing at least 2.5 hours of “OFF” time confirmed by the Hauser patient diary for each of 3 consecutive days anytime during Screening. Patients must demonstrate ability to accurately recognise their “ON” and “OFF” states after appropriate training as verified by successfully meeting concordance testing criteria. Patients must be compliant (with no missing data > 2 hours per day) for at least 2 of the 3 consecutive days for the Screening visit. The “OFF” time inclusion criterion must NOT be shared with patients or their caregivers prior to and during Screening
14) Clearance by an Enrollment Authorization Committee (EAC) comprised of Parkinson’s disease clinical experts

EXCLUSION CRITERIA:

1) Any condition that, in the opinion of the investigator and/or EAC, would interfere with evaluation of the IMP or interpretation of patient safety or study results
2) Patients unable or unwilling to adhere to their prescribed PD medication regimen or the requirements of the study in the opinion of the investigator
3) Life-threatening illness unrelated to PD at the time of surgery
4) History of malignancy within five years or a positive cancer screening test within one year prior to screening visit (with the exception of in situ, non-melanoma forms of skin cancer)
5) History of epilepsy or any other co-morbid condition that the Investigator believes presents an unacceptable health risk to the patient in conjunction with the procedures in this protocol
6) History of psychosis or current treatment with dopamine blocking agents of any kind and prior regular exposure to antipsychotic agents
7) Concurrent treatment for depression that targets the dopaminergic pathways.
8) Concurrent anti-retroviral therapy that would inactivate the investigational agent
9) Alcohol or other substance abuse within five years prior to screening visit
10) History of stereotactic or other surgery for the treatment of PD, including Deep Brain Stimulation (DBS)
11) Participation in a prior cell or gene transfer therapy study
12) Planned participation in any other clinical study, for any condition, including those relating to PD, during the first 12 months of the study
13) Treated with dopaminergic antagonists within six months prior to screening
14) Prior treatment with tolcapone, within the six months prior to screening, due to its ability to modify dopaminergic pathways within the brain
15) History of treatment with any agent that may induce PD or PD symptoms within the last three months prior to screening
16) Any contraindication for undergoing a Magnetic Resonance Imaging (MRI) scan of the head
17) Contraindications to use of anaesthesia
18) Current or anticipated treatment with anticoagulant therapy or the use of anticoagulation therapy that cannot be temporarily stopped around the time of surgery
19) Any known allergy to any component of the IMP or protocol required medication
20) Severe disabling dyskinesia’s ≥ 51% of the day as defined by the UPDRS (Part IV) at Screening
    - Note: The dyskinesia time exclusion criterion must NOT be shared with patients or their caregivers prior to and during Screening

21) Patients with L-DOPA resistant tremor
22) Diagnosis of multiple system atrophy following assessment of the autonomic nervous system function and MRI during the screening process (historical diagnosis not formal testing)
23) Abnormal MRI findings such as mega cisterna, septum pellucidum, signs of severe cortical or subcortical atrophy, brain tumours, vascular diseases, trauma or arteriovenous malformations
24) Clinically significant abnormalities in laboratory tests (including full blood count, chemistry panel, liver function tests), electrocardiogram (ECG) and/or chest X-ray, identified during the screening period that in the opinion of the investigator would make the patient unsuitable for participation in the study
25) Presence of dementia, as diagnosed using the Mattis Dementia Rating Scale (2nd Edition)
26) Intercurrent illness or severe infection 28 days prior to AXO-Lenti-PD administration
27) Major surgery within the 28 days prior to AXO-Lenti-PD administration
28) History of any investigational agent within 3 months prior to AXO-Lenti-PD administration
29) Inability to understand the informed consent or to comply in full with the requirements of the study
30) Significant suicide risk as defined by
    - Suicidal ideation as endorsed on items 4 or 5 on the Columbia Suicide Severity Rating Scale (C-SSRS) within the past year at Screening of this study, or
    - Suicidal behaviours within the past year endorsed on the C-SSRS at Screening of this study, or
    - Clinical assessment of significant suicidal risk

MAIN ENDPOINTS:

Primary endpoints:

- The incidence, causality and severity of adverse events, and measurements of other safety parameters (laboratory parameters, vital signs, brain MRI, physical examination, C-SSRS) at 3 months

Secondary endpoints:

The primary time point for secondary endpoints is 6 months unless otherwise specified.

- The change in UPDRS scores for each part compared to baseline in defined “OFF” and “ON” medication states
- The change in dyskinesia rating scale from baseline using the Rush Dyskinesia Rating Scale (RDRS)
- The change in time during the waking day the patient is in the “OFF” state compared to baseline as assessed by Hauser patient diaries
- The change from baseline in the patients’ total score on activity of daily living using the PD Questionnaire 39 (PDQ-39)
- The change in health-related quality of life using the EuroQOL five dimensions questionnaire (EQ-5D-5L)
- The change from baseline in impulsivity using the Questionnaire for Impulsive-Compulsive Disorders – Rating Scale in Parkinson's Disease (QUIP-RS)
- The change in Clinical Global Impression (CGI) Scale from baseline
- The change from baseline in dementia using the Mattis Dementia Rating Scale–2™ (DRS-2)
- The change from baseline in the Hoehn and Yahr Scale in “ON” and “OFF” states
- The change from baseline in cognition using the Montreal Cognitive Assessment (MoCA)
- The change from baseline in depression/mood using the Hospital Anxiety and Depression Scale (HADS)
- The change from baseline in apathy using the Starkstein Apathy Scale
- The change from baseline in the L-DOPA Equivalent Daily Dose (LEDD)
- The change from baseline in L-DOPA dose
- Immunology: the incidence of humoral responses to AXO-Lenti-PD administration

Biodistribution Endpoint

- Potential distribution in the blood and urine of AXO-Lenti-PD as assessed by quantitative polymerase chain reaction (qPCR) analysis

Exploratory Endpoints

- The percentage change from baseline in the dopamine levels using [^{11}C]-(+)-PHNO PET BP_{ND} at "OFF" state and optional at "ON" medication state
- Correlation between the percentage change from baseline in the dopamine levels using [^{11}C]-(+)-PHNO PET BP_{ND} at "OFF" state and various clinical endpoints including the percentage change from baseline in UPDRS scores at "OFF" state
- The change from baseline in the Non-Motor Symptom Scale (NMSS)
- The change in Patient Global Impression (PGI) Scale from baseline
- Exploratory research on optional blood samples for PD-related research, including but not limited to genomic analysis

STATISTICAL CONSIDERATIONS:

Treatment Groups

Treatment Groups are defined as follows:

Treatment Group	Part	Treatment
1	A	Dose Level 1
2	A	Dose Level 2
3	A	Dose Level 3
4	B	Optimal dose from Part A
5	B	Imitation Surgical Procedure

Listings and Summaries

All electronic Case Report Form (eCRF) data and derived study endpoints will be listed. Listings will be sorted by Treatment Group, Patient Number and Time point.

The standard summary statistics for categorical variables are the number and proportion (expressed as percentage) of patients in each category. The standard summary statistics for continuous variables are mean, standard deviation, median, minimum and maximum.

Baseline characteristics will be summarised by Treatment Group. Patient disposition will be summarised by Treatment Group and Time point. Study endpoints (safety, activity and other), together with, if appropriate, absolute and/or percentage change from baseline will be summarised by Treatment Group and Time point. A pooled summary will also be provided for Treatment Groups 1 to 4 inclusive (all treated patients). An additional summary will be provided pooling Treatment Group 4 and the corresponding Dose Level from Part A.

Figures may be generated as appropriate.

Primary Time points

The primary time point for the assessment of safety data is three months after surgery. The primary time point for the assessment of activity data is six months after surgery. The first analyses to be reported in the Clinical Study Report (CSR) will be performed when all patients have reached six months after surgery or have withdrawn before six months.

Follow-up Time points

During the follow-up period (from the last patient reaching 6 months or withdrawing to the last patient reaching 15 years after surgery or withdrawing) annual summaries of safety, activity and survival will be generated.

Sample Size

This is an exploratory Phase I/II study and the primary objective is to evaluate safety. No formal sample size calculation has been performed. The sample size is typical of Phase I/II studies of a similar nature.

Safety Monitoring Committee (SMC) Review

Safety listings and tables will be provided as appropriate for review by the SMC in accordance with the SMC Charter. The SMC may make recommendations based on safety relating to the study including but not limited to recommendations relating to the dosing of future patients in Part A and Part B of the study, as detailed in the SMC Charter.

3 STUDY SCHEDULE

Table 1: Schedule of Evaluations

Procedure	Screening[p]		Post AXO-Lenti-PD Administration											Follow up					Annually for life
	Days		Days						Months										
	-60 to -1	-1	1 Surgery / ISP (Part B)	2	3	4	8	15	1	3	6	9	12	18 mths	2,3, **5** yrs[n]	4 yrs[n]	6-9, 11-14 yrs Phone	10, 15 yrs[n]	Phone
Visit window	*- 7 days*	*- 1 day*	*± 0 days*	*± 0 days*	*± 0 days*	*± 1 days*	*± 2 days*	*± 2 days*	*± 3 days*	*± 7 days*	*± 14 days*	*± 14 days*	*± 14 days*	*± 28 days*	*± 28 days*	*± 28 days*	*± 28 days*	*± 28 days*	
AXO-Lenti-PD administration[a]			X																
Hospitalisation[b]	X	X	X	X	X	X				X	X		X		X				
Entry Criteria	X	X																	
Signed consent[c]	X																		
Medical history[d]	X																		
PD medication / treatment	X	X	X	X	X	X	X	X	X	X	X	X	X	X	X	X	X	X	
L-DOPA review	X	X	X	X	X	X	X	X	X	X	X	X	X	X	X	X	X	X	
Concomitant medication	X	X	X	X	X	X	X	X	X	X	X	X	X	X	X	X	X	X	
Anaesthesia assessment[e]	X[e]	X																	
Vital signs[f]	X	X	X[f]	X	X	X	X	X	X	X	X	X	X	X	X	X			
ECG	X									X	X		X						

Table 1: Schedule of Evaluations

Procedure	Screening [p]		Post AXO-Lenti-PD Administration											Follow up					Annually for life
	Days		Days						Months										
	-60 to -1	-1	1 Surgery / ISP (Part B)	2	3	4	8	15	1	3	6	9	12	18 mths	2,3, **5** yrs[n]	4 yrs[n]	6-9, 11-14 yrs Phone	10, 15 yrs[n]	Phone
Chest X-ray	X																		
Physical examination	X	X[u]	X[u]	X	X	X	X	X	X	X	X	X	X	X	X	X			
Neurological examination[g]	X	X	X	X	X	X	X	X	X	X	X	X	X	X	X	X		X	
UPDRS Part 3 (in “OFF”)[h]	X									X	X		X		X				
UPDRS Parts 1-4 (in “ON”)[i]	X								X	X	X	X	X	X	X	X		X	
Optional video recording of patients for “OFF””/”ON” tests of UPDRS Part III and Dyskinesia Scale	X									X	X		X						
Hoehn & Yahr (in “OFF”)[h]	X									X	X		X		X				
Hoehn & Yahr (in “ON”)[i]	X								X	X	X	X	X	X	X	X		X	
Mattis Dementia Rating Scale-2™ (DRS-2)	X										X								
UPDRS Part 3 Area Under the Curve (AUC) analysis of Levodopa challenge (at selected sites)	X									X	X		X						
PDQ-39	X								X	X	X	X	X	X	X	X	X	X	

Table 1: Schedule of Evaluations

Procedure	Screening [p]		Post AXO-Lenti-PD Administration											Follow up					Annually for life
	Days		Days						Months										
	-60 to -1	-1	1 Surgery / ISP (Part B)	2	3	4	8	15	1	3	6	9	12	18 mths	2,3, **5** yrs[n]	4 yrs[n]	6-9, 11-14 yrs Phone	10, 15 yrs[n]	Phone
EQ-5D-5L	X								X	X	X	X	X	X	X	X	X	X	
Hauser Patient Diary (3-consecutive-day period)	X								X	X	X	X	X	X	X	X	X	X	
Non-Motor Symptom Scale (NMSS)	X								X	X	X	X	X	X	X	X	X	X	
Hauser Patient Diary (3-consecutive-day period)	X								X	X	X	X	X	X	X	X	X	X	
Hauser Patient Diary Training and Concordance Testing	X																		
C-SSRS "baseline" version	X																		
C-SSRS "since last visit" version									X	X	X	X	X	X	X	X	X	X	
Rush Dyskinesia Scale before L-DOPA medication withdrawn for "OFF"' assessments ("Prior to OFF")	X									X	X		X		X				
Rush Dyskinesia Rating Scale (in "OFF")[h]	X									X	X		X		X				

Table 1: Schedule of Evaluations

Procedure	Screening[p]		Post AXO-Lenti-PD Administration											Follow up					Annually for life
	Days		Days						Months										
	-60 to -1	-1	1 Surgery / ISP (Part B)	2	3	4	8	15	1	3	6	9	12	18 mths	2,3, **5** yrs[n]	4 yrs[n]	6-9, 11-14 yrs Phone	10, 15 yrs[n]	Phone
Rush Dyskinesia Rating Scale (in "ON")[i]	X								X	X	X	X	X	X	X	X		X	
Clinical Global Impression Scale	X								X	X	X	X	X	X	X	X		X	
Patient Global Impression Scale	X								X	X	X	X	X	X	X	X		X	
Cognition assessment (MoCA)	X								X	X	X	X	X	X	X	X		X	
Depression/mood assessment (HADS)	X								X	X	X	X	X	X	X	X		X	
Impulsivity assessment (QUIP-RS)	X								X	X	X	X	X	X	X	X		X	
Starkstein Apathy Scale	X								X	X	X	X	X	X	X	X		X	
Safety laboratory blood samples[j]	X	X			X		X		X	X	X	X	X						
Urinalysis[k]	X	X			X						X		X						
Blood for qPCR	X			X	X		X		X	X	X	X	X	X	X	X			
Blood for Immunology	X							X	X	X	X	X	X	X	X	X			
Urine for qPCR	X			X	X		X		X	X	X	X	X	X	X	X			

Table 1: Schedule of Evaluations

Procedure	Screening[p]		Post AXO-Lenti-PD Administration											Follow up					Annually for life
	Days		Days						Months										
	-60 to -1	-1	1 Surgery / ISP (Part B)	2	3	4	8	15	1	3	6	9	12	18 mths	2,3, **5** yrs[n]	4 yrs[n]	6-9, 11-14 yrs Phone	10, 15 yrs[n]	Phone
Optional blood sample (genomic)[l]	X																		
Brain MRI scan	X	X[o]	X[o] [q]	X[q]	X[q]				X		X		X						
CT scan at investigational site			X[o]																
PET/CT scan in "OFF" medication state[r] (PHNO)	X										X[v]		X[m, v]						
Optional PET/CT scan in "ON" medication state[r]	X										X		X						
AEs	X	X	X	X	X	X	X	X	X	X	X	X	X	X	X	X	X	X	X
Survival status														X	X	X	X	X	X
Urine pregnancy test[s]	X[t]	X								X	X[t]	X[t]	X[t]						

Notes:

a. AXO-Lenti-PD administration will be to all Part A patients, and those in Part B randomised to active study treatment
b. Patients will be hospitalised for at least 72 hours post-surgery, and for one night preceding each "OFF" assessment to enable all testing to be carried out.
c. Written informed consent is required before any protocol-specified procedures or assessments are performed
d. Medical history includes full PD history, prior PD treatments, and demographics; baseline PD symptoms also to be recorded
e. Optional additional anaesthetic assessment between days -28 to -2 per hospital practice
f. Vital signs: blood pressure (BP, lying down and standing up (no standing up immediately post-surgery)), heart rate (HR) and temperature; weight only at screening, Day -1, Day 15, and all subsequent visits up to the Year 5 follow up; height at screening only.

g. Neurological examination will include assessments of the patient's level of consciousness, speech, cranial nerves, muscle strength, coordination/cerebellar function, sensation and reflexes. Acceptable to limit assessments on post-surgery days 1, 2 and 3 according to patient's ability to be assessed.
h. Assessments in "OFF" defined as before the first morning dose of L-DOPA and at least 12 hours after the last administration of L-DOPA the previous day (see Section 8.4.9.2).
i. Assessments in "ON" defined as a minimum of one hour after a dose of L-DOPA (see Section 8.4.9.3).
j. Safety laboratory blood samples will comprise:
   - Haematology: white blood cell (WBC) count, red blood cell (RBC) count, haemoglobin (HGB), haematocrit (HCT), mean corpuscular volume (MCV), mean corpuscular haemoglobin (MCH), red cell distribution width (RDW), erythrocyte sedimentation rate (ESR), platelets, neutrophils, lymphocytes, monocytes, eosinophils
   - Chemistry panel: serum electrolytes (phosphorus, calcium, sodium, chloride and potassium), creatine phosphokinase (CPK), lactate dehydrogenase (LDH), albumin, creatinine, blood urea nitrogen (BUN), uric acid, alanine transaminase (ALT), aspartate transaminase (AST), total bilirubin (TBIL), alkaline phosphatase (AP), total protein (TP), gamma glutamyl transferase (GGT). Tests only at screening and M6: fasting blood glucose, glycosylated haemoglobin test (Haemoglobin a1c), total cholesterol (CHOL), low-density lipoprotein cholesterol (LDL), high-density lipoprotein cholesterol (HDL), triglycerides, thyroid stimulating hormone (TSH), C-reactive protein (CRP).
   - Coagulation: prothrombin time (PT), partial thromboplastin time (PTT)
k. Urinalysis: by dipstick, with microscopic urinalysis required only if dipstick/symptoms are indicative of need
l. Optional blood sample for PD-related research, including but not limited to genomic analysis. Genomic blood sample will be collected at any time during the screening period
m. Optional PET imaging assessment, in "OFF" medication state, at 12 months
n. Adverse events, PD medication, alternative PD treatment, concomitant medication, and L-DOPA assessments will be obtained via a phone call should the patient be unable to attend for hospital visits.
o. A pre-operative brain MRI scan can be performed the day before surgery followed on the day of surgery by a CT scan at sites where MRI scans cannot be performed on the day of surgery.
p. Screening assessments may be performed over more than one visit during the Screening period.
q. The post-operative MRI to assess surgical artefacts and confirm target localization should preferably be taken within 3 hours of surgery completion, but no longer than 24 hours after. Post-operative MRI images shall be transmitted to imaging storage vendor.
r. Only patients eligible for the main study and considered appropriate for the PET sub-study by the investigator will attend the PET centre for the conduct of the PET at the visits where PET is performed. Patients who are not able to tolerate PET-related procedures based on investigators' judgement will not be required to participate in the PET sub-study. The PET centre will be at a different location from the investigational site and the PET visit will occur on a different day from the clinical visit but within the visit window allowed.
s. Pregnancy test will be required only for women of childbearing potential.
t. For women of childbearing potential participating in the PET sub-study, urine pregnancy test will be required at the PET centre for each PET visit, and during Screening Days -60 to -1 two urine pregnancy tests will be performed, one at the clinical site and one at the PET centre. Pregnant women will not be scanned. During Screening Days -60 to -1, for women of childbearing potential not in the PET sub-study, one urine pregnancy test will be performed at the clinical site.
u. A full or focused physical exam must be performed on day -1 AND/OR day of surgery, but cannot be excluded on BOTH day -1 AND day of surgery.
v. An acquisition window of ± 30 days for PET scans is allowed for feasibility and patient access.

4 STUDY FLOW DIAGRAM

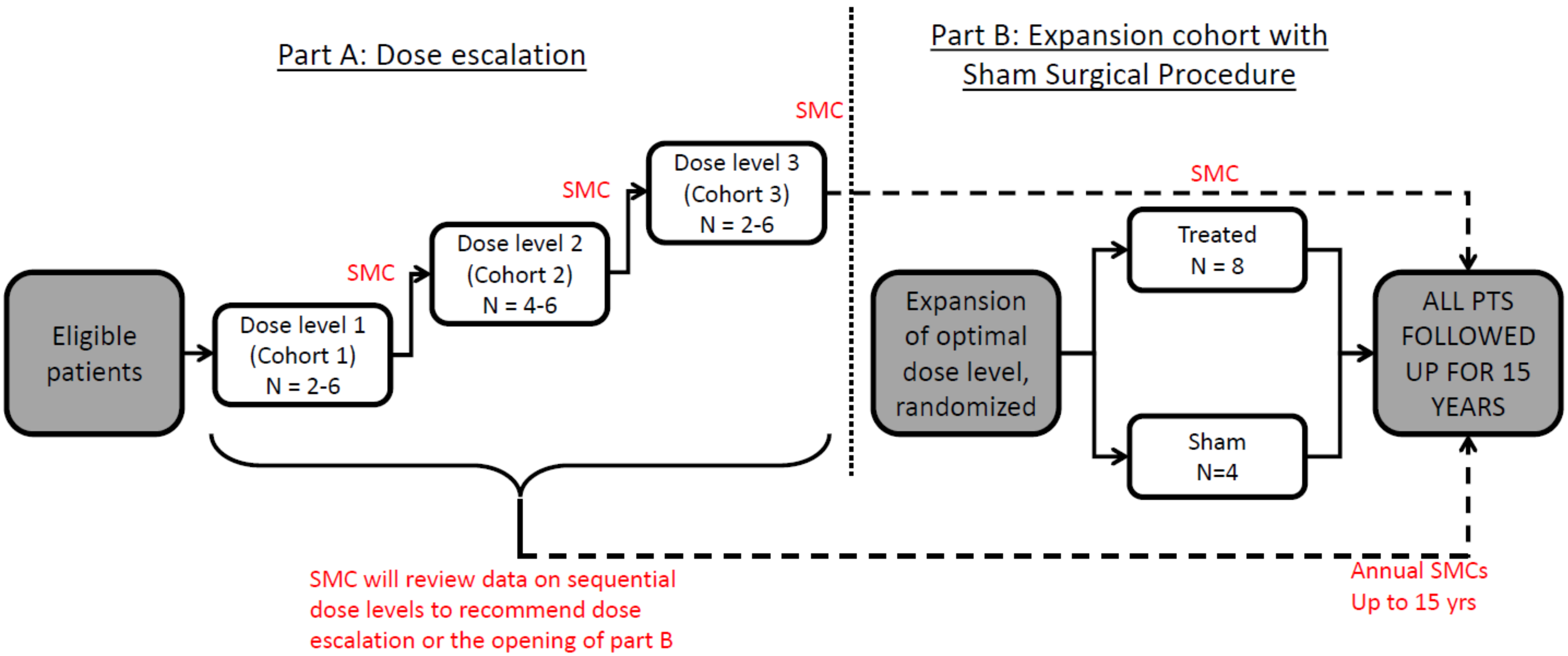

5 INTRODUCTION AND RATIONALE

5.1 Background to Parkinson's Disease

Parkinson's disease (PD) is a progressive, degenerative condition of the central nervous system (CNS) mainly affecting the motor system. The cause of PD for most patients is unknown, and this is referred to as 'sporadic' or 'idiopathic' PD. It is estimated that 6.3 million people worldwide have PD, and although most people will develop the symptoms after 60 years of age, approximately one in ten are diagnosed before the age of 50. Slightly more men than women are affected.[1]

In patients with PD, the most consistent and significant loss of function occurs in the dopamine-producing cells of the substantia nigra pars compacta in the basal ganglia. These neurons send axonal projections to the striatum where the neurotransmitter, dopamine, is released from the terminals. Within the basal ganglia there are two main efferent pathways from the striatum, the direct pathway and indirect pathway, each consisting of more than 80% medium-sized gamma-aminobutyric acid producing neurons. Dopamine excites the direct pathway (striatum, globus pallidus internus [GPi], substantia nigra pars reticulate and thalamus) and inhibits the indirect pathway (globus pallidus externa, subthalamic nucleus [STN] and thalamus) within the basal ganglia. Dopamine helps coordinate movement by acting on both pathways which counterbalance one another. The loss of dopaminergic input to the striatum in PD leads to an imbalance in the basal ganglia circuitry which results in severe motor impairment. The classic clinical symptomatology, characterised by a resting tremor, bradykinesia and muscle rigidity, typically manifests when the dopaminergic loss in the striatum is in excess of 60%. Many people with PD may experience other changes, known as non-motor symptoms, which can vary substantially from patient to patient. Examples of such symptoms include: change in taste and smell, bladder dysfunction, dementia and cognitive impairment, hallucinations, depression and anxiety, sexual dysfunction, orthostatic hypotension, excessive daytime sleepiness, insomnia, delusions and impulse control disorders.

There is no curative treatment or therapy to halt the disease progression of PD. Current treatment options include pharmacological and surgical approaches. The early stages of disease can be effectively managed by oral dopaminergic treatments, such as L-DOPA therapy, dopamine agonists and enzyme inhibitors that aim to stop the breakdown of L-DOPA or dopamine in the periphery. After approximately 5 years of oral dopaminergic treatments, 50% of patients develop motor problems, such as dyskinesias, in particular after prolonged and pulsatile use. The associated pill burden of oral dopaminergic treatments is substantial and increases as combination treatments are added in the more severe stages of the disease. Once the oral drugs start to fail in mid to late stage PD there is no standard care. At this stage more invasive surgical therapies to control motor functions and to reduce hypomobility episodes are introduced, including deep brain stimulation (DBS), Duodopa® (combined levodopa/carbidopa) and apomorphine (dopamine agonist) pumps. It is estimated that about

10 to 20% of patients with PD may be eligible for surgical treatment[2].These treatments are invasive and expensive, both in terms of acquisition costs and ongoing care, and have clinically significant contraindications. Deep brain stimulation involves stereotactic implantation of electrodes into a selective area of the brain such as the thalamus, STN or GPi. The electrodes are attached to a pulse generator that is placed within the patient's chest. Although DBS is contraindicated in patients with dementia; which is twice as prevalent amongst PD patients as the normal population, since 1997, more than 135 000 PD patients worldwide have received Medtronic DBS[3] (data from Medtronic website). Not all patients respond to DBS and adverse effects on cognitive function are an area of specific concern. Nevertheless, DBS now sees an annual accrual of 8000 to 10 000 new patients per year worldwide[4] and the cost per quality adjusted life-year is positive compared to the best medical care for appropriate patients[5].

Gene therapy offers an innovative approach for the treatment of mid to late stage PD. The striatum is well-defined and surgically accessible making gene targeting of cells a feasible approach with minimal disruption of other brain regions. Gene-based therapeutic strategies include the stimulation of dopamine production in the striatum, to depress over excitation in the STN or to allow the production of growth factors to retard or repair the degeneration, and/or the increase in activity of dopaminergic neurons that project into the striatum[6, 7, 8, 9, 10]. Lentiviral vectors are promising gene therapy delivery systems that are capable of transducing non-dividing cells, such as neurons, and maintaining long term expression[11, 12]. Oxford BioMedica plc (OXB, United Kingdom) has developed a first-generation gene therapy product called ProSavin®, which has been used in a phase I/II study in patients with bilateral idiopathic PD, (PS1/001/07, EudraCT 2007-001109-26), and continues to be assessed in a long term follow up study, (PS1/001/09, EudraCT 2009-017253-35). Data from these studies demonstrated ProSavin® was safe and well tolerated and to have encouraging signs of biological effect[13]. AXO-Lenti-PD has been developed as an enhanced version of the ProSavin® vector, expressing the same three enzymes as ProSavin® but with at least five times increased dopamine production per genetically modified cell[14.]

5.2 Background to AXO-Lenti-PD

AXO-Lenti-PD is a second-generation gene therapy medicinal product developed initially by OXB and now by Axovant Sciences for the treatment of levodopa-responsive PD. The therapeutic rationale for the treatment of PD is to provide dopamine replacement to the dopamine-depleted putamen of PD patients by gene transfer of the three critical enzymes in the dopamine biosynthesis pathway.

The AXO-Lenti-PD vector is a second-generation product to an earlier construct, ProSavin®. Both products encode three enzymes in the dopamine biosynthetic pathway, the truncated form of human tyrosine hydroxylase (TH), human GTP-cyclohydrolase 1 (CH1), and human aromatic L-amino-acid decarboxylase (AADC). The genes are delivered to the target cell populations by local bilateral stereotactic infusion of AXO-Lenti-PD into the sensorimotor

putamen of PD patients. This results in expression of the corresponding proteins in non-dopaminergic neurons thus converting these cells to dopamine ‘factories’ resulting in replacement of the dopamine that is depleted in PD. The maintenance of a constant dopaminergic tonic level within the putamen offers the potential to reduce the level of L-DOPA and dopamine agonist therapy and provide a sustained motor correction with longer "‘ON" periods and shorter “OFF” periods.

The completed phase I/II study with ProSavin® was an open label dose escalation study (PS1/001/07, EudraCT 2007-001109-26), and patients from this study were invited to enrol into a long-term follow on study, (PS1/001/09, EudraCT 2009-017253-35), designed to monitor the safety and tolerability of ProSavin® for up to 10 years post treatment. These studies, which were conducted in two sites in two countries, (France and the UK) evaluated three dose levels of ProSavin® and demonstrated it to be safe and well tolerated and to have encouraging signs of biological activity although a consistent dose response was not observed[13]. As the data indicated that the optimal therapeutic dosing regimen for ProSavin® had not been achieved, further exploration into increasing the potency was warranted. However, as neither increasing the manufactured dose nor the volume delivered per needle track were feasible, OXB developed an enhanced version of the ProSavin® vector, AXO-Lenti-PD, expressing the same three enzymes as ProSavin® but with at least five times increased dopamine production per genetically modified cell. Data from *in vitro* transduced primary human neuronal cultures and from the N-methyl-4-phenyl-1,2,3,4-tetrahydropyridine (MPTP) macaque model of PD demonstrate that AXO-Lenti-PD is more efficacious than ProSavin®.

Although AXO-Lenti-PD is a new Investigational Medicinal Product (IMP), it differs from ProSavin® only in the configuration of its genetic cargo. Given that the lentiviral vector delivery system used in ProSavin® and indeed the therapeutic genes encoded within the products are identical, it is anticipated that clinical and nonclinical data already generated with ProSavin® will provide important supporting information on the safety and tolerability of AXO-Lenti-PD. Accordingly, in order to enable comparison across the PD clinical programme completed to date, the study will be conducted in a very similar patient population (the upper age limit is increased from 65 years to 70 years) and will include the same sites which participated in the ProSavin® clinical study.

Summaries of the nonclinical data generated to date are provided in the Investigator Brochure (IB) for the IMP accompanying this protocol. The IB also contains a summary of pertinent non-clinical and clinical data generated on the related product ProSavin®.

5.3 Description of Product

5.3.1 Introduction

AXO-Lenti-PD is a non-replicating non-primate, recombinant lentiviral vector based on the non-pathogenic wild type equine infectious anaemia virus (EIAV). The wild-type EIAV genome has 6 distinct genetic units, however, the majority of these EIAV sequences (~90%) have been

removed to produce a replication defective minimal vector system that contains less than 10% of the original viral genome and does not contain any of the natural viral promoters or enhancers and there are no coding regions for accessory proteins in either the EIAV genome or in the packaging system.

5.3.2 Description

AXO-Lenti-PD is formulated as a liquid product for putamenal infusion and is provided in 0.3 mL type I borosilicate glass 'V' vials with stopper and aluminium crimp seal. Each vial is filled with 130 µL of AXO-Lenti-PD. The product is presented as a frozen liquid formulation, stored at -80˚C. AXO-Lenti-PD is presented as a clear, colourless or white to off-white turbid solution with no visible particulates.

Two dose strengths (concentration in TU/mL) of AXO-Lenti-PD have been prepared for the proposed clinical trial (see Table 2). Alternatively, the dose strengths can be prepared by dilution of a more concentrated vector solution using tris, sucrose, sodium chloride, mannitol (TSSM) formulation buffer.

5.3.3 Composition of the Medicinal Product

The composition of AXO-Lenti-PD medicinal product at all dose strengths is presented in Table 2.

Table 2 Composition of AXO-Lenti-PD Drug Products (all strengths)

Name of Ingredients	Quantity	Function	Reference to Standards
**Active Substance:**<br>AXO-Lenti-PD	**Dose strength (titer)**: planned target titer was 7.0 x 10^6 TU/mL (≥2.2 x 10^6 to <2.2 x 10^7 TU/mL). Actual measured titer is 4.4 x 10^6 TU/mL<br><br>**Dose strength (titer)**: planned target titer was 2.3 x 10^7 TU/mL (≥7.3 x 10^6 to <7.3 x 10^7 TU/mL). Actual measured titer is 1.5 x 10^7 TU/mL	Active	N/A
**Excipients:**<br>Mannitol<br>Tromethamine (TRIS)<br>Sucrose<br>Sodium chloride<br>Hydrochloric acid<br>Water for Injection (WFI)	 1.0% w/v<br>20 mM<br>1.0%<br>100 mM<br>qs to pH 7.3<br>qs to volume	 Cryopreservation aid<br>pH Buffer<br>Cryopreservation aid<br>Tonicity agent<br>pH adjustment<br>Solvent	 Ph Eur/USP<br>Ph Eur/USP<br>Ph Eur/USP<br>Ph Eur/USP<br>Ph Eur/BP/JP<br>Ph Eur

BP=British Pharmacopoeia; JP=Japanese Pharmacopoeia; Ph Eur=European Pharmacopoeia; qs=quantum sufficit; TU=transducing units; USP=United States Pharmacopoeia

5.4 Summary of Pre-Clinical Studies

5.4.1 Summary of Nonclinical Studies

The development of AXO-Lenti-PD is one of a number of therapeutic products based upon the OXB lentiviral vector gene therapy platform.

Overviews of the nonclinical studies pertinent to the development of AXO-Lenti-PD that evaluate pharmacology, pharmacokinetics, toxicology and carcinogenicity are provided in the IB. The nonclinical development of AXO-Lenti-PD, using *in vitro* systems and in *vivo* studies in rats and macaques, builds on the platform of nonclinical information already compiled using ProSavin®. The dosing regimen for AXO-Lenti-PD was established empirically in rodents and non-human primates (NHPs) based on consideration of the relative size of the cynomolgus monkey and human brain and the historical experience of introducing suspensions into the brain gained during the development of ProSavin®.

Some key findings from the nonclinical development program with AXO-Lenti-PD are summarised as follows:

- In the NHP MPTP model of PD, high dose AXO-Lenti-PD animals (referred to as "full strength" in the study report) treated with vector doses of 1.52 x 10^7 or 2.18 x 10^7 TU, and low dose AXO-Lenti-PD animals (referred to as "1/5th strength") treated with vector doses of 3.04 or 4.36 x 10^6 TU were as efficacious as animals treated with a high dose of 1.22 x 10^7 TU ProSavin®. There was a marked temporal change in the 6-[^{18}F]Fluoro-L-*m*-tyrosine (^{18}FFMT) radiotracer signal that delineates the activity of AADC in the putamen in AXO-Lenti-PD treatment groups which was greater than in the ProSavin® treatment group. These data suggest that AXO-Lenti-PD is more potent than ProSavin®, shown in study PSC0148.
- Combined L-DOPA and dopamine concentrations in primary human cortical neuronal cells transduced with the AXO-Lenti-PD were approximately 10-fold higher compared to ProSavin®, see study CEG001 and CEG003
- No product-related time limit for efficacy has been reached in any of the AXO-Lenti-PD nonclinical studies (the latest time point was 6 months), see study PCS0148.
- Biodistribution and vector persistence of AXO-Lenti-PD in non-target tissues and the potential for vector shedding (tested to 6 months) following a clinically relevant route of administration are limited, and vector was not detected in non-target tissues,, see study AP129 supplemental to PCS0171/ HBW0003-00.
- Safety has been evaluated in toxicology study in NHPs. There have been no AXO-Lenti-PD-related toxicities, see PCS0171.

- Long-term AXO-Lenti-PD integration had no adverse effects on Human Embryonic Kidney 293 Large T antigen (HEK293T) cells *in vitro*, CEG003
- The potential for AXO-Lenti-PD to induce an immune response has been evaluated by monitoring serum for antibodies to the AXO-Lenti-PD vector and to the therapeutic gene products by Western blot analysis. Antibodies to vector components have been detected in the serum of animals but there was no response against the transgene products, AP130 (suppl. To PCS0171/ HWB0003-00)
- Interim results from study LNT-PD-1001 in NHPs provide safety and tolerability data for an increased flow rate and dose volume that will be used to support changes to the dose regimen in Cohort 3

Overview of key findings from ProSavin® development that are also pertinent to AXO-Lenti-PD:

- Efficacy has currently been sustained for up to 44 months in the NHP (the last time point in the study) following a single treatment of ProSavin®, see ProSavin2 (NHP2).
- Safety has been evaluated in toxicology studies in naïve rats and macaques. There have been no ProSavin® related toxicities, see PCS0021/HAG5000, PCS0026/20060333TCYB.
- No adverse events (AEs) were observed when ProSavin® was co-administered with L-DOPA or the dopamine agonist apomorphine in the NHP, see NHP1, NHP2, NHP3.
- The potential for ProSavin® to induce or exacerbate dyskinesia has been studied using quantitative dyskinesia scoring in the NHP PD model and in preliminary studies using dyskinetic animals. No induction or exacerbation of pre-existing dyskinesias have been observed in any of these studies, see NHP6.
- The potential for dopamine toxicity has been evaluated by measuring dopamine levels in ProSavin® treated animals and these have been shown in all cases to be much less than normal physiological levels, see NHP1, NHP2.
- The distribution of integrated vector genomes at the target site and non-target sites has been examined using sensitive PCR methods and the impact of integration has been considered by analysing more than 500 integration sites in cell lines, primary rat astrocytes and rat brain. No bias in the integration sites associated with increased risk of activating an oncogene has been observed, see ASTROR001, AP034.
- In rats there is no marked shedding of ProSavin® from urine, blood or the surgical site at 7 days post administration, see HAG5006.

5.4.2 Nonclinical Pharmacology

5.4.2.1 Evaluation of Efficacy

A number of studies have been undertaken using *in vitro* systems and *in vivo* models to evaluate the pharmacology of AXO-Lenti-PD, the details of which are provided in the IB.

The AXO-Lenti-PD *in vitro* studies were designed to identify an enhanced vector with improved production of dopamine in comparison to ProSavin®. The *in vitro* studies evaluated the detection of dopamine-synthesising enzymes and absolute catecholamine levels in transduced cells. Following completion of study CEG003, the modified vector TCiAmod was identified as the configuration with a much improved dopamine production profile compared to ProSavin®. The vector was designated as OXB-102 and later as AXO-Lenti-PD.

The *in vivo* studies were designed to further assess the behavioural efficacy of AXO-Lenti-PD. Two well established *in vivo* animal models of PD, the 6-OHDA lesioned rat (PCS0146/BRIS016) and the MPTP lesioned cynomolgus macaque (PCS0148), were used to assess this efficacy. In a 6-OHDA lesioned rat model study (PCS0146/BRIS016), there was no statistically significant differences in either amphetamine- or apomorphine-induced rotational behaviour or striatal catecholamine levels between control vector (LacZ) and ProSavin or AXO-Lenti-PD treated animals, likely due to small group sizes. However, in the MPTP macaque model (PCS0148) there was a statistically significant improvement in the clinical rating scores in animals injected with two doses of AXO-Lenti-PD, and increased movement compared to the control treated animals. In particular, ^{18}F-FMT based PET scans indicated the AADC activity in AXO-Lenti-PD treated animals was greater than in the ProSavin®treated animals (dose 1.22×10^7). The two doses of AXO-Lenti-PD evaluated in this study were a high dose in the range of 1.52 to 2.18×10^7 and a low dose in the range of 3.04 to 4.36×10^6.

5.4.3 Nonclinical Pharmacokinetics

A series of pharmacokinetic studies have been performed *in vivo* and *in vitro* to assess the biodistribution and dissemination of vector-associated nucleic acids. Further details of each of these studies can be found in the IB.

Analysis of the biodistribution of AXO-Lenti-PD in NHPs following a single 100 µL infusion into each putamen in the toxicology study PCS-171/HBW0003-00/AP129 showed that there were no vector sequences detected in off-target tissues (gonads, spleen, liver, lung) at 6 months. The results indicate that there is no significant dissemination of the vector beyond the brain following targeted delivery of the vector into the putamen region in cynomolgus macaques. These results were consistent with the previously generated ProSavin® data.

Vector shedding in NHPs was analysed using blood (plasma and buffy coat) and cerebrospinal fluid (CSF) from two ProSavin® studies (ProSavin® Report Number 20060107SPG and toxicology ProSavin® Report Number 20080394TCYPB/AP085) and from the AXO-Lenti-PD toxicology study (Report Number AP129 sub-report to PCS0171/HBW0003). qPCR and RT-qPCR data from these studies indicated that EIAV Ψ ribonucleic acid (RNA)/

deoxyribonucleic acid (DNA) was below the limits of quantification (ProSavin® Report Numbers AP057 /AP048, AP085; AXO-Lenti-PD Report Number AP129). Vector RNA was not detected in the CSF when tested at 3 or at 9 months post administration for ProSavin® and at 6 months post-administration for AXO-Lenti-PD (Report AP129).

Results from these studies have demonstrated that the distribution and persistence of AXO-Lenti-PD and ProSavin® to non-target tissues and the potential for vector shedding following a clinically relevant route of administration is limited and that vector is not detected in non-target tissues beyond 1 month (AP129, sub-report to PCS0171/HBW0003).

5.4.4 Nonclinical Toxicology

AXO-Lenti-PD has been tested in Good Laboratory Practice (GLP) nonclinical safety study in 12 cynomolgus macaques (PCS0171/HBW0003-00), in which a total volume of 200 µL of AXO-Lenti-PD (100 µL/hemisphere) was administered bilaterally into the striatum using the maximum feasible dose (1.3×10^7 TU)at an infusion rate of 3 µL/min. The titre of the AXO-Lenti-PD vector used in this study was 6.5×10^7 TU/mL and each animal in the AXO-Lenti-PD treatment group received a total dose of 1.3×10^7 TU (total volume of 200 µL/animal). The PD human brain volume is 21-fold greater that the cynomolgus macaque brain. Therefore, the total dose delivered to the macaque brain in the toxicology study is allometrically equivalent to a 2.73×10^8 TU dose ($1.3 \times 10^7 \times 21$) in the PD human brain.

Possible toxicological effects may occur over a short time period due to the effects of the vector particles (specific properties and process related impurities) and the most likely event would be acute inflammation. Effects occurring over longer periods of time may be due to the integrated vector and could involve a chronic immune response to the vector components, effects of vector integration or effects of the transgene product, dopamine. A chronic Good Laboratory Practice nonclinical toxicology study with AXO-Lenti-PD was undertaken in one species (cynomolgus macaque) and included a sacrifice time point of 6 months. There were no AXO-Lenti-PD-related toxicities observed. All study findings were attributed to background findings of animals of that age or due to the surgical procedure. This study demonstrated that AXO-Lenti-PD is safe and well tolerated in NHPs when delivered by bilateral administration into the brain.

Immunological analysis showed that antibody responses against the Vesicular Stomatitis Virus Glycoprotein 2 (VSV-G2) envelope protein were generated in all 12 animals in the NHP AXO-Lenti-PD toxicology study (Report Number AP130 Sub-report to PCS0171/HBW0003-00); of the anti-VSV-G2 responses were initially detectable at 4 weeks post-administration of AXO-Lenti-PD, and remained present at the 6 month time point. Responses against EIAV p26 capsid protein were also generated in 4 animals, while a response against a HEK293T packing cell component was detected in a single animal. However, there were no detectable antibody responses to any AXO-Lenti-PD transgene products or neomycin phosphotransferase at any time point.

5.4.4.1 Nonclinical Toxicology Supporting Cohort 3

A GLP nonclinical safety study (LNT-PD-1001) was conducted to assess the safety and tolerability of increased dose volumes and flow rate of infusion of the AXO-Lenti-PD drug product to support the dosing plan for Cohort 3; see also Section 5.6.4. The study used 14 cynomolgus macaques (most relevant species) to dose a total volume of 500 μL of either EIAV-LacZ or AXO-Lenti-PD (250 μL/hemisphere) which was administered bilaterally-simultaneously into the striatum at an infusion rate of 5 μL/min. The titer of AXO-Lenti-PD vector used in this study was 1.6×10^7 TU/mL and each animal in the AXO-Lenti-PD treatment group received a total dose of 8.0×10^6 TU. Animals were assessed at 1 month post dosing for acute toxicological findings and at 3 months for chronic findings. Interim toxicology results are available for both Day 28 EIAV-LacZ and AXO-Lenti-PD and biodistribution Day 28 data is available for EIAV-LacZ.

AXO-Lenti-PD and EIAV-LacZ were not associated with unscheduled deaths. Antemortem endpoints (i.e., clinical observations, body weight, ophthalmoscopic, functional behavioural, and clinical pathology examinations) were unaffected by dosing of the test article (EIAV-LacZ or AXO-Lenti-PD). There were no test article-related organ weight changes, macroscopic or microscopic findings in non-nervous system tissues at the Day 28 $\pm$2 necropsy. Microscopic findings of minimal to slight perivascular mononuclear cell infiltration with/without pigmented macrophage infiltration were reported but these were limited to the injection sites (putamen and surroundings) and were without any associated systemic observations. Biodistribution was assessed for the EIAV-LacZ at Day 28 $\pm$2, showed the test article was localized to the brain, specifically the target site of administration, with no vector detected systemically.

5.4.5 Carcinogenicity

A series of *in vitro* studies and a small *in vivo* study were previously conducted to determine the integration site profile of the EIAV-based lentiviral vector system to support the ongoing development of related lentiviral vector product, ProSavin® (see ASTROR001, AP034 sub-report DPJK1001, CEG003). Given the common vector structural components used for the EIAV lentiviral vector platform, the body of integration site data is considered to be directly relevant to inform the integration site profile of the vector system platform as a whole, including AXO-Lenti-PD.

Further, whilst AXO-Lenti-PD is a lentiviral vector which integrates its genetic cargo into the genome of the target putamen cell, it is replication incompetent and has a low risk associated with insertional mutagenesis. The target cells are differentiated cells (i.e. neurons) and hence are post-mitotic so, by definition, genes involved in cell division will not be active in these cells.

It is believed that treatment with AXO-Lenti-PD offers very little opportunity to contribute to oncogenesis because the EIAV vector system is minimal such that it has been engineered to remove all viral genes and promoter sequences to minimise the potential to transactivate genes proximal to its insertional site. During the production process, envelope components

and other structural proteins required for vector packaging and transduction are supplied *in trans* and encoded on separate plasmids. Moreover, the complete vector system is split across three plasmids in total to minimize the risk of generating a replication competent lentivirus (RCL) and assays that were developed and validated demonstrate that RCL have not been found. Two Benzonase® treatment steps (degrades DNA) are included in the manufacturing process to prevent the delivery of any intact genes as impurities to the target cells.

Tumorigenic AEs following retroviral gene therapy have been observed in three clinical settings: in a trial for chronic granulomatous disease (CGD) 3 out of 12 patients developed myelodysplasia with one death[15], in a trial for Wiskott-Aldrich syndrome 7 out of 10 patients developed leukaemia and is undergoing treatment[16] and in X-linked severe combined immunodeficiency (X-SCID) trials 5 out of 20 patients have developed leukaemia with four patients successfully treated and one death. In the X-SCID trials the tumorigenic events have been attributed to insertional mutagenesis[17,18]. Both the X-SCID, the CGD and the Wiskott-Aldrich syndrome trials utilized gammaretroviral vectors which are different to the lentiviral vector used for AXO-Lenti-PD. The gene therapy community has concluded that it is likely that other mutations contributed to these events but the proliferative advantage supplied by the vector was necessary to allow these secondary mutations to develop and be selected. The AEs in these trials appear to be protocol, vector and transgene specific; the therapeutic gene favoured proliferation, aspects of the protocol either required or allowed selective advantage of the transduced cells and the recipients were immuno-compromised. The following features markedly mitigate the tumourigenic risks of the AXO-Lenti-PD protocol, when compared to the X-SCID protocols, thus removing some of the key features that are known to have contributed and/or been causative for the observed AEs reported:

- The target cells are differentiated cells (i.e. neurons) and hence are post-mitotic so genes involved in cell division will not be active in these cells.
- Dopamine synthesising genes are not central to cell survival or replication and so are unlikely to confer a proliferative advantage to target cells.
- In haematopoietic transplantation models in normal and tumour-prone mice using lentiviral vectors, no increase in the incidence of leukaemia was reported.
- Patients in the OXB-102-01 clinical study are not immunocompromised, and are not treated with immunosuppressive drugs.
- Unlike gammaretroviral vectors, EIAV vectors do not integrate preferentially into the promoter region or the 5’ end of the transcription unit, and are therefore less likely to up regulate gene expression.
- In an attempt to assess the tumourigenesis potential of EIAV vectors, an *in vitro* soft agar assay using WI38 cells (foetal human lung fibroblasts) was used to evaluate EIAV vectors at high copy number; there was no evidence for tumourigenesis in these studies.

In addition, AXO-Lenti-PD nonclinical studies have demonstrated that AXO-Lenti-PD is safe, well tolerated and efficacious. AXO-Lenti-PD is carefully delivered to a discrete region of the brain, and biodistribution and shedding studies indicate AXO-Lenti-PD is mostly confined to the brain. The majority of cells transduced are neuronal in nature. It is also unlikely that production of dopamine will be problematic following AXO-Lenti-PD administration. There has not been any suggestion of carcinogenicity or chronic histopathology in a 6-month NHP study.

In conclusion, the potential for AXO-Lenti-PD to cause any carcinogenic risk is considered to be low.

5.4.6 Effects in Humans

The clinical development of AXO-Lenti-PD builds on the platform of clinical information already compiled using ProSavin®. Cumulative AE data reported from the baseline of PS1/001/07 to the data cut-off date of 11th December 2018 from Study PS1/001/09 have been analysed and validated. This provides safety data on the administration of ProSavin® from 15 patients for a minimum of 36 months, a minimum of 7 years for the 10 ongoing patients and up to 10 years of exposure for the earliest treated patients.

5.4.6.1 Safety and Efficacy

The results of the ProSavin® Phase I/II study[13] are summarised here as a guide.

5.4.6.1.1 ProSavin® Safety

Serious Adverse Events

There has been one suspected unexpected serious adverse reaction (SUSAR) of worsening dyskinesias reported 7 years post-surgery over the clinical development programme with ProSavin®. As of 11th December 2018, 38 SAEs have been reported: one occurred during Study PS1/001/07 and 37 SAEs during the long-term follow-up study PS1/001/09. Three patients had an SAE with an outcome of death (peritonitis, cardio-respiratory arrest and Parkinson's disease) but all were not considered to have a reasonable possibility of a causal relationship to the investigational product or investigational procedures by the reporting Investigators and/or OXB. The greatest numbers of SAEs (15 of the 38) have been reported in the nervous system disorders System Organ Class (SOC). All SAEs were assessed as not related or unlikely to be related to ProSavin® or study procedures.

Adverse Events

From the cumulative AE data from studies PS1/001/07 and PS1/001/09 generated to 11th December 2017, all patients (100%) had a least one post-surgery AE. Overall, 730 treatment emergent AEs have been reported with 571 classed as mild, 128 classed as moderate and 30 classed as severe, and one was missing/unknown so could not be classified. Out of all treatment emergent events, 96 AEs were considered to be treatment

related (related to either IMP or the surgical procedure) although it should be noted 57 out of those 96 events were considered unlikely to be related. The most common AEs considered treatment related were reported in the nervous system disorders SOC. Of these, the most frequent were dyskinesia and “ON” and “OFF” phenomena, which abated when the dose of oral dopaminergic therapy was adjusted. All 15 patients (100%) experienced at least one related AE.

Immunotoxicity

There is no evidence of any local immunological or inflammatory reaction to ProSavin®. During Study PS1/001/07, antibody responses were not detected against any ProSavin® component in patients in Cohort 1 up to 24 months and Cohorts 2a or 2b up to 12 months following ProSavin® administration. Antibody responses against VSV-G were detected in at least one post-treatment serum sample from 4 out of the 6 patients in Cohort 3 (total vector dose $10x10^7$ TU). In 3 of the same patients, an antibody response against p26 (EIAV structural protein) was also observed. All patients were asymptomatic at the time. Anti-VSV-G (the envelope protein of the vector) responses appear to be decreasing 3-6 months after the response was first detected. Anti-p26 responses were detected at 6 months and were relatively weak as compared to the VSV-G responses.

Biodistribution/Vector Resistance/Vector Shedding

Analysis of blood and urine has not detected any consistent RNA or DNA from ProSavin®. ProSavin® DNA and RNA sequences were not detected in the majority of blood and urine samples. Where sequences were detected, they were present at a level that was below the lower limit of quantification with the exception of white buffy coat samples for 4 patients and for one plasma sample for 1 patient. In all cases, the sequences disappeared spontaneously and appeared to be of no clinical significance.

5.4.6.1.2 ProSavin® Efficacy

No efficacy claim was made for the doses of ProSavin® studied in PS1/001/07. Although there were some encouraging converging and consistent indices suggesting a potential dopaminergic response, the DMC concluded that the signs of clinical and biological activity even at the highest dose level were not superior to the published placebo effect associated with PD studies and were therefore not strong enough to warrant further investigation with ProSavin® other than the long term followup- of all patients treated to date. The DMC recommended the future clinical development of the more potent construct AXO-Lenti-PD. After further review of the longer term data from the PS1/001/09 study (a minimum of three years data on all patients and up to nearly seven years on some patients) the DMC acknowledged encouraging signs of biological activity but their recommendation to explore higher doses did not change.

5.5 Rationale for Study Conduct

As PD progresses there is increased neuronal loss within the substantia nigra leading to a reduction in the amount of dopamine released into the striatum. There is typically at least 60% loss of dopaminergic cells by the time patients present with clinical symptoms of PD[19]. However, such levels are considerably less when L-DOPA therapy starts to fail, with post mortem analysis detecting up to a 90% depletion[20, 21]. The provision of exogenous L-DOPA boosts dopamine levels from the remaining nigral neurons but as the disease progresses there are less neurons and these neurons become less efficient at metabolising the L-DOPA into dopamine and eventually it becomes impossible to provide sufficient L-DOPA without incurring side effects of severe debilitating motor dysfunction. The pulsatile dopamine release and receptor stimulation associated with L-DOPA administration are also thought to contribute to disturbances in motor function. This treatment regimen often results in the side effects of the treatment becoming as disabling as the disease.

It is thought that in part the motor complications and failing clinical responses observed with L-DOPA therapy are associated with its pulsatile administration, although the mechanisms are poorly understood[22]. Therefore, the rationale for AXO-Lenti-PD is to provide a continuous supply of dopamine to the striatum that is sufficient to enable restoration of normal movement in the absence of disabling side effects. It is envisaged that AXO-Lenti-PD will contribute to a continual supply of dopamine within the striatum and sustain stimulation of post-synaptic dopamine receptors. Such a constant production of dopamine may be sufficient to improve motor function and permit a decrease or removal of L-DOPA from the patient's treatment regimen. This decrease in drug therapy could potentially reduce the side effects associated with the pulsatile delivery of exogenous L-DOPA, and have a significant impact on the management of PD.

5.6 Rationale for Study Design, Doses, and Control Groups

5.6.1 Rationale for Study Design

This first in man study has been split into two parts:

Part A: open label, dose escalation design to determine safety and tolerability of AXO-Lenti-PD, and establish the optimal dose from this study in the study patient population.

Part B: double-blind design, with the same clinical assessments as Part A, with patients randomised to an active group (optimal dose of AXO-Lenti-PD found in Part A) or a control group (undertaking an imitation surgical procedure (ISP)) in a 2:1 ratio.

The rationale for including ISP is to assess the placebo effect from expectation of benefit (from patients and site staff), which would confound the data, and for which the physiological basis, magnitude and duration are unknown. It has been suggested that ISP-controlled studies are especially valuable when objectives and endpoints are based on subjective evaluations such as symptom improvements and quality of life[23]. Shetty and colleagues have reviewed a large

number of medical clinical trials for PD and found evidence of placebo effects in a large proportion of the studies[24]. In an analysis of the placebo effects during a drug trial for PD, Goetz and colleagues documented significant and persistent placebo effects over a six month period[25]. Placebo effects were evident with the most objective component of the evaluation, the motor score (Part III) of the UPDRS. Unlike most placebo effects, there may be a physiological explanation for them in PD. One group showed that placebo administration[26] evoked a substantial increase in dopamine release. In addition, the effect on dopamine receptor occupancy was similar to parenteral administration of the dopamine agonist, apomorphine or oral L-DOPA. One of the functions of the nigrostriatal dopamine projection may be to signal expectation for reward, such as the expectation of therapeutic benefit. This result is consistent with the present understanding of nigrostriatal neuronal firing. The results obtained by De la Fuente-Fernandez and colleagues are not however, a general explanation for placebo effects but are probably specific to nigrostriatal dopaminergic function.

There are however ethical concerns regarding the inclusion of ISP in clinical trials, as an ISP does carry more risk to a patient than a placebo pill. It has been suggested that three criteria be met before considering the conduct of a randomised double blind, placebo controlled trial; 1) the clinical trial should address an important question that cannot be answered by a study with an alternative design, which poses a lower risk to patients; 2) there must be preliminary but not conclusive evidence that the intervention is effective and 3) the treatment should be developed to a point where it is unlikely to become obsolete before the study has been completed[27]. Based on these three considerations, two neural transplant studies have compared active treatment with ISP[28,29] and also two phase II gene therapy studies included ISP[30,31]. The results of the two neural transplant studies indicated a variable clinical outcome of the study with a significant improvement in Schwab and England activities of daily living (ADL) scores in patients who received neural grafts compared to those who underwent ISP one year after surgery[32]. Reference was made to the fact there were no significant differences between patient diary score and doses of drugs in the two groups. The majority (75%) of the same patient population from the original study consented to participate in a related quality of life study[33]. The study investigated the quality of life of the patients relative to their perceived and actual treatment. The study suggested that the perception a patient has regarding their treatment may influence their behaviour thereby supporting the need for placebo controlled studies.

5.6.2 Rationale for Study Population and Disease Stage

The patient population for this clinical study will be very similar to that for the ProSavin® clinical study, the only key changes being an increase on the upper age limit from 65 years to 70 years and the UPDRS score for entry into the study has been narrowed from 20-60 to 30-60 to further standardise the advanced patient population. This patient population was selected, as it is one for whom current treatments are failing but whose disease has not progressed to extremes whereby there are extensive L-DOPA induced dyskinesias or where there is cognitive impairment. This group of patients could potentially receive some benefit from AXO-

Lenti-PD treatment. The patients must still be L-DOPA responsive, and assessed as being stage 3 to 4 on the Hoehn and Yahr scale in the “OFF” medication state.

Up to 30 patients of either sex between the ages of 30 and 70 years with bilateral idiopathic PD, in whom increased L-DOPA administration is demonstrating no additional clinical improvement in PD symptom management, will be included in the study. It is considered that data obtained in this selected group of patients will be relevant for a broader group of patients who could benefit from treatment with AXO-Lenti-PD at an earlier stage in the disease and who could be the subject of future clinical studies.

This population excludes patients who may be successfully managed for a number of years with conventional oral medications, as interventional brain surgery with an experimental agent not previously tested in humans would not be justified. Also excluded are patients with significant L-DOPA induced dyskinesia, as although data from the ProSavin® clinical trials demonstrated that dyskinesia was not exacerbated in the “OFF” medication state, it would similarly not be justified to include them at this stage of development.

It is intended that patients recruited into this study will share similarities with a subset of the population eligible for DBS. AXO-Lenti-PD patients eligible for this study will have increasing periods of “OFF” time despite optimal oral therapy but will still show L-DOPA responsiveness. AXO-Lenti-PD may provide advantages over DBS, despite the stereotactic procedures being similar. Firstly, AXO-Lenti-PD would remove the need for the implantation of an indwelling catheter, which presents potential issues with integrity, and a pulse generator located in the chest wall, which may present problems with infection and electrical hardware malfunction. Secondly, DBS is associated with a significant risk of AEs including difficulties with speech, expression, hallucinations and behaviour and social changes. If AXO-Lenti-PD demonstrates no clinical benefit, patients would still be eligible for DBS, although this protocol excludes patients who have previously received DBS.

5.6.3 Rationale for Key Endpoints

The key endpoints selected for assessing safety include standard endpoints such as incidence, causality and severity of AEs, vital signs, lab parameters and brain MRI assessments.

The key endpoints selected for signs of activity include, amongst others, standard questionnaires and scales used to assess PD, such as changes in the UPDRS scores for Parts I-IV, the dyskinesia rating scale, cognition and impulsivity. Changes in PD medications will also be assessed.

Several exploratory endpoints have been included, such as PET imaging, in the “OFF” medication state, using [^{11}C]–(+)-PHNO and an optional “ON” medication state [^{11}C]-(+)-PHNO scan.

The last exploratory endpoint is to conduct exploratory research on optional blood samples for PD-related research, specifically to assess biomarkers to study the expression profile of genes

known or suspected to be involved with PD, including but not limited to: *ATP13A2, GBA, LRRK2, PARK2, PARK7, PINK1, SNCA, UCHL1, VPS35, TH, DDC (Dopa Decarboxylase), GCH1 (GTP Cyclohydrolase 1) and COMT.* The objective of biomarker profiling is to enable development of treatments specifically targeted for optimal patient benefit.

5.6.4 Rationale for Dose

Table 3 below provides the intended dose and infusion volume of AXO-Lenti-PD for cohorts 1-3.

Table 3 AXO-Lenti-PD Dosing Parameters

Dose-Level	Total Volume (µL/patient)	Infusion Rate (µL/Min)	Target Titer (TU/mL)	Measured Titer [a] (TU/mL)	Total Dose [b] (TU/patient)
Cohort 1	600 µL	3	$7.0x10^6$	$4.2x10^6$	$2.6x10^6$
Cohort 2	600 µL	3	$2.3x10^7$	$1.5x10^7$	$9.0x10^6$
Cohort 3	1,800 µL	5	$2.3x10^7$	$1.5x10^7$	$2.7x10^7$

a. Measured concentration (i.e. titer; strength) of the manufactured lot used in clinical trial
b. Based on the actual measured titer

5.6.4.1 Dose and Infusion Volume Rationale for Cohort 1 and 2

The dose levels of AXO-Lenti-PD to be administered Cohort 1 and Cohort 2 were determined based on the tolerability of the concentrated vector (7.0×10^7 TU/mL) in the nonclinical macaque toxicology study (study PCS0171/HBW0003-00 in the IB). The titre of the AXO-Lenti-PD vector used in this study was 6.5×10^7 TU/mL and each animal in the AXO-Lenti-PD treatment group received a total dose of 1.3×10^7 TU (total volume of 200 µL/animal). The PD human brain volume is 21-fold greater than the macaque brain. Therefore, the total dose delivered to the macaque brain in the toxicology study is allometrically equivalent to a 2.73×10^8 dose ($1.3 \times 10^7 \times 21$) in the PD human brain. Similarly, the highest dose of vector that a patient could receive in Cohort 1 and Cohort 2 would be 4.4×10^7 and 1.3×10^7 TU respectively. These are 6.2-fold and 21-fold lower than the PD brain allometrically scaled dose used in the macaque toxicology study respectively. The proposed starting dose for the clinical study of 2.5×10^6 TU/patient (Cohort 1) and 9.0×10^6 TU/patient (Cohort 2) are less than the allometrically scaled lowest dose used in the NHP efficacy study thus providing a margin of safety (see Table 4). Safety margin for scaled infusion volume of 600 µL in Cohort 1 and Cohort 2 is shown in Table 5.

5.6.4.1 Dose Rationale for Cohort 3

The dose regimen for Cohort 3, was selected to yield increased distribution of vector within the target post-commisural putamen, by increasing the volume of each of the six infusion sites

3-fold from 100 µL per infusion to 300 µL per infusion site, that is an increase in infusion volume from 300 to 900 µL per hemisphere (based on 3 infusions in each putamen), increasing the total infusion volume per patient from 600 µL to 1,800 µL. The titer to be used for Cohort 3 remains the same as for Cohort 2, 1.5 x 10^7 TU/mL. The dosing approach for Cohort 3 is anticipated to provide a significant increase in overall putamenal vector coverage and to yield a greater volume of transduction and production of dopamine. Increase in putamenal coverage with a unilateral infusion volume of 900 µL per hemisphere has been previously shown to be safe and to likely improve outcomes in PD[34].

In order to support this higher volume of infusion at an appropriate putamenal scaled dose (7.2x scaling factor[35]), a second NHP study was carried out in cynomolgus animals (LNT-PD-1001). The titer of AXO-Lenti-PD for this study was 1.6 x 10^7 TU/mL yielding a total infused dose of 8.0 x 10^6 TU. Using putamenal volumetric scaling, the human dose equivalent taken from the second NHP study was 5.76 x 10^7 TU (7.2x scaling factor multiplied by 8.0 x 10^6 TU). The safety margin was calculated by dividing the scaled dose by the cohort 3 dose (2.7 x 10^7 TU) to yield a value of 2.13-fold. See Table 4 for a layout of these dosing values.

Likewise, a scaled volume of infusion (Vi) can be calculated from the NHP study LNT-PD-1001. Using a 7.2x scaling factor multiplied by the NHP Vi of 500 µL (total Vi) yields a human equivalent Vi of 3,600 µL. Dividing this value by the Vi from Cohort 3 (1,800 µL total Vi) gives a safety margin of 2.13-fold. Thus, the clinical dose for Cohort 3 is well below the scaled NHP human dose equivalent for both NHP studies. Moreover, the Vi for Cohort 3 was also below the scaled NHP human Vi indicating safety coverage was provided for dose and Vi based on the two NHP studies. See Table 5 for a layout of these Vi values.

In addition to modeling the dose and Vi for Cohort 3, the NHP LNT-PD-1001 also incorporated a change in the rate of infusion to match Cohort 3. The rate of infusion was increased from 3 µL/min to 5 µL/min, with this faster rate intended to shorten overall infusion and anesthesia time with the volumes to be used in Cohort 3.

Table 4 Scaled Doses and Safety Margins based on NHP Studies

				Human Brain Equivalent			
				Whole Brain (21x)		Putamen (7.2x)	
Dose-Level	Total Dose (TU/patient)	Non-Clinical Study Ref	Total NHP Dose (TU)	Scaled Dose (TU)	Safety Margin	Scaled Dose (TU)	Safety Margin
Cohort 1	$2.64x10^6$	PCS0171/HBW0003-00	$1.30x10^7$	$2.73x10^8$	103.41	$9.36x10^7$	35.45
Cohort 2	$9.00x10^6$	PCS0171/HBW0003-00	$1.30 x10^7$	$2.73x10^8$	30.33	$9.36 x10^7$	10.40
Cohort 3	$2.70x10^7$	PCS0171/HBW0003-00	$1.30 x10^7$	$2.73x10^8$	10.11	$9.36 x10^7$	3.47
Cohort 3	$2.70x10^7$	LNT-PD-1001	$8.00 x10^6$	$1.68x10^8$	6.22	$5.76 x10^7$	2.13

Table 5 Scaled Volumes of Infusion (Vi) and Safety Margins based on NHP studies

Clinical Dose Level	Total Vi (µL /patient)	Non-Clinical Study Ref	Total Putamenal NHP Vi (µL)	Scaled Vi (µL) (7.2x)	Safety Margin
Cohort 1 and 2	600	PCS0171/HBW0003-00	200	1,440	2.4
Cohort 3	1,800 µL	LNT-PD-1001	500	3600	2.0

5.6.5 AXO-Lenti-PD Administration

AXO-Lenti-PD is administered in small (100 µL to 300 µL) volumes at a controlled rate (3µL/minute or ramping up to 5 µL/minute) directly into the putamen of patients by local bilateral stereotactic infusion. The AXO-Lenti-PD Administration System was therefore designed to be capable of accurately delivering the precise small volume of AXO-Lenti-PD vector into a target area of the putamen at the controlled flow-rate.

The optimal specifications for administration of this targeted gene therapy were defined in non-clinical studies and further refined in the clinical study of the first generation product ProSavin®, (PS1/001/07 study). In this study, administration of small quantities of ProSavin® at the defined flow rate, using equipment previously determined in non-clinical studies was found to be safe and effective with no administration issues noted. The infusion system used in initial clinical study PS1/001/07 was comprised of components of laboratory equipment, which were purchased directly from the suppliers.

AXO-Lenti-PD will be administered to a similar patient population by an identical route to that shown to be safe and effective for the administration of ProSavin®. Accordingly, when determining appropriate equipment for the administration of AXO-Lenti-PD, it was important to design an administration system which met the essential performance requirements identified in relevant non-clinical and clinical studies and had similar design specifications to the components safely used previously.

No single CE marked medical device is currently known to be available which could meet the defined requirements. However, medical devices were identified that are currently used within the clinic, the majority of which are CE marked, which could be assembled to form an administration system, which could perform to the required specifications.

Full details of the AXO-Lenti-PD Administration System including risk assessment can be found in the AXO-Lenti-PD Administration System Device IB.

A Sponsor agent (or a representative thereof) may be present to observe during study protocol and/or surgical procedures.

5.6.6 Rationale for Study Duration

Per the European Medicines Agency (EMA) Guideline on Follow-Up of Patients Administered with Gene Therapy Medicinal Products[36], active clinical follow up should be undertaken with patients administered with gene therapy medicinal products, in order to detect signals of early or delayed adverse reactions, to prevent clinical consequences of such reactions, to ensure timely treatment and to gain information on the long-term safety and efficacy of the interventions. The recommended follow-up for a gene therapy such as AXO-Lenti-PD would be at 3, 6 and 12 months after treatment, and then yearly thereafter for a minimum of 5 years. This study incorporates this, but will extend the follow-up period to 15 years. In order to balance the imposition on patients and clinicians, patients will only be required to visit the clinic for the first 5 years after treatment. Thereafter, patients will only be contacted by phone and sent questionnaires annually to collect any safety and efficacy data, however, patients will be encouraged to attend the clinic for assessments at years 10 and 15 if possible (but will be given the option to conduct a limited assessment by phone). Patients will be asked to consent to the sponsor contacting the site staff annually after 15 years to determine each patient's survival status.

5.6.7 Summary of Overall Risks and Benefits

Although the CNS is regarded as immune-privileged in healthy individuals, physical barriers and tolerance mechanisms that are utilised to prevent and suppress any inflammatory immune responses are not fully understood, and may be affected by disease, age, injury (e.g. surgery) and the presence of foreign material. The infusion procedure used to administer AXO-Lenti-PD may breach capillaries and therefore result in exposure of AXO-Lenti-PD components to

the peripheral immune system of a recipient. Administration of AXO-Lenti-PD could induce immune responses against components of the vector that could potentially result in adverse effects (e.g. inflammation), and/or reduce efficacy.

As ProSavin® was delivered by a similar surgical procedure and expresses the same genes as AXO-Lenti-PD, benefit-risk data compiled from the nonclinical and clinical development of ProSavin® are considered relevant to AXO-Lenti-PD. There are no data from nonclinical studies with AXO-Lenti-PD (or ProSavin®) that are considered to indicate a potential impact on patient safety in the proposed clinical setting. Clinical development of ProSavin® has been completed with one Phase I/II study conducted in France and the UK and a follow-up study is ongoing. Safety data on the administration of ProSavin® are available in 15 patients for a minimum of 36 months, a minimum of 7 years for the 10 ongoing patients and up to 10 years of exposure for some patients. Overall ProSavin® has been safe and well tolerated with one SUSAR of worsening dyskinesias reported. Up to 11th December 2018, a total of 38 SAEs have been reported to date during the ProSavin® clinical development programme; one SAE in PS1/001/07 and 37 in PS1/001/09, of which 3 had a fatal outcome. No SAEs were considered to have a reasonable possibility of a causal relationship to the investigational product or investigational procedures by the reporting Investigators and/or OXB. There have also been no indications of any late-onset AEs related to ProSavin®.

Development of ProSavin® was discontinued due to uncertainty over clinically meaningful responses at the dose levels which it was possible to study. The long-term follow-up study continues to capture data for 10 years on the enrolled patients. Overall, the vast majority of AEs believed to be IMP related have been in the nervous system SOC and mild to moderate in severity. Of the reported AEs in the nervous system SOC the most frequent were dyskinesia and “ON” and “OFF” phenomena, which abated when the dose of oral dopaminergic therapy was adjusted.

Currently, there is no evidence of any local immunological or inflammatory reaction to ProSavin® as assessed by direct imaging on MRI. An antibody response against VSV-G was observed in 4 out of the 6 patients in PS1/001/07 Cohort 3 but the patients were asymptomatic at the time. Analysis of blood and urine has not detected any consistent RNA or DNA from ProSavin®.

The Phase I/II study (PS1/001/007) was terminated early when the DMC recommended the exploration of higher doses potentially to improve efficacy. After further review of the longer term data from the PS1/001/09 study the DMC acknowledged encouraging signs of biological activity but their recommendation to explore higher doses did not change. For those patients already dosed, one drug-related SAE of worsening dyskinesias was noted and the AE profile broadly reflects the underlying disease and its treatment. The benefit/risk for those patients already treated with ProSavin® was considered positive and is expected to be similar or more beneficial for patients treated with AXO-Lenti-PD.

A full risk assessment has been conducted on the AXO-Lenti-PD Administration System which is described in the AXO-Lenti-PD Administration System device IB. In summary, it was

considered by OXB and accepted by Axovant Sciences after examination and final assessment by the involved investigators/neurosurgeons that the risk/benefit balance remains favourable for use of the administration system.

AXO-Lenti-PD is to be administered to patients with bilateral, idiopathic PD who are inadequately controlled by oral therapies. There is no standard treatment established for these later stage patients and available treatment options for these patients are limited, typically involving invasive surgical therapies to control motor functions and to reduce hypomobility episodes including DBS, Duodopa® (levodopa/carbidopa intestinal gel) and apomorphine (dopamine agonist) pumps. Treatment with AXO-Lenti-PD may provide long term constant and local dopamine replacement to the striatum and thereby restore beneficial movements in the absence of central and peripheral side effects. By providing a more continuous putamenal dopamine supply, motor side effects associated with the delivery of exogenous L-DOPA may be avoided. The level of dopamine replacement mediated by AXO-Lenti-PD may be sufficient to permit a decrease in the overall dose of the L-DOPA regimen and provide a sustained motor correction with longer "ON" periods and shorter "OFF" periods. Accordingly, treatment with AXO-Lenti-PD may provide a long-term clinically significant benefit to patients with limited treatment options.

Based on current pre-clinical studies AXO-Lenti-PD may to be 5 to 10 times more potent than ProSavin®, however its potency in human is unknown. As this is a first in human study, a cautious approach has been adopted where a 10-fold increase in potency has been assumed. The starting dose is therefore anticipated to be one which is safe, well tolerated and one which is similar to the mid dose used in the completed ProSavin® study. This initial dose is anticipated to provide a minimal expected biological effect level in humans thus providing a modest effect on PD symptoms. However, if the potency in the clinic is less potent, then the starting dose will be similar to the lowest dose used in the completed ProSavin® study which whilst allowing an even greater margin for safety could potentially be a sub-therapeutic dose. It is important not to expose patients to sub-therapeutic doses and therefore the first dose cohort will enrol a minimum of 2 patients who will be evaluated at 3 months for safety and tolerability before possible dose escalation to the next dose cohort (or enlargement of the current cohort).

Given the potential of AXO-Lenti-PD to exceed the encouraging signs of clinical effect reported with ProSavin®, the reassuring safety profile reported from patients undergoing treatment with ProSavin® to date and the conservative approach to dose escalation, the benefit:risk for patients treated with AXO-Lenti-PD using the AXO-Lenti-PD Administration System is considered to be positive.

Participants taking part in the PET sub-study will be exposed to increased ionising radiation due to the administration of [^{11}C]-(+)-PHNO and the low dose CT scans acquired to aid with the analysis of the PET data. Participation in this study will lead to a maximal exposure of 7.38 mSv (4.86 mSv from up to 6 [^{11}C]-(+)-PHNO PET scans of up to 180 MBq each, and 2.52 mSv from up to 7 low dose CT scans of the head of up to 0.36 mSv each). These figures include exposure from 2 required PET and CT scans, 4 optional PET and CT scans, and a possible

CT scan on the day of surgery. This exposure is equivalent to approximately 3 times the average background exposure for UK residents (2.3 mSv per year). While any additional radiation exposure increases the risk of future malignancy, the additional risk due to the radiation exposure in this study is low. Exposure to ~7.4 mSv in a healthy 40-year old will lead to an estimated risk of 1:2700 of future malignancy. When contrasted with the average life-time risk of malignancy for UK residents (1:3), this additional risk is low. As the risk of future malignancy is proportional to the life-expectancy following exposure, and as study participants have a reduced life expectancy compared to a healthy 40-year old, the additional risk due to ionising radiation exposure in this population is expected to be lower than 1:2700.

(+)-PHNO is a potent dopamine D_2/D_3R agonist and can lead to incidences of nausea and vomiting even when administered in very low amounts. For this reason, the maximal mass of (+)-PHNO administered at each PET scans will not exceed 30 ng/kg. Past experience indicates that doses below 30 ng/kg do not lead to nausea and vomiting in participants.

Although (+)-PHNO does not have any therapeutic benefit for Parkinson's disease patients, [^{11}C]-(+)-PHNO PET can potentially provide important scientific evidence for the biological effect of AXO-Lenti-PD and the correlation between the biological effect and clinical benefits. As the risks of [^{11}C]-(+)-PHNO PET scans are low, using them in the PET sub-study will not significantly alter the benefit:risk of AXO-Lenti-PD.

6 STUDY OBJECTIVES

6.1 Primary Objectives

The primary objective of this study is:

- To evaluate the safety and tolerability of AXO-Lenti-PD treatment in patients with bilateral, idiopathic, advanced stage Parkinson's disease

6.2 Secondary Objectives

The secondary objectives of this study are:

- To determine the optimal dose of AXO-Lenti-PD
- To evaluate any signs of activity of AXO-Lenti-PD
- To evaluate the long term safety of AXO-Lenti-PD

6.3 Exploratory Objectives

The exploratory objectives of this study are:

- To evaluate any changes in dopamine levels with the use of $[^{11}C]$-(+)-PHNO PET BP_{ND} and their correlation with changes in clinical assessments
- To conduct exploratory research on optional blood samples for PD-related research, including but not limited to genomic analysis
- To explore treatment impact on novel questionnaires

7 STUDY ENDPOINTS

7.1 Primary Endpoint

- The incidence, causality and severity of adverse events, and measurements of other safety parameters (laboratory parameters, vital signs, brain MRI, physical examination, C-SSRS) at 3 months

7.2 Secondary Endpoints

The primary time point for secondary endpoints is 6 months unless otherwise specified. These endpoints are listed below.

- The change in UPDRS scores for each part compared to baseline in defined “OFF” and “ON” medication states
- The change in time during the waking day the patient is in the “OFF” state compared to baseline as assessed by Hauser patient diaries
- The change from baseline in the patients’ total score on activity of daily living using the PD Questionnaire 39 (PDQ-39)
- The change in health-related quality of life using the EuroQOL five dimensions questionnaire (EQ-5D-5L)
- The change in dyskinesia rating scale from baseline using the Rush Dyskinesia Rating Scale (RDRS)
- The change from baseline in impulsivity using the Questionnaire for Impulsive-Compulsive Disorders in Parkinson's Disease (QUIP-RS)
- The change in Clinical Global Impression (CGI) Scale from baseline
- The change from baseline in dementia using the Mattis Dementia Rating Scale-2™ (DRS-2)
- The change from baseline in the Hoehn and Yahr Scale in “ON” and “OFF” states
- The change from baseline in cognition using the Montreal Cognitive Assessment (MoCA)
- The change from baseline in depression/mood using the Hospital Anxiety and Depression Scale (HADS)
- The change from baseline in apathy using the Starkstein Apathy Scale
- The change from baseline in the L-DOPA Equivalent Daily Dose (LEDD)
- The change from baseline in L-DOPA dose
- Immunology: the incidence of humoral responses to AXO-Lenti-PD administration

7.3 Biodistribution Endpoint

- Potential distribution in the blood and urine of AXO-Lenti-PD as assessed by quantitative polymerase chain reaction (qPCR) analysis

7.4 Exploratory Endpoints

- The percentage change from baseline in the dopamine levels using [^{11}C]-(+)-PHNO PET BP_{ND} at 6 months and optionally at 12 months ("OFF" state). Optional "ON" medication state scans may also be conducted at baseline, 6 and 12 months.
- Correlation between the percentage change from baseline in the dopamine levels using [^{11}C]-(+)-PHNO PET BP_{ND} at "OFF" state and various clinical endpoints including the percentage change from baseline in UPDRS scores at "OFF" state and other clinical assessments
- Area Under the Curve (AUC) analysis of response to Levodopa challenge
- The change from baseline in the Non-Motor Symptom Scale (NMSS)
- The change in Patient Global Impression (PGI) Scale from baseline
- Exploratory research on optional blood samples for PD-related research, including but not limited to genomic analysis (baseline only)

8 STUDY DESIGN

8.1 Overview of Study Design

This first in man, phase I/II study has been split into two parts in order to evaluate safety, tolerability and the optimal dose of AXO-Lenti-PD in this study in the described patient population:

Part A: open label, dose escalation design to determine safety and tolerability of AXO-Lenti-PD, and to establish the optimal dose (from three dose levels) in the study patient population.

Part B: double-blind design with patients randomised to an active group (optimal dose AXO-Lenti-PD found in Part A) or a control group (an imitation surgical procedure) in a 2:1 ratio.

Up to 18 patients will be enrolled in Part A (depending on the number of dose levels evaluated and the number of patients per dose level), a minimum of 2 patients will be enrolled in Cohort 1, a minimum of 4 patients will be enrolled in Cohort 2, and a minimum of 2 patients will be enrolled in Cohort 3. Each cohort may be expanded to 6 patients on the recommendation of the SMC (per Table 6). Up to 12 patients will be enrolled in Part B, up to 8 of those patients receiving AXO-Lenti-PD and up to 4 patients with an ISP only.

The overall duration of the study will continue for 15 years post-treatment to assess long term safety and tolerability.

The schematic of the study design is shown in Section 4.

Part A: Open labelled dose escalation

Three dose level cohorts will potentially be explored in the dose escalation part of the study (see Table 6).

During patient enrolment through the dose escalation phase, the individual patient safety data will be reviewed on an ongoing basis by the Medical Monitor and the sponsor via safety reports generated through the eCRF as well as from the pharmacovigilance vendor. All relevant safety information will be disseminated to participating sites together with ongoing written notifications indicating when the next patient/s can be screened.

<u>Cohort 1</u>

At the lowest dose level (Cohort 1), 2 patients will initially be sequentially enrolled and treated a minimum of 28 days apart to allow each patient to be observed for short-term safety and tolerability.

Once 2 patients in cohort 1 have been followed for a minimum of 3 months post treatment, all available data will then be reviewed by the SMC. After review of the data, the SMC could recommend to expand the dose level to include an additional 2 patients (who could be treated in parallel rather than sequentially on the SMC recommendation), to progress to the next

cohort, to move to Part B or to suspend enrollment and request further post-treatment data or to stop any further dosing in the study.

If further patients are to be recruited, the SMC will reconvene to review a minimum of 3-months post-treatment data on the additional patients in conjunction with all available data on the ongoing patients. After review of all available data the SMC could then recommend to include a further 2 patients treated in parallel (i.e. total maximum of 6 patients), to progress to the next cohort, to move to Part B, to suspend enrollment and request further post-treatment data or to stop any further dosing in the study. If further patients are to be recruited, the SMC will reconvene to review a minimum of 3 months post-treatment data on the additional patients in conjunction with all available data on the ongoing patients.

<u>Cohort 2</u>

If the mid dose level (Cohort 2) is to open, the first patient will be observed for short-term safety and tolerability for a minimum of 28 days after treatment with this dose level and then 3 more patients will be treated in parallel (i.e., 4 patients in Cohort 2). Once 4 patients at dose level 2 have been treated the SMC will reconvene to look at the corresponding data (at this dose level, a minimum of 3 months safety data will be available on all patients) but also the available ongoing data from patients in the lowest dose level 1. The SMC could then recommend that the study progresses as above (i.e. expand the dose level 2 cohort to 6 patients (which can be treated in parallel), proceed to Cohort 3, move to part B, to suspend enrollment for further post-treatment data or stop the study). If further patients are to be recruited for a total of 6 patients in Cohort 2, the SMC will reconvene to review a minimum of 3 months post-treatment data on the additional patients in conjunction with all available data on the ongoing patients.

<u>Cohort 3</u>

If the high dose Cohort 3 is to open, the first patient treated at this dose level will be observed for 28 days after treatment to assess safety and tolerability and then an additional patient will be treated. The SMC will meet to review a minimum of 3 months post-treatment data from these first two patients in Cohort 3 along with the available ongoing data from Cohort 1 and cohort 2 as described in the SMC charter. After review of the data, the SMC could recommend to expand the dose level to include an additional 2 patients (who can be treated in parallel), to open Part B or to suspend enrollment and request further post-treatment data or to stop any further dosing in the study.

If further patients are to be recruited, the SMC will reconvene to review a minimum of 3 months post-treatment data on the additional patients in conjunction with all available data on the ongoing patients. After review of all available data the SMC could then recommend to include a further 2 patients treated in parallel, to open Part B, to suspend enrollment and request further post-treatment data or to stop any further dosing in the study. If 2 additional patients are to be recruited in Cohort 3, the SMC will reconvene to review a minimum of 3 months post-treatment data on the additional patients in conjunction with all available safety data on the

ongoing patients. After review of all available safety data, the SMC may then recommend to open Part B, include a further 2 patients treated in parallel (i.e. total maximum of 6 patients in Cohort 3), suspend enrollment and request additional post-treatment safety data, or to stop any further dosing in the study.

Patient recruitment will be suspended by the Sponsor and further dosing within a cohort temporarily halted if the study meets study stopping criteria detailed in Section 8.4.8.

All patients from the dose escalation Part A will continue to be assessed in the long-term follow up according to the study schedule in Section 3.

Part B: Double-blind, randomised to active (optimal dose of AXO-Lenti-PD from Part A) or control (ISP)

This part of the study will only open once the SMC have determined there are sufficient data on safety from the patients in the dose escalation part of the study. Based on this safety recommendation, Sponsor will determine the optimal safe dose suitable for further evaluation by reviewing all available data for safety and signs of activity. Twelve patients will then be enrolled in a double blind (patient and assessor blinded, but not the surgical team), randomised (2 active:1 control) manner, and patients may be enrolled in parallel. Eight patients will receive the selected dose of AXO-Lenti-PD and four patients will receive ISP (without bone and dural penetration).

Table 6 Dosing Regimen

Study Part	Cohort	No of treated Pts	No of Pts for Imitation Surgical Procedure	Concentration of vector (Titer)[1]	Total Infusion Volume	Total Dose/Patient (TU)[2]
A	1	2 – 6	Not applicable	4.4×10^6	600 µL (300 µL per hemisphere)	2.6×10^6
	2	4 – 6	Not applicable	1.5×10^7	600 µL (300 µL per hemisphere)	9.0×10^6
	3	2 – 6	Not applicable	1.5×10^7	1800 µL (900 µL per hemisphere)	2.7×10^7
B	Active treatment: Dose level from Cohort 1, 2 or 3 (to be determined from Part A)	8	Not applicable	Concentration as determined from Part A	600 or 1800 µL (300 or 900 µL per hemisphere)	Dose as determined from Part A
	ISP control	Not applicable	4	Not applicable	Not applicable	Not applicable

[1]Vector concentrations (i.e. titers) are based on measurement of vector lots manufactured for clinical use
[2]Doses are calculated based on the measured titers

8.2 Schedule of Assessments and Study Duration

All patients must provide consent prior to any trial-related procedures, and will then undergo a panel of screening tests over a period of up to 60 days prior to surgery (see study schedule, Section 3).

8.2.1 Part A

Part A patients will undergo the surgical procedure to administer AXO-Lenti-PD on Day 1. These patients will remain in hospital for at least 72 hours postoperatively and then be followed up at Day 8, Day 15, and Month 1, 3, 6, 9 and 12, after which they will enter the long term follow up phase of the study. This will comprise assessments every 6 months in the second year after surgery, then annual assessments to 15 years from the date of surgery. The patients will need to attend the clinic for follow up visits occurring in the first 5 years, but after that, the patients can be followed up via a phone call or written questionnaire for the subsequent 10 years although will be encouraged to attend the clinic for assessments at years 10 and 15, if possible. This long term follow up will assess long-term safety and long term signs of activity. After this active follow-up phase of data collection, no further study visits or phone calls for the patient are required, although the site staff will be contacted annually by the sponsor to determine each patient's survival status.

For study visits where assessments need to be performed with the patients in the “OFF” medication state, the patients will need to be hospitalised the night before the assessments and their PD medication withdrawn temporarily (see Section 8.4.9.2).

8.2.2 Part B

Part B active group patients randomised to receive study treatment AXO-Lenti-PD: these patients will follow the same process as the Part A, Cohort 3 patients above (see Section 8.2.1 and Section 10.5) but will be unaware of their treatment until the blind is broken.

Part B control group patients randomised to receive ISP: these patients will undergo all the screening and follow-up procedures as for the Part A patients above, but instead of having AXO-Lenti-PD administered will undergo an ISP and will be unaware of their treatment until the blind is broken. Each patient will be admitted to hospital the day before surgery and on Day 1 undergo the fixation of the stereotactic frame. All ISP patients will undergo general anaesthesia but only bilateral skin incisions at locations guided by the neuronavigation system under strict aseptic conditions will be performed. To reduce the risk of infection, no holes will be drilled in the skull. As the burr holes will be sealed with Craniosorb Absorbable Fixation System (AFS, Codman®, USA or equivalent) and covered with a titanium implant in patients who receive AXO-Lenti-PD, Craniosorb AFS (or equivalent), and titanium implant will be placed over the bone at the skin incision site during the ISP in order to ensure blinding is maintained during postoperative imaging studies. Details of procedure are included in Surgical Manual.

The total surgery time for AXO-Lenti-PD treated patients (including the preparation of the scalp, and stereotactic frame attachment and MRI/CT) is expected to be approximately eight hours if dose volume from Cohorts 1 or 2 is selected, and twelve hours if dose volume from Cohort 3 is selected (of which the actual administration of AXO-Lenti-PD is likely to take approximately four to five hours if dose volume from Cohorts 1 or 2 is selected, and six to seven hours if dose volume from Cohort 3 is selected). To maintain blinding of the patient, their relatives and other hospital staff, the patient undergoing ISP may remain in the operating theatre for the same duration. All patients will then be transferred to the neurosurgical intensive care unit and monitored according to specific hospital policies and procedures for at least 72 hours post-surgery.

The blinding of Part B of the study will be maintained until all Part B patients have reached the 6 month post-surgery assessment visit and the corresponding data analysed. At the time of unblinding, those patients who received ISP will be considered for AXO-Lenti-PD treatment should the patients still be suitable for surgery, considered medically appropriate and should the SMC, relevant regulatory and ethics committees consider it safe and potentially of benefit. If such patients consent and are suitable to receive AXO-Lenti-PD treatment they will be followed under a separate protocol.

8.3 Patient Selection

Patients will only be recruited after all necessary approvals (e.g. ethics, regulatory, R&D committees) have been received in writing. Entry criteria into the study will be assessed during the screening period and where appropriate, checked the day before surgery. An Enrolment Authorisation Committee (EAC) will be responsible for reviewing and approving each subject prior to AXO-Lenti-PD administration or ISP to ensure that each subject meets entry criteria, has the correct diagnosis, and in addition, has data that are consistent and is a suitable candidate for the study. Subjects cannot be enrolled without EAC approval. The details of the review will be specified in the EAC Charter.

8.3.1 Inclusion Criteria

Patients must meet ALL of the following criteria to be considered for enrolment into the study:

1) Diagnosed with bilateral idiopathic PD, using the UK Parkinson's Disease Society (PDS) Brain Bank Criteria, but without the specific exclusion of patients with more than one affected relative
2) Males/females between 30 and 70 years at the time of surgery
3) Presence of motor fluctuations and/or dyskinetic movement
4) Affiliated with the French, UK or equivalent social security health care system

5) Candidate for surgical intervention because of disabling motor complications and willing to defer this until at least after the 12 month study visit (unless medically recommended)

6) Freely given signed and dated written informed consent obtained from the patient/legal representative prior to performing any protocol-related procedures, including screening evaluations

7) **For Women**: Women of nonchildbearing potential who meet at least 1 of the following criteria:

    b. Achieved postmenopausal status, defined as cessation of regular menses for at least 12 consecutive months with no alternative pathological or physiological cause

    c. Documented hysterectomy with bilateral oophorectomy

    d. Medically confirmed ovarian failure

    Women who are not postmenopausal (as defined above) must have:

    e. Negative pregnancy test and must not be lactating at the Screening and Day -1 visits

    f. Must agree to practice two effective methods of birth control, including at least one barrier method and one highly effective method, and must agree to continue using such contraceptive precautions for at least the first 3 months of the study following AXO-Lenti-PD administration. Highly effective forms of birth control include:

    - Combined (oestrogen and progestogen containing) hormonal contraception associated with inhibition of ovulation: oral, intravaginal, or transdermal
    - Progestogen-only hormonal contraception associated with inhibition of ovulation: oral, injectable, or implantable
    - Intrauterine device (IUD)
    - Intrauterine hormone-releasing system (IUS)
    - Bilateral tubal occlusion
    - Vasectomized partner
    - Sexual abstinence (true abstinence only; periodic abstinence [e.g. calendar, ovulation, symptothermal, post-ovulation methods] and withdrawal are not acceptable)

    For Men: Males, unless surgically sterile, must agree in conjunction with their female partner to use two effective methods of birth control if their partner is of childbearing

potential, including at least one barrier method, and must agree to continue using such contraceptive precautions for at least the first 3 months of the study following AXO-Lenti-PD administration

8) Willing to be hospitalised and have current PD treatment withdrawn for up to 24 hours (or up to 48 hours for long lasting agonists, such as ropinirole MR, pramipexole PR, or cabergoline) for each visit where patients need to be assessed in the “OFF” medication state
9) UPDRS Part III score of between 30 and 60 in the “OFF” medication state
10) Positive response to dopaminergic therapy as defined by at least a 40% improvement in UPDRS (Part III) between the “OFF” and “ON” medication states at Screening
11) Hoehn and Yahr (H&Y) Stage 3 or 4 in the “OFF” medication state at Screening
12) Stable dosing of PD medication, including L-DOPA, for four weeks prior to the first day of Screening with the L-DOPA Equivalent Daily Dose (LEDD) being ≥ 900 mg
13) Experiencing at least 2.5 hours of “OFF” time confirmed by the Hauser patient diary for each of 3 consecutive days anytime during Screening. Patients must demonstrate ability to accurately recognise their “ON” and “OFF” states after appropriate training as verified by successfully meeting concordance testing criteria. Patients must be compliant (with no missing data > 2 hours per day) for at least 2 consecutive days for the Screening visit. The “OFF” time inclusion criterion must NOT be shared with patients or their caregivers prior to and during Screening
14) Clearance by an Enrollment Authorization Committee (EAC) comprised of Parkinson’s disease clinical experts

8.3.2 Exclusion Criteria

Any of the following would exclude the patient from being enrolled into the study:

1) Any condition that, in the opinion of the investigator and/or EAC, would interfere with evaluation of the IMP or interpretation of patient safety or study results
2) Patients unable or unwilling to adhere to their prescribed PD medication regimen or the requirements of the study in the opinion of the investigator
3) Life-threatening illness unrelated to PD at the time of surgery
4) History of malignancy within five years or a positive cancer screening test within one year prior to screening visit (with the exception of in situ, non-melanoma forms of skin cancer)

5) History of epilepsy or any other co-morbid condition that the Investigator believes presents an unacceptable health risk to the patient in conjunction with the procedures in this protocol, including the surgery
6) History of psychosis or current treatment with dopamine blocking agents of any kind (e.g., metoclopramide, prochlorperazine, lithium and sodium valproate) and prior regular exposure to antipsychotic agents (e.g., chlorpromazine, clozapine, quetiapine, risperidone)
7) Concurrent treatment for depression that targets the dopaminergic pathways.
8) Concurrent anti-retroviral therapy that would inactivate the investigational agent
9) Alcohol or other substance abuse within five years prior to screening visit
10) History of stereotactic or other surgery for the treatment of PD, including Deep Brain Stimulation (DBS)
11) Participation in a prior cell or gene transfer therapy study
12) Planned participation in any other clinical study, for any condition, including those relating to PD, during the first 12 months of the study
13) Treated with dopaminergic antagonists within six months prior to screening
14) Prior treatment with tolcapone, within the six months prior to screening into the study, due to its ability to modify dopaminergic pathways within the brain
15) History of treatment with any agent that may induce PD or PD symptoms within the last three months prior to screening
16) Any contraindication for undergoing a Magnetic Resonance Imaging (MRI) scan of the head
17) Contraindications to use of anaesthesia
18) Current or anticipated treatment with anticoagulant therapy or the use of anticoagulation therapy that cannot be temporarily stopped around the time of surgery
19) Any known allergy to any component of the IMP or protocol required medication
20) Severe disabling dyskinesias ≥ 51% of the day as defined by the UPDRS (Part IV) at Screening
    - Note: The dyskinesia time exclusion criterion must NOT be shared with patients or their caregivers prior to and during Screening
21) Patients with L-DOPA resistant tremor
22) Diagnosis of multiple system atrophy following assessment of the autonomic nervous system function and MRI during the screening process (historical diagnosis not formal testing)

23) Abnormal MRI findings such as mega cisterna, septum pellucidum, signs of severe cortical or subcortical atrophy, brain tumours, vascular diseases, trauma or arteriovenous malformations

24) Clinically significant abnormalities in laboratory tests (including full blood count, chemistry panel, liver function tests), ECG and/or chest X-ray, identified during the screening period that in the opinion of the investigator would make the patient unsuitable for participation in the study

25) Presence of dementia, as diagnosed using the Mattis Dementia Rating Scale-2™ (DRS-2)

26) Intercurrent illness or severe infection 28 days prior to AXO-Lenti-PD administration

27) Major surgery within the 28 days prior to AXO-Lenti-PD administration

28) History of any investigational agent within 3 months prior to AXO-Lenti-PD administration

29) Inability to understand the informed consent or to comply in full with the requirements of the study

30) Significant suicide risk as defined by
    - Suicidal ideation as endorsed on items 4 or 5 on the C-SSRS within the past year at Screening of this study, or
    - Suicidal behaviours within the past year endorsed on the C-SSRS at Screening of this study, or
    - Clinical assessment of significant suicidal risk

8.4 Patient Enrolment and Randomisation

8.4.1 Treatment Assignment

Each patient in Part A who meets the eligibility criteria will be assigned open-label IMP at the dose level ongoing at the time they are screened and enrolled into the study. Patient enrolment in Part A will be controlled by registering the patient on the eCRF and with Axovant Sciences to ensure adequate control is maintained of sequentially treating the patients across the sites. When a patient has been registered at screening in the eCRF, an automatic notification from the eCRF will be sent to Axovant Sciences to enable patient tracking across sites into the study. During Part A of the study, Axovant Sciences will provide participating sites with written authorisation before a patient can be treated and will ship IMP on a per patient basis (to ensure that patients are treated sequentially across the sites) in accordance with Section 10.1.

Each patient enrolled into Part B will be randomised at a 2:1 ratio to receive either AXO-Lenti-PD or ISP using a computer-generated schedule and Interactive Voice and Web Response System (IxRS).

8.4.2 Blinding for Part B only

Part B is a double-blind design, where the surgical procedures for the AXO-Lenti-PD treated group compared to the ISP group are clearly different, thus the neurosurgeon and operating staff involved in the surgical procedure will have to be unblinded. None of the unblinded surgical team may be involved in any way in the management or follow up of the patients post-surgery. It will also be necessary for the pharmacy staff to be unblinded due to the dispensing of the vials to the theatre staff and to handle returned vials. Further details are provided in the Pharmacy Manual and Surgical Manual together with details of considerations of how to maintain the blind in these circumstances. The rest of the investigational team who are involved in the treatment or clinical evaluation of the patients, the patient/legal representative and sponsor staff (and delegated study monitors and vendor/contract research organisation (CRO) staff) must remain blinded. An exception will include an independent monitor to perform IMP accountability.

In the event that the treatment allocation for a patient becomes known to the investigator or other study staff involved in the management of study patients the sponsor must be notified as soon as possible by the investigator.

The blinding of Part B of the study will be maintained until all Part B patients have reached the 6 month post-surgery assessment visit and the corresponding data analysed.

8.4.2.1 Unblinding in the Event of a Medical Emergency

In the event of a medical emergency, the investigator may unblind an individual patient's treatment allocation via the IxRS system or through the pharmacy. Prior to unblinding the IMP allocation for an individual patient, the investigator may choose to contact the sponsor Medical Monitor to discuss the medical emergency and the reason for wanting to unblind. If the investigator unblinds without discussion with the Medical Monitor, they must inform the sponsor as soon as possible thereafter. Instructions for unblinding an individual patient's treatment allocation are contained in the IxRS user manual. In general, unblinding should only occur if management of the medical emergency would be different based on the patient having received the active treatment. In the majority of cases, the management of a medical emergency would be the same whether or not IMP was received by the patient. If this was the case, the treatment allocation should not be unblinded.

8.4.2.2 Unblinding for SMC Reviews

During Part B of the study, the SMC will be provided with unblinded data, and the sponsor will only be provided with blinded data. Unblinded listings and summaries will be submitted periodically to the SMC. The timing of the SMC submissions will be specified in the SMC Charter.

8.4.2.3 Unblinding for SUSAR Expedited Reporting

SUSARs for reporting to EudraVigilance and Competent Authorities, as required, will be unblinded by the pharmacovigilance providers and unblinded data will be provided to the regulatory authorities. This information will not be shared with the sponsor or any of the study team.

8.4.2.4 Unblinding of AEs concerning the AXO-Lenti-PD Administration System

Any AE that is deemed related to the AXO-Lenti-PD Administration System will automatically unblind the patient concerned as events related to the ISP will be distinguishable. Any AE occurring during surgery (serious or non-serious) that is deemed related will therefore be handled only by the surgical team and by unblinded monitors and the pharmacovigilance provider unblinded team. Only blinded data will be shared with the sponsor or any of the blinded study team. It is acknowledged that post-surgical AEs related to the AXO-Lenti-PD Administration System may be difficult to manage and considerations of maintaining the blind will be further provided in the study procedure manuals.

8.4.3 Withdrawal Criteria

A patient has the right to withdraw from the study follow up assessments at any time and for any reason without prejudice to their future medical care.

However, once AXO-Lenti-PD has been administered bilaterally to the motor putamen region of the brain it cannot be removed, therefore patients are unable to truly withdraw from treatment although they can withdraw consent from any further study related visits/procedures. If a patient withdraws early from the study follow up assessments then the investigator will ask the patient to return to determine if the patient would agree to limited follow-up in order to comply with guidance from the EMA on follow up for clinical trials involving Advanced Therapy Medicinal Product (ATMPs).

The reason for a patient's withdrawal from the study, if this is provided by the patient, should be clearly documented in the patient's notes. If the patient agrees to return to discuss with site staff a limited follow-up, this discussion should be documented in the patient's medical records and should state what limited follow up the patient would agree to. A separate brief informed consent form should be discussed with the patient, and if they agree to a limited follow up, the details of this must be specified on the consent form.

Withdrawal of full consent: If full consent is withdrawn, the patient will not undergo any further study procedures or further study observation and will be withdrawn from the study. No further follow up will be permitted.

Patient has indicated their wish for a reduced follow up: If a patient no longer wishes to attend study site visits but has agreed either to be contacted by phone for a health status update (e.g. review of AEs, concomitant medications and any signs of efficacy) or to allow the

study team to contact their primary healthcare provider for updates, then the patient will be asked to document the details of the limited follow up they have agreed to on a separate brief informed consent form which they will need to sign.

Lost to follow-up: The study team should document attempts to re-establish contact with any missing patient at each scheduled visit. If contact is re-established the patient should resume visits according to the protocol. Patients will be considered lost-to-follow-up only if no contact has been established by the time the study is completed such that there is insufficient information to determine the patient's status at the Year 15 time point.

8.4.4 Replacement/Re-screening of Patients

It is not expected that patients will be replaced. However, patients may be replaced if withdrawal during the first 6 months is clearly unrelated to treatment. For example, if the patient has moved away and can no longer attend hospital visits or if the patient has died due to a reason that is clearly not treatment related. Any replacements must be discussed first with Axovant Sciences and no more than 2 patients will be permitted to be replaced within the study.

Any patients who screen fail on a criterion which at a future time point the investigator anticipates they would be able to pass, e.g. low haemoglobin, may be retested on the assessment that did not pass. For criteria related to Hauser patient diary, patients who screen fail only on compliance may be re-screened. Patients may only be re-screened on one occasion.

8.4.5 Patient Completion

An individual patient will be considered to have completed the study if the patient dies or was followed up through to the end of the study, defined as the Year 15 follow up.

8.4.6 End of the Study

The end of the study ("study completion") is defined as the date of the last protocol-specified visit/assessment (including telephone contact) for the last patient in the study. This date will be, at the latest, that of the last Year 15 contact to be conducted. All materials or supplies provided by the sponsor will be returned to the sponsor or designee upon study completion, as directed by the site monitor. The investigator will notify the Institutional Review Board (IRB)/ Independent Ethics Committee (IEC) when the study has been completed and the sponsor will notify the regulatory authority.

8.4.7 Annual Follow-up for Survival

After each patient's last study visit, they will be followed up annually for life by the site as part of their PD standard of care. Data collected during these post-study follow-up assessments will not be communicated to the sponsor except for the patients' survival data.

8.4.8 Study Stopping Criteria

Safety data is reviewed regularly by the sponsor and SMC. All events will be reviewed by the SMC as outlined in the charter (see Section 12). If any of the following occur in the study, administration of IMP will be temporarily halted and recruitment suspended:

1) Death of any patient in which the cause of death is assessed as related to IMP or the AXO-Lenti-PD Administration System.
2) Anaphylactic reaction to IMP in any patient
3) An SAE in any patient where the event is assessed as related to IMP or the AXO-Lenti-PD Administration System.
4) Any severe AE in any two patients that are considered related to IMP or the AXO-Lenti-PD Administration System.

The Sponsor will discuss all available safety data pertaining to the event with the reporting principal investigator. The Sponsor will also schedule an ad-hoc meeting with the SMC to discuss from a safety perspective, any potential impact of the benefit risk ratio of AXO-Lenti-PD and safety impact to subjects. If the study is suspended because of one of the above-listed events, this must be reported as soon as possible per regulatory guidelines as an Urgent Safety Measure (see Section 15.6). SMC and regulatory approval is required for resumption of the study after such an event. Where applicable, regulatory authorities and IRB/IECs will be notified of any actions taken with the study.

Any patients who have already received IMP and are currently in the study at the time study stopping criteria are met will continue to be followed-up, for safety and any signs of efficacy, per protocol.

8.4.9 Concomitant Medication or Treatment (including any surgical procedures)

All prescription and over the counter medications (including herbal remedies) taken by the patient for 28 days prior to administration of study therapy on Day 1 through to Month 12 will be recorded on the source document and eCRF. As far as possible, concomitant medications taken by the patients during the 15 year follow up will also be recorded on the source documents/eCRF. The Investigator may prescribe additional medications during the study, as long as the prescribed medication is not prohibited by the protocol. In the event of an emergency, any required medications may be prescribed without prior approval, but the Medical Monitor or designee must be notified of the use of any contraindicated medications

immediately thereafter. Any concomitant medications added or discontinued during the study will be recorded on the eCRF, including the start and end dates, as applicable.

Concurrent supportive care, including routine treatment for PD will not be restricted, including the use of narcotics for pain control and anti-emetics (corticosteroids and domperidone, should be used in preference to metoclopramide or prochlorperazine wherever possible due to these anti-emetics having anti-dopaminergic properties) for control of nausea. Special efforts will be made not to routinely prescribe other anti-emetics. Therapies considered necessary for the patient's well-being will be administered at the discretion of the investigator. Patients will not be prescribed tolcapone unless there are no other treatment options available, due to the potential to modify dopaminergic pathways within the brain. The administration of therapies to treat depression will be limited to those that target the serotonergic system. Those acting on the dopaminergic pathways will be avoided.

8.4.9.1 Concomitant Treatment with Parkinson's disease therapies and/or surgical procedures

At all study visits the patients' L-DOPA and additional PD therapy, including any surgical procedures, will be reviewed and changes documented in the source notes and eCRF (to account for minor fluctuations in doses of medication, per standard clinical care, a narrow range of dosing will be permitted in the eCRF). The patients' PD medication regimen should not be adjusted by the Investigator or patient unless an AE develops or clinically necessary. If it is determined by the site investigator that PD medications must be lowered, preference should be made for lowering non-levodopa PD medications, such as dopamine agonists. Patients should follow standard clinical care for managing their PD medication. LCIG, subcutaneous pumps, continuous patches should not be prescribed, and Apomorphine should not be prescribed as rescue medication during the first 12 months of the study although daily apomorphine therapy, which started prior to screening, is acceptable so long as the dose is kept constant. If a patient is prescribed amantadine (Mantadix) prior to enrolment into the study to reduce dyskinesias the patient should remain on the pharmacological therapy post-surgery. If a patient is not prescribed amantadine prior to inclusion in the study then it should not be administered following AXO-Lenti-PD administration to treat dyskinesias until modifications in L-DOPA and or dopamine therapy have been attempted and failed. If a patient's dyskinesias remain too severe following amendments to their dopaminergic therapy discussions will be held between the clinicians involved in the study prior to the potential addition of amantadine to the treatment regime.

Concomitant surgeries or procedures for PD such as DBS or the fitting of an apomorphine pump will be recorded on the eCRF and hospitalisation for these procedures or any follow-up maintenance of such devices will not be considered an SAE.

8.4.9.2 Withdrawal of PD Medication for “OFF”/“ON” Clinical and PET Assessments

Patients should be instructed to take their last dose of long acting dopamine agonists 2 days prior to the day that the “OFF”/”ON” assessments will be made. Patients will be advised to come to the hospital associated with the investigational site or PET centre the day prior to the “OFF”/”ON” assessments, and will take their last dose of L-DOPA early in the evening. Precise times will be advised to the patient by the team at the investigational site or PET centre based on the PD medications they are taking but the last L-DOPA dose must be at least 12 hours before the “OFF” assessment.

8.4.9.3 Dose of L-DOPA Challenge for “ON” assessments

For the “ON” assessments, patients will be given a challenge dose of L-DOPA sufficient to achieve an effective “ON” response, which will usually be between 100% and 150% of their usual morning dose; the normal morning dose of COMT inhibitor or agonist may be given simultaneously. The “ON” assessments will be recorded once a good “ON” response has been reached, as judged by patient and examiner. Timing will be flexible, but at least an hour after administration.

If the patient’s requirement for L-DOPA therapy decreases during the study, the investigator may adjust the L-DOPA challenge dose accordingly.

8.4.9.4 Dose of L-DOPA for Area Under the Curve (AUC) assessments (at participating sites)

At Cohort 3 and Part B patient visits that include evaluation of UPDRS in “OFF” state within the first year (Screening, Months 3, 6, 12) and in conjunction with the L-DOPA challenge for “ON” assessments, a sequence of UPDRS Part 3 motor examinations will be repeated at timed intervals to produce data for an exploratory “Area Under the Curve” analysis at participating sites. Beginning with the UPDRS Part 3 “OFF” score, the UPDRS Part 3 will be repeated at 30-minute intervals after the L-DOPA challenge to 2 hours, then hourly to 4 hours (0.5, 1, 1.5, 2, 3, 4 hours after L-DOPA challenge – i.e., within one post L-DOPA dose timeframe; if the next dose needs to be taken before the 4 hours elapsed, the final AUC UPDRS 3 should be performed just prior to the next dose, if at least 30 minutes passed from previous AUC UPDRS 3) and scores recorded. Other scheduled assessments and questionnaires can be completed during this period

9 STUDY PROCEDURES

9.1 Schedule of Study Procedures

The investigator must notify the sponsor or designee of deviations from protocol visits or evaluations and these evaluations, if applicable, must be rescheduled or performed at the nearest possible time to the original schedule. Protocol deviations will be recorded on the source document with an explanation for the deviation and also on the eCRFs. The investigator must comply with the applicable requirements related to the reporting of protocol deviations to the IRB/IEC. Details of pre-defined major and minor protocol deviations will be provided to sites prior to study start.

Patients/legal representatives will be instructed to call study personnel to report any abnormalities during the intervals between study visits and to come to the study site if medical evaluation is needed and the urgency of the situation permits. For emergency and other unscheduled visits to a medical facility other than the study site, medical records will be obtained by the investigator where possible and made available to the sponsor or designee during monitoring visits.

A schedule of study procedures is presented in Section 3, further details are provided below. Generally, recording of AEs should occur first at clinic visits. For the multiple questionnaires/scales (excluding the “OFF”/“ON” tests), it is recommended that cognitive tests should be performed first, and whatever order the tests are conducted at baseline should as far as possible remain the same at subsequent visits. Also, as far as possible, the raters for the tests administered to the patients should remain the same.

9.1.1 Informed Consent and Screening

All screening procedures must be performed within 60 days (plus a 7-day visit window) of administration of study treatment/ISP (Day 1) unless otherwise specified. Prior to screening patients, participating sites should check the safety and recruitment notifications from Axovant Sciences as necessary to ensure that patients can be scheduled for screening/surgery in accordance with the dose escalation recruitment criteria. Screening assessments will be considered as the baseline values, and where the same procedure is performed twice as a result of re-screening, the second reading will count as the baseline value. **The screening evaluations may be carried out over more than one visit** and will involve the patient being hospitalized on two occasions for the “OFF”/ “ON” assessments (the “OFF” and optional “ON” medication PET scans, and the “OFF”/ “ON” medication clinical assessments, including concordance testing).

1) Obtain written informed consent and any locally required authorization prior to any study-specific procedures

2) Register patient in screening via the eCRF (Part A patients) or in IxRS (Part B patients), ensure written authorisation is received from Axovant Sciences and IMP shipment scheduled.

3) Record full medical history, including PD history, prior treatments, current PD symptoms and demographics (year of birth, reproductive status, sex, race (if permitted in that country)

4) Perform a full physical examination

5) Collect vital signs (systolic and diastolic blood pressure, lying down and standing up, heart rate, and temperature), record height and weight

6) Collect samples for clinical laboratory tests (haematology, serum chemistry, coagulation and dipstick urinalysis)

7) Collect blood for Immunology and Biodistribution (qPCR), also urine for Biodistribution

8) Collect optional blood sample for PD-related research, including but not limited to genomic analysis

9) Screen for dementia using DRS-2

10) Assess suicidality using the C-SSRS “baseline” version

11) Evaluate nature of PD by:

   a) Rush dyskinesia rating scale before L-DOPA medication withdrawn for “OFF” assessments

   b) **“OFF” Medication Assessments** (before the first morning dose of L-DOPA and at least 12 hours after the last administration of L-DOPA the previous day – hospitalisation required; see Section 8.4.9.2)

      i) UPDRS Part 3 (to include optional video recording of Part 3)

      ii) Hoehn and Yahr

      iii) Rush dyskinesia rating scale (to include optional video recording of patient)

   c) **“ON” Medication Assessments** (see Section 8.4.9.3)

      i) At 30 minutes after L-DOPA challenge (at selected sites):

         (1) UPDRS Part 3 assessment for AUC analysis

      ii) At one-hour after L-DOPA challenge:

         (1) UPDRS Parts 1, 2, 3, 4 (to include optional video recording of Part 3)

         (2) Hoehn and Yahr

         (3) Rush dyskinesia rating scale (to include optional video recording of patient)

      iii) At least one hour after L-DOPA challenge (at selected sites):

(1) Sequential UPDRS Part 3 assessments for AUC analysis (1.5, 2.0, 3.0, 4.0 hours)

(For sites participating in AUC analysis, the below assessments may be staggered with the sequential UPDRS 3)

d) Neurological examination

e) Cognition assessment (MoCA)

f) Quality of Life assessments (PDQ-39 and EQ-5D-5L)

g) Depression/mood assessment (HADS)

h) Impulsivity assessment (QUIP-RS)

i) Starkstein Apathy Scale

j) CGI

k) PGI

l) NMSS

12) ECG

13) Chest X-ray

14) Hauser patient diary training and concordance testing

15) Baseline Hauser patient diary for confirmation of eligibility criteria (Experiencing at least 2.5 hours of “OFF” time confirmed by the Hauser patient diary for each of 3 consecutive days anytime during Screening)

16) A structural brain MRI to be used in eligibility assessment and the analysis of the PET data

17) Baseline [^{11}C]-(+)-PHNO PET scan in the “OFF” medication state and additional optional [^{11}C]-(+)-PHNO PET scan in the “ON” medication state (Note: the imaging will be conducted separately from the other “OFF” assessments and be done at a different facility). Results of PET scans do not affect patients’ eligibility for the main study. For women of childbearing potential participating in the PET sub-study, urine pregnancy test will be required at the PET centre.

18) During Screening Days -60 to -1, for women of childbearing potential not in the PET sub-study, one urine pregnancy test will be performed at the clinical site.

19) Record any AEs occurring after signing of the informed consent

20) Record all concomitant medications or treatments (including herbal therapies) that the patient has received within 28 days prior to surgery on Day 1

21) Anaesthesia assessment (time point per institution practice)

22) Verify eligibility criteria to ensure patient is eligible for the study.

The following assessments must be repeated on Day -1 (patient must be hospitalized):

1) Record any AEs observed or reported since the previous visit
2) Collect vital signs (systolic and diastolic blood pressure, lying down and standing up, heart rate, and temperature), record weight
3) Collect samples for clinical laboratory tests (haematology, serum chemistry, coagulation and dipstick urinalysis)
4) Focused physical examination at the discretion of the Investigator based on the patient's clinical condition (Physical exam must be performed on day -1 AND/OR day of surgery, but cannot be excluded on BOTH day -1 AND day of surgery)
5) Neurological examination
6) Anaesthesia assessment (time point per institution practice)
7) Pre-operative brain MRI as necessary (for sites unable to image on day of surgery)
8) Record any changes to the patient's concomitant medications or treatments (including herbal therapies) since the previous visit
9) Re-verify eligibility criteria to ensure patient is eligible for the study and if still eligible enrol patient in the study via the eCRF and confirm with Axovant Sciences there is no reason not to proceed to surgery (Part A patients) or IxRS (Part B patients) and inform Pharmacy of surgery timings
10) Urine pregnancy test

The patient's will also be prepared for surgery in accordance with the Surgical Manual which will include patient's being nil by mouth (except medication) 12 hours prior to surgery. Patients should be instructed as per local surgical guidelines to abstain from antiplatelet medication (i.e., aspirin) and other medications contraindicated prior to surgery.

9.1.2 Treatment Period

9.1.2.1 Day 1: Day of administration of AXO-Lenti-PD/ISP

Prior to surgery:

1) Physical examination (can be focused at the discretion of the Investigator based on the patient's clinical condition. Physical exam must be performed on day -1 AND/OR day of surgery, but cannot be excluded on BOTH day -1 AND day of surgery)
2) Collect vital signs (systolic and diastolic blood pressure, lying down and standing up, heart rate and temperature)
3) Administration of general anaesthetic, prior to attachment of stereotactic head frame

4) Brain MRI or CT scan (for sites conducting MRI prior to Day 1) to enable computation of three dimensional anatomical localisation of the putamen

Surgery and post-surgery on Day 1:

5) Administer IMP/ISP as detailed in the study Surgical Manual and Section 10.5.

6) Perform post-operative observations in accordance with standard hospital policy

7) Collect vital signs (systolic and diastolic blood pressure, lying down only, heart rate and temperature

8) Perform limited neurological examination

9) Post-operative brain MRI to assess surgical artefacts and confirm target localization (should preferably be taken within 3 hours of surgery completion, but no longer than 24 hours after).

10) Record any AEs observed or reported during Day 1

11) Record all new and any changes to concomitant medications or treatments (including herbal therapies) given to the patient during Day 1

9.1.3 Post-Surgery Follow-up Period

9.1.3.1 Follow-up on Day 2 and Day 3 post-surgery

1) Record any AEs observed or reported since the previous time point

2) Collect vital signs (systolic and diastolic blood pressure, lying down and standing up, heart rate and temperature)

3) Perform limited neurological examination

4) Physical examination (can be focused at the discretion of the Investigator based on the patient's clinical condition)

5) Collect samples for clinical laboratory tests (haematology, serum chemistry, coagulation and dipstick urinalysis) at Day 3 only post-surgery

6) Collect blood and urine samples for Biodistribution (qPCR)

7) Record any changes to the patient's concomitant medications or treatments (including herbal therapies) since the previous time point

9.1.3.2 Follow-up at Days 4, 8 and 15 post-surgery

1) Record any AEs observed or reported since the previous time point

2) Collect vital signs (systolic and diastolic blood pressure, lying down and standing up, heart rate and temperature); record weight (Day 15 only)

3) Neurological examination
4) Physical examination (can be focused at the discretion of the Investigator based on the patient's clinical condition)
5) Collect blood for clinical laboratory tests (haematology, serum chemistry, coagulation) (Day 8 only)
6) Collect blood and urine samples for Biodistribution (qPCR) (Day 8 only)
7) Collect blood sample for Immunology (Day 15 only)
8) Record any changes to the patient's concomitant medications or treatments (including herbal therapies) since the previous time point
9) On Day 15 visit, provide Hauser patient diary to patient, to complete assessments at home the week prior to the next clinic visit at 1 Month

9.1.3.3 Follow-up at Months 1 and 9 post-surgery (no "OFF" assessments and no hospitalization)

1) Record any AEs observed or reported since the previous visit
2) Collect vital signs (systolic and diastolic blood pressure, lying down and standing up, heart rate and temperature); record weight
3) Physical examination (can be focused at the discretion of the Investigator based on the patient's clinical condition)
4) Collect blood for clinical laboratory tests (haematology, serum chemistry, coagulation)
5) Collect blood and urine samples for Biodistribution (qPCR)
6) Collect blood sample for Immunology
7) Brain MRI scan (Month 1 only)
8) Evaluate nature of PD by:
    a) Neurological examination
    b) Cognition assessment (MoCA)
    c) PDQ-39 and EQ-5D-5L
    d) Depression/mood assessment (HADS)
    e) Impulsivity assessment (QUIP-RS)
    f) Starkstein Apathy Scale
    g) CGI
    h) PGI

i) Hauser patient diary (recorded for any 3 consecutive days the week prior to the clinic visit)

j) **“ON” Medication Assessments** (at least one hour after a dose of L-DOPA)

   i) UPDRS

   ii) Hoehn and Yahr

   iii) Rush dyskinesia rating scale

9) Record any changes to the patient’s concomitant medications or treatments (including herbal therapies) since the previous visit

10) Provide Hauser patient diary to complete assessment at home the week prior to next clinic visit, and bring to next clinic (diary can also be mailed to patient in time for completion the week prior to each visit)

11) Assess suicidality using the C-SSRS “since last visit” version

12) Urine Drug Screen

9.1.3.4 Follow-up at Months 3, 6 and 12 post-surgery (“OFF” and “ON” assessments with hospitalization)

Provide Hauser patient diary to patient approximately 1 month prior to visit, to complete assessments at home the week prior to the clinic visit and bring to next clinic visit as necessary.

Patient should come to the hospital associated with the investigational site or PET centre the night before these follow up visits (and for PET at Month 6 and optionally at Month 12) to allow for their PD medication to be withdrawn and the “OFF” assessments performed the following morning.

1) Record any AEs observed or reported since the previous visit

2) Collect vital signs (systolic and diastolic blood pressure, lying down and standing up, heart rate and temperature); record weight

3) Physical examination (can be focused at the discretion of the Investigator based on the patient’s clinical condition except at Month 6)

4) Collect samples for clinical laboratory tests (haematology, serum chemistry, coagulation at each visit, urine dipstick analysis at Months 6 and 12 only)

5) Collect blood and urine samples for Biodistribution (qPCR)

6) Collect blood sample for Immunology

7) Evaluate nature of PD by:

   a) Neurological examination

   b) Cognition assessment (MoCA)

c) PDQ-39 and EQ-5D-5L

d) Depression/mood assessment (HADS)

e) Impulsivity assessment (QUIP-RS)

f) Starkstein Apathy Scale

g) CGI

h) Dementia (DRS-2) at 6 months only

i) PGI

j) NMSS

k) Hauser patient diary (recorded for any 3 consecutive days the week prior to the clinic visit)

l) Rush dyskinesia rating scale before L-DOPA medication withdrawn for "OFF" assessments

m) **"OFF" Medication Assessments** (before the first morning dose of L-DOPA and at least 12 hours after the last administration of L-DOPA the previous day)

   i) UPDRS Part 3 (to include optional video recording of Part 3)

   ii) Hoehn and Yahr

   iii) Rush dyskinesia rating scale (to include optional video recording of patient)

n) **"ON" Medication Assessments** (see Section 8.4.9.3)

   i) At 30 minutes after L-DOPA challenge (at selected sites):

      (1) UPDRS Part 3 assessment for AUC analysis

   ii) At one-hour after L-DOPA challenge:

      (1) UPDRS Parts 1, 2, 3, 4 (to include optional video recording of Part 3)

      (2) Hoehn and Yahr

      (3) Rush dyskinesia rating scale (to include optional video recording of patient)

   iii) At least one hour after L-DOPA challenge (at selected sites):

      (1) Sequential UPDRS Part 3 assessments for AUC analysis (1.5, 2.0, 3.0, 4.0 hours)

         - (For sites participating in AUC analysis, questionnaires can be staggered with the sequential UPDRS 3)

8) ECG

9) Brain MRI scan (Months 6 and 12 only)

10) [^{11}C]-(+)-PHNO PET scan in the “OFF” medication state (Month 6, optionally at Month 12); additional optional [^{11}C]-(+)-PHNO PET scans in the “ON” medication state (Month 6 and 12) (Note: the imaging will be conducted separately from the other “OFF” state assessments and be done at a different facility). For women of childbearing potential participating in the PET sub-study, urine pregnancy test will be required at the PET centre.

11) Record any changes to the patient’s concomitant medications or treatments (including herbal therapies) since the previous visit

12) Assess suicidality using the C-SSRS “since last visit” version

9.1.3.5 Follow-up at Month 18 and Year 4 (“ON” assessments with no hospitalization)

Provide Hauser patient diary to patient approximately 1 month prior to visit, to complete assessments at home within 2 weeks prior to the clinic visit and bring to next clinic visit.

1) Record any AEs observed or reported since the previous visit
2) Collect vital signs (systolic and diastolic blood pressure, lying down and standing up, heart rate and temperature); record weight
3) Collect blood and urine samples for Biodistribution (qPCR)
4) Collect blood sample for Immunology
5) Physical examination (can be focused at the discretion of the Investigator based on the patient’s clinical condition)
6) Evaluate nature of PD by:
   a) Neurological examination
   b) Cognition assessment (MoCA)
   c) PDQ-39 and EQ-5D-5L
   d) Depression/mood assessment (HADS)
   e) Impulsivity assessment (QUIP-RS)
   f) Starkstein Apathy Scale
   g) CGI
   h) PGI
   i) NMSS
   j) Hauser patient diary (recorded for any 3 consecutive days within 2 weeks prior to the clinic visit)

k) **"ON" Medication Assessments** (at least one hour after a dose of L-DOPA)

i) UPDRS

ii) Hoehn and Yahr

iii) Rush dyskinesia rating scale

7) Record any changes to the patient's concomitant medications or treatments (including herbal therapies) since the previous visit

8) Assess suicidality using the C-SSRS "since last visit" version

9.1.3.6 Follow-up at Years 2, 3 and 5 ("OFF" and "ON" assessments with hospitalization)

Provide Hauser patient diary to patient approximately 1 month prior to visit, to complete assessments at home within 2 weeks prior to the clinic visit and bring to next clinic visit.

Patient should be hospitalized the night before these follow up visits to allow for their PD medication to be withdrawn and the "OFF" assessments performed the following morning.

1) Record any AEs observed or reported since the previous visit

2) Collect vital signs (systolic and diastolic blood pressure, lying down and standing up, heart rate and temperature); record weight

3) Collect blood and urine samples for Biodistribution (qPCR)

4) Collect blood sample for Immunology

5) Physical examination (can be focused at the discretion of the Investigator based on the patient's clinical condition)

6) Evaluate nature of PD by:

a) Neurological examination

b) Cognition assessment (MoCA)

c) PDQ-39 and EQ-5D-5L

d) Depression/mood assessment (HADS)

e) Impulsivity assessment (QUIP-RS)

f) Starkstein Apathy Scale

g) CGI

h) PGI

i) NMSS

j) Hauser patient diary (recorded for any 3 consecutive days within 2 weeks prior to the clinic visit)

k) Rush dyskinesia rating scale before L-DOPA medication withdrawn for "OFF" assessments

l) **"OFF" Medication Assessments** (before the first morning dose of L-DOPA and at least 12 hours after the last administration of L-DOPA the previous day)

   i) UPDRS Part 3

   ii) Hoehn and Yahr

   iii) Rush dyskinesia rating scale

m) **"ON" Medication Assessments** (at least one hour after a dose of L-DOPA)

   i) UPDRS Parts 1, 2, 3, 4

   ii) Hoehn and Yahr

   iii) Rush dyskinesia rating scale

7) Record any changes to the patient's concomitant medications or treatments (including herbal therapies) since the previous visit

8) Assess suicidality using the C-SSRS "since last visit" version

9.1.3.7 Follow-up at Years 6 - 9 and 11-14 (via phone call and sending questionnaire to patient only)

Provide Hauser patient diary to patient approximately 1 month prior to visit, to complete assessments at home and bring to a non study clinic visit (or give details to post back).

1) Record any AEs observed or reported since the previous visit

2) Evaluate nature of PD by:

   a) PDQ-39 and EQ-5D-5L

   b) Hauser patient diary (recorded for any 3 consecutive days within a month prior to or after the call)

3) Record any changes to the patient's concomitant medications or treatments (including herbal therapies) since the previous visit

4) Assess suicidality using the C-SSRS "since last visit" version

5) At end of study visit year 15, patients to be reminded about annual follow-up and their standard of care

9.1.3.8 Follow-up at Years 10 and 15

Where possible patients will be encouraged to attend the final follow-up assessment at year 10 and 15 in the clinic (if unable/unwilling to attend, an assessment can be done via the phone call in accordance with the section above). Prior to the visit the Hauser patient diary should be provided to the patient for completion before the visit.

1) Record any AEs observed or reported since the previous visit
2) Evaluate nature of PD by:
    a) Neurological examination
    b) Cognition assessment (MoCA)
    c) PDQ-39 and EQ-5D-5L
    d) Depression/mood assessment (HADS)
    e) Impulsivity assessment (QUIP-RS)
    f) Starkstein Apathy Scale
    g) CGI
    h) PGI
    i) NMSS
    j) Hauser patient diary (recorded for 3 consecutive days within a month prior to the clinic visit)
    k) **"ON" Medication Assessments** (at least one hour after a dose of L-DOPA)
        i) UPDRS
        ii) Hoehn and Yahr
        iii) Rush dyskinesia rating scale
3) Record any changes to the patient's concomitant medications or treatments (including herbal therapies) since the previous visit
4) Assess suicidality using the C-SSRS "since last visit" version

9.2 Details of Specific Procedures

9.2.1 Clinical Laboratory Tests

Clinical laboratory safety tests will be performed in the clinical laboratories of each investigational site, whilst urine dipstick may be performed at the clinical site. The following clinical laboratory tests will be performed (see section 3 for the schedule of tests):

**Serum Chemistry (approximately 5 – 10 mL of blood)**	
Calcium	Blood urea nitrogen (BUN)
Chloride	Uric acid
Phosphorus	Creatinine
Potassium	Total bilirubin (TBIL)
Sodium	Lactate dehydrogenase (LDH)
Aspartate transaminase (AST)	Albumin
Alanine transaminase (ALT)	Total protein (TP)
Alkaline phosphatase (AP)	Creatine phosphokinase (CPK)
Glucose (fasting glucose only at screening and Month 6)	Total cholesterol (CHOL) (only at screening and Month 6)
Glycosylated Haemoglobin Test (Haemoglobin A1c) (only at screening and Month 6)	Low-density lipoprotein cholesterol (LDL) (only at screening and Month 6)
Gamma glutamyl transferase (GGT)	High-density lipoprotein cholesterol (HDL) (only at screening and Month 6)
C-Reactive protein (CRP) (only at screening and Month 6)	Triglycerides (only at screening and Month 6)
Thyroid stimulating hormone (TSH) (only at screening and Month 6)	
**Haematology (approximately 5 mL of blood)**	
White blood cell (WBC) count with differential (neutrophils, lymphocytes, monocytes, eosinophils)	Platelet count
Red blood cell (RBC) count	Mean corpuscular volume (MCV)

Haematocrit (HCT)	Mean corpuscular haemoglobin (MCH)
Haemoglobin (HGB)	Red cell distribution width (RDW)
Erythrocyte sedimentation rate (ESR)	
**Coagulation (approximately 5 mL of blood)**	
Prothrombin time (PT)	Partial thromboplastin time (PTT)
**Dipstick Urinalysis* (approximately 20 mL of urine)**	
pH	Ketones
Protein	Bilirubin
Glucose	Leucocyte esterase
Blood	Nitrite
*Microscopy only if clinically indicated	
**Urine human chorionic gonadotropin (hCG) pregnancy test (for women of childbearing potential at Screening, Day -1 and Month 3 Visits)**	

9.2.2 Samples for Biodistribution, Immunology, Biomarkers and Pharmacogenomics

Further to the summary details below, a Laboratory Manual will be provided to all relevant sites (e.g. investigational sites, analytical laboratories, CROs) that specifies the procedures and instructions for collection (including volume of samples), processing, storage, and shipment of these samples, as well as laboratory contact information.

9.2.2.1 Samples for Biodistribution / qPCR

Blood and urine samples (5 mL required of each) will be taken according to the study schedule and analysed by qPCR to assess for the presence of AXO-Lenti-PD/vector-derived RNA or DNA.

In addition, samples may be collected if any patient experiences an adverse event of special interest (AESI; as defined in Section 11.7). If death occurs, additional biopsy samples may be provided by autopsy for further biodistribution studies (per Section 11.12).

Samples will be labelled with the patient number, date and time of collection, study visit and type of sample. They will be stored in a freezer at ≤-70°C prior to being shipped to OXB (contracted by Axovant Sciences) or another CRO with validated method on dry ice. They will

be kept for the duration of the study, in case any re-testing is required, for example for safety reasons.

9.2.2.2 Samples for Immunology

Blood samples (5–7 mL) will be collected in order to monitor any humoral immune responses against AXO-Lenti-PD and will be assessed by ELISA and/or Western blot analysis at the time points specified in the study schedule.

In addition, samples may be collected if any patient experiences an AESI (as defined in Section 11.7). If death occurs, biopsy samples may be provided by autopsy for immunological analyses investigations (per Section 11.12).

Samples will be labelled with the patient number, date and time of collection, study visit and type of sample. They will be stored in a freezer at ≤-70°C prior to being shipped to OXB (contracted by Axovant Sciences) or another CRO with validated method on dry ice. They will be kept for the duration of the study, in case any re-testing is required, for example for safety reasons.

9.2.2.3 Samples for Biomarkers and Pharmacogenomics

An optional blood sample will be collected for each patient at screening for DNA extraction. The sample will be assessed for biomarkers, to study the expression profile of genes known or suspected to be involved with PD, including but not limited to *ATP13A2, GBA, LRRK2, PARK2, PARK7, PINK1, SNCA, UCHL1, VPS35, TH, DDC, GCH1 and COMT*. The samples will be kept for the duration of the study, in case a baseline sample needs to be re-checked, or if additional genes are found to be associated with PD. The results will be used to see if there is a correlation between any clinical improvements and the particular genotype of a patient, and also to compare the genetic variants observed in the patients in this study to published research.

Samples will be labelled with the patient number, date and time of collection, study visit and type of sample. They will be stored in a freezer at ≤-70°C. Shipping details will be provided in the Laboratory Manual.

9.2.3 Physical Examination, Vital Signs and Other Observations related to Safety

A full physical examination is to be conducted at screening and at Month 6, but at other visits this can be limited to a focused physical examination at the discretion of the Investigator based on the patient's clinical condition and what is appropriate to be assessed. A full or focused physical exam must be performed on day -1 AND/OR day of surgery, but cannot be excluded on BOTH day -1 AND day of surgery.

Baseline physical examination will consist of the following body systems: (1) eyes; (2) ears, nose, throat; (3) cardiovascular system; (4) respiratory system; (5) gastrointestinal system; (6)

dermatologic system; (7) extremities; (8) musculoskeletal system; (9) nervous system; (10) lymph nodes; (11) other.

Vital signs including systolic and diastolic blood pressure, heart rate and temperature will be assessed at screening and subsequent visits per the study schedule. Blood pressure readings will be assessed with the patient lying down and standing up, apart from in the 24 hours post-surgery where only lying down blood pressure readings will be required. Any clinically significant change in vital signs will be recorded as an AE.

Height and weight will be recorded at screening, and weight only will be recorded at subsequent visits per the study schedule.

A standard 12-lead ECG will be performed during screening and Months 3, 6 and 12, and any new or worsening clinically significant abnormalities post-surgery recorded as an AE (screening abnormalities to be recorded as a pre-treatment AE). For safety monitoring purposes, the investigator or designee must review, sign and date all ECG tracings. Paper copies will be kept as part of the patient's permanent source documents at the site.

A chest X-ray will be performed during screening, and any clinically significant abnormalities recorded on the medical history.

9.2.4 Hauser Patient Diary Training, Completion and Concordance Testing

Patients will be requested to complete self-reporting diaries[37] detailing their motor function, indicating on a half-hourly basis over a 3-day consecutive period what functional state they are experiencing (selecting from 5 options; asleep, "OFF" state, "ON" state without dyskinesia, "ON" state with non-troublesome dyskinesia and "ON" state with troublesome dyskinesia). During screening, concordance testing will include educating the patient on these motor states, and evaluating the reliability with which a patient is able to correctly identify their motor state as compared to the rater. A concordance accuracy of ≥75% must be achieved between 2.5 hours and 6 hours of testing. The diary should be completed on 3 consecutive "typical" days within the week prior to visits through Month 12, and within 2 weeks prior to subsequent study visits. Acceptable compliance is no more than 2 hours of missing data per day, on at least 2 of the 3 days. If a patient is not compliant with their 3-day home diary or misses significant data, they may be allowed to repeat the assessment.

9.2.5 Questionnaires

9.2.5.1 UPDRS Part 1, 2, 3 and 4

The UPDRS questionnaire[38] is used to grade and monitor the severity of motor aspects of PD in individual patients. It will be administered to the patients by structured interview and examination, per the study schedule. Part 3 shall be performed in both the "OFF" and "ON" states, while Parts 1, 2 and 4 shall be performed only in the "ON" state. The questionnaire should take approximately 20 minutes to complete. It should be administered by a certified

member of the investigational site team, and details of the training and certification will be provided to sites prior to study initiation. The UPDRS is in four parts with a total possible score of 199:

- Part 1: Mentation, behaviour, and mood (4 questions; total possible score of 16)
- Part 2: Activities of daily living (13 questions; total possible score of 52)
- Part 3: Activities of motor examination (14 questions; total possible score of 108)
- Part 4: Complications of therapy (11 questions; total possible score of 23)

There will be optional video recording of Part 3 in the “OFF” and “ON” states where applicable at baseline, Month 3, Month 6, and Month 12 for visual documentation of motor symptoms.

9.2.5.2 Dyskinesia rating scale

The Rush Dyskinesia Rating Scale[39] (RDRS) is an objective rating scale for dyskinesia assessment during activities of daily living. The rating will be performed by a member of the investigational site team experienced in the use of the scale; training will be provided to sites prior to study initiation if necessary. The rater should observe the patient putting on and buttoning a coat, taking a cup from a table top to their lips, and walking. From their observations, the rater will rate the severity of the dyskinesia, check which dyskinesias are observed, and check the type of dyskinesia that is causing the most disability on the specified tasks. This assessment should take approximately 15 minutes. In addition to the RDRS being undertaken in the “OFF” and “ON” states per the study schedule, it must also be performed before L-DOPA medication is withdrawn for the “OFF” assessments.

There will be optional video recording of the patient undergoing this assessment in the “OFF” and “ON” states where applicable at baseline, Month 3, Month 6, and Month 12 for visual documentation of dyskinesia.

9.2.5.3 Quality of Life: PDQ-39

The PDQ-39 questionnaire[40] is a self-administered patient questionnaire designed to address aspects of functioning and well-being for those affected by PD. The questionnaire has 39 questions incorporated into eight discrete scales (mobility, activities of daily living, emotional wellbeing, stigma, social support, cognition, communication and bodily discomfort). Patients are asked to think about their health and general well-being and to consider how often in the last month they have experienced certain events (e.g. difficulty walking 100 yards). Patients are asked to indicate the frequency of each event by selecting one of 5 options: never/occasionally/sometimes/often/always (or cannot do at all). It should take between 10 and 20 minutes to complete.

9.2.5.4 Quality of Life: EQ-5D-5L

The EQ-5D-5L[41] is a descriptive system of health-related quality of life states consisting of five dimensions (mobility, self-care, usual activities, pain/discomfort, anxiety/depression) each of which can take one of five responses. The responses record five levels of severity (no problems/slight problems/moderate problems/severe problems/extreme problems) within a particular EQ-5D dimension – the EQ-5D-5L descriptive system and the EQ visual Analogue scale (EQ VAS). The EQ VAS records the patient's self-rated health on a 20 cm vertical, visual analogue scale with endpoints labelled 'the best health you can imagine' and 'the worst health you can imagine'. The patients will be asked to complete the EQ-5D according to how it best describes their health on that day, and it should take 2 - 5 minutes to complete.

9.2.5.5 Depression/mood

The Hospital Anxiety and Depression Scale[42] (HADS) will be used. This is a self-administered questionnaire comprised of 14 questions which assess levels of depression and anxiety. It should take approximately 2 - 5 minutes to complete, and the patients will be asked to complete it according to how they have felt in the past week.

9.2.5.6 Cognition

The Montreal Cognitive Assessment[43] (MoCA) is a screening instrument for mild cognitive dysfunction. It assesses different cognitive domains: attention and concentration, executive functions, memory, language, visuoconstructional skills, conceptual thinking, calculations, and orientation. A member of the clinical team will be required to administer the MoCA, and it should take approximately 10 minutes to complete.

9.2.5.7 Impulsivity

The Questionnaire for Impulsive-Compulsive Disorders – Rating Scale in Parkinson's Disease (QUIP-RS)[44] is designed to assess impulse control disorders (ICDs) and other compulsive behaviours in PD. It has 3 sections: Section 1 assesses four ICDs (involving gambling, sexual, buying, and eating behaviours), Section 2 assesses other compulsive behaviours (such as hobbies, simple motor activities and walkabout), and Section 3, compulsive medication use. It is a self-administered questionnaire composed of 30 questions. It should take approximately 5 minutes to complete.

9.2.5.8 Dementia

The Mattis Dementia Rating Scale–2™ (DRS-2)[45] is a battery-style assessment of mental status in older patients, which will be used as a screening tool to exclude patients with dementia and to assess patients at 6 months for emerging signs of dementia. The DRS-2 consists of 24 brief subtests whose scores are combined into five domains (attention,

initiation/perseveration, construction, conceptualization, and memory). A member of the clinical team will be required to administer the questionnaire, and it should take approximately 15 – 30 minutes to complete.

9.2.5.9 Non-Motor Symptoms

The Non-Motor Symptom Scale (NMSS)[46] is a validated measure for measuring the burden of a wide range of nonmotor symptoms including nonmotor fluctuations. It has been verified to have acceptable clinimetric properties to capture in a single instrument a broad range of NMSs that occur commonly in PD. The NMSS has been shown to correlate the impact of NMS on health-related quality of life. The good reliability plus acceptable precision support its use as an outcome measure in clinical trials, and it has been used to monitor NMS in multiple such trials.

9.2.5.10 Apathy

The Starkstein Apathy Scale[47] will be used. This is a 14-item scale measuring cognitive, emotional, and behavioural symptoms of apathy. Items are rated on a 0 to 3 scale (“not at all”, “slightly”, “some” and “a lot”) with scores ranging from 0-42 (higher scores indicating more severe apathy). A member of the clinical team will be required to administer this scale and it should take approximately 5 - 10 minutes to complete.

9.2.6 Neurological Examination

This full neurological examination will include, but not limited to, assessments of the patient’s level of consciousness, speech, cranial nerves, muscle strength, coordination/cerebellar function, sensation and reflexes. Time points of assessment are per the study schedule. On post-surgery days 1, 2 and 3 a limited neurological examination can take place pending the patient’s condition and ability to conduct the examination.

9.2.7 L-DOPA and PD therapy

Review of L-DOPA and PD therapy will take place at the time points specified in the study schedule.

9.2.8 Imaging

9.2.8.1 Striatal dopaminergic changes assessed using PET

Only patients eligible for the main study and considered appropriate for the PET sub-study by the investigator will attend the PET centre associated with the investigational site for the conduct of the PET at the visits where PET is performed. Patients who are not able to tolerate PET-related procedures based on investigators’ judgement will not be required to participate

in the PET sub-study. For women of childbearing potential participating in the PET sub-study, urine pregnancy test will be required at the PET centre for each PET visit. Pregnant women will not be scanned. The PET centre will be at a different location from the investigational site and the PET visit will occur on a different day from the clinical visit. An MRI scan of the brain will be acquired during Screening in order to provide anatomical information required for the analysis of the PET data, and will last up to 30 minutes. If the MRI scan performed at the investigational site during Screening has acceptable quality, no additional MRI scan will be done at the PET centre. The PET scans will be performed with patients in the "OFF" medication state (see Section 8.4.9.2). Patients will be admitted overnight prior to the PET scan. Prior to the start of the PET scan, a venous cannula will be placed into the antecubital or forearm vein, patients will be positioned in the PET scanner, and a low dose CT scan of the head will be acquired to estimate tissue attenuation of the PET signal. Following the acquisition of the low dose CT scan, patients will receive an intravenous injection of $[^{11}C]$-(+)-PHNO as a slow bolus (over approximately 20 seconds). Emission PET data acquisition will start contemporaneously with the $[^{11}C]$-(+)-PHNO administration and data will be acquired for approximately 90 minutes, with the whole PET session lasting approximately 120 minutes. The injected dose of $[^{11}C]$-(+)-PHNO at each PET scan will not exceed 180 MBq, with the mass of (+)-PHNO injected not exceeding 30 ng/kg. Emission PET data acquired will be analysed in conjunction with the MRI derived anatomical data, using an appropriate kinetic model, to generate regional BP_{ND}, where the BP_{ND} is a parameter proportional to the availability of the D_2/D_3R binding sites. Two required PET scans in the "OFF" medication state will be performed at Screening, Month 6 and another optional scan may be performed at Month 12. Patients who do not undergo a PET scan at baseline will not be requested to have a scan at 6 or 12 months.

Additional optional $[^{11}C]$-(+)-PHNO PET scans, in the "ON" medication state using a fixed dose L-DOPA challenge (250 mg L-DOPA + 25 mg carbidopa), may also be performed at Screening, Month 6 and Month 12. Brief details are provided in Appendix 2, and full details regarding image acquisition and analysis will be provided in a separate Independent Imaging Manual and Imaging Report.

9.2.8.2 MRI / CT scanning at Investigational Sites

Patients will be scanned by MRI and/or CT at investigational sites per hospital procedures and the MRI Imaging Manual at the time points specified in the study schedule. Preoperative and postoperative MRI images, as well as MRI scans obtained for safety reasons, will be transferred to a central imaging database for Sponsor review.

9.2.9 Other Measures

9.2.9.1 Hoehn and Yahr (H&Y)

The Hoehn and Yahr scale[48] is used to categorise the degree of clinical disability in patients with PD (range: Stage One (Mild) to Stage Five (Cachectic)) by the clinical team. This scale will be recorded in the "OFF" and "ON" state per the study schedule, and should take approximately 2 minutes to complete.

9.2.9.2 Clinical Global Impression (CGI)

The Clinical Global Impression (CGI) scale[49] provides a brief, stand-alone assessment of the clinician's view of the patient's global functioning prior to and after initiating an intervention to their treatment. The CGI has two components - the CGI-Severity (CGI-S), which rates illness severity, and the CGI-Improvement, which rates change from the initiation (baseline) of the intervention. The CGI-S rating will be based upon observed and reported symptoms, behaviour, and function in the past seven days, and categorises patients on a seven-point scale from "Normal, not at all ill" to "Among the most extremely ill patients". For CGI-I, the clinician will compare the patient's overall clinical condition to the CGI-S score obtained at baseline. This is also scored on a seven-point scale, from "Very much improved" to "Very much worse". Following a clinical evaluation, the CGI form can be completed in approximately a minute by an experienced clinician.

9.2.9.3 Patient Global Impression (PGI)

The Patient Global Impression (PGI) scale[50] provides a brief, stand-alone assessment of the patient's view of their global functioning prior to and after initiating an intervention to their treatment. The PGI is a patient reported outcome (PRO) consisting of one item that evaluates all aspects of patients' health and assesses if there has been an improvement or decline in their clinical status.

9.2.9.4 Patient baseline characteristics

During screening, year of birth, height, weight, sex, reproductive status, race, inclusion/exclusion criteria, clinical symptoms of PD, medical history, PD treatment history, chest X-ray, ECG and anaesthesia assessment will be recorded.

9.2.9.5 Assessment of Suicidality

Subjects will be assessed for suicidality before and during the study using the Columbia Suicide Severity Rating Scale (C-SSRS). The "baseline" version of the C-SSRS that references subject lifetime history and past year should be administered at the Screening visit. The "since last visit" version of the C-SSRS should be administered at all subsequent visits.

Subjects considered to be at significant risk will be excluded from the study. The C-SSRS is a brief measure that is designed to assess severity and change of suicidality by integrating both behaviour and ideation. It assesses intensity of ideation (a potentially important marker of severity), specifically asking about frequency, duration, controllability, deterrents, and reasons for the ideation that was most severe during the respectively assessed timeframe. Suicidal behaviour is also assessed by asking further questions to categorize the behaviours into actual, interrupted, or aborted attempts, as well as preparatory and non-suicidal self-injurious behaviour. The C-SSRS will be completed by a rater who is trained and certified to administer this scale. Any change in C-SSRS score indicating the presence of suicidality should be evaluated by the investigator for clinical significance to determine continued study eligibility (Section 8.3.2) and appropriate clinical actions (including but not limited to a referral to a mental health professional).

Clinically meaningful suicidal ideation, suicidal behaviour, and completed suicide should be recorded as AEs or SAEs.

10 INVESTIGATIONAL MEDICINAL PRODUCT AND TREATMENT

10.1 AXO-Lenti-PD Supply and Labelling

AXO-Lenti-PD is classified as an ATMP and for the purpose of this study protocol referred to as an IMP.

AXO-Lenti-PD will be supplied to the site in 0.3 mL type I borosilicate glass 'V' vials with a stopper and aluminium crimp seal. Each vial is uniquely numbered and will be filled with 130 µL of AXO-Lenti-PD. To account for loss of vector during pooling, priming and dead space in the AXO-Lenti-PD Administration System, 12 vials are required per patient dose for dose levels 1 and 2; whilst 18 vials are required per patient dose for dose level 3.

Each vial (primary container) of AXO-Lenti-PD will be packaged into a secondary container with a foam insert. Both the primary and secondary containers will be labelled in accordance with annex 13 of Volume 4 of "The rules governing medicinal products in the European Union" and in Commission Directives 91/356/EEC, as amended by Directive 2003/94/EC, and 91/412/EEC. The primary container (vial) is a small container and therefore the expiry date of the product is specified only on the secondary containers.

Two dose strengths of AXO-Lenti-PD have been prepared for the proposed clinical trial: 4.4×10^6 TU/mL (Dose Level – Cohort 1), and 1.5×10^7 TU/mL (Dose Level – Cohort 2 and Cohort 3). Both concentrations were prepared by dilution of a more concentrated vector (at the time of manufacture) using tris, sucrose, sodium chloride and mannitol (TSSM) formulation buffer. The dose of each vial will be clearly identified on the primary and secondary container labels.

Mock drug vials will also be provided for the patients undergoing ISP in Part B of the study. These vials will be the same 0.3 mL type I borosilicate glass 'V' vials used for AXO-Lenti-PD,

however they will remain empty without the stopper or aluminium crimp seal. The mock drug (ISP) vials will be labelled stating that they are not for administration. Each vial will be issued in a secondary container identical to those used for AXO-Lenti-PD. The labelling of the secondary containers will also be identical to that of AXO-Lenti-PD secondary containers with the exception that they will be uniquely numbered. Depending on the optimal dose chosen to move to Part B of the study, the appropriate numbers of mock vials in their secondary containers are provided for a single patient dose similar to the AXO-Lenti-PD vials described above.

Vials will be shipped frozen on dry ice to the clinical sites once all relevant study approvals have been received in writing (and in accordance with Section 8.4.1 for Part A patients). The packaged vials will be distributed to the sites on a per patient basis with additional vials provided as backup for Part A. In Part B, both treatment options (to maintain blinding) will be provided to sites. Back up vials from both treatment options will be available should there be any damage or problem with the vials on inspection at sites and also as a precaution for any surgical procedural issues that may require additional vials to ensure the intended dose can be given.

Healthcare staff that are pregnant, or suspect they are, must not administer or handle AXO-Lenti-PD.

A Pharmacy Manual will also be provided to the sites providing detailed instructions on all aspects of the IMP management, labelling and preparation.

10.2 Storage and Dispensing of Study Medication

Storage and dispensing of study medication will be conducted in accordance with the Pharmacy Manual and the overview below.

AXO-Lenti-PD is a frozen liquid formulation, stored at -80˚C. AXO-Lenti-PD must be stored in a locked temperature-controlled freezer in the hospital pharmacy. The freezer must be monitored daily and any deviation in temperature from -70˚C to -90˚C reported to Axovant Sciences as soon as possible and prior to further clinical use of the IMP. The IMP must be stored in such a way that it cannot be mixed up or confused with any other medications.

The secondary cartons with empty ISP vials for Part B of the study will also be shipped to the sites on dry ice and stored in identical conditions, in a locked -80°C freezer in the hospital pharmacy.

The relevant cartons of IMP will be allocated when a patient is registered in the eCRF and Axovant Sciences authorisation form received for Part A patients and via the IxRS (see Section 8.4) for Part B patients.

Investigators and pharmacists should note that the clinical trial supplies may only be used for the clinical trial for which they are indicated. They must not be employed for any other clinical or nonclinical use.

10.3 IMP Accountability and Destruction

The investigator or designee (e.g. pharmacist) is responsible for maintaining accurate IMP accountability records for reconciliation and full traceability of all vials used in the study. The IMP accountability records for Part B must be maintained by designated unblinded staff and should only be reconciled by an unblinded monitor during the study (or the blinded monitor at the end of the study or when the blind is broken). The accountability should be able to trace each uniquely numbered vial to an individual patient.

The specific details of IMP supplies (date of receipt and dispensing, lot number, expiration date, dose preparation, accountability for each uniquely numbered vial and storage details) will be logged at the site. Per European Commission Guidelines on GCP specific to ATMPs[51], these documents along with other study documents showing the traceability of the IMP at the site will be maintained by the investigator for a minimum of 30 years after the expiration date of the product and will not be destroyed without formal written approval from the study sponsor.

All unused IMP will be returned to an Axovant Sciences-authorized depot or upon authorization by Axovant Sciences, disposed of in accordance with the hospital policy on genetically modified material (refer to the Pharmacy Manual or other written instructions provided by Axovant Sciences or its designee for contact information and specific shipping instructions).

10.4 Treatment Regimen

AXO-Lenti-PD will be administered by stereotactic infusion bilaterally to the posterior putamen of a patient on Day 1 of the study. For patients undergoing ISP in Part B of the study, the ISP will also be deemed Day 1. Full details of the treatment, surgical procedures (including pre and post-surgery details) and administration of AXO-Lenti-PD or ISP are included in the study Surgical Manual; an overview of which is presented below.

10.5 IMP Administration for Part A and Part B

10.5.1 Hospitalisation

Patients will be admitted to hospital the day before surgery, and will be nil by mouth (except medication as surgery will be performed in the “ON” state) for the 12 hours prior to surgery. Patients will have a shower with betadine red soap, their hair will also be carefully washed with betadine red soap on the night before and again on the morning of surgery (hair will be dried prior to surgery). Patients at sites where an MRI cannot be conducted on the day of surgery will have their pre-operative brain MRI scan on the day prior to surgery. Patients must avoid blood thinning medications including antiplatelets (such as aspirin) prior to procedure as per surgical protocol.

10.5.2 General Anaesthesia

On the morning of the surgery patients will be taken to the operating theatre and given general anaesthesia. Details surrounding the anaesthesia of an individual patient will have been discussed during the patient's anaesthesia assessment at screening.

General anaesthesia may be conducted according to national guidelines and practices and local hospital procedures; this is to include anaesthesia and patient monitoring during and after the surgery. Any change to the site procedures for general anaesthesia detailed in the Surgical Manual must be approved by the PI and Axovant Sciences.

10.5.3 Attachment of Stereotactic Head Frame

When under general anaesthesia, the stereotactic head frame (e.g. Leksell® stereotactic frame, Elekta® Sweden or equivalent) will be secured to the patient's head, parallel to the anterior commissure (AC)-posterior commissure (PC) line. The stereotactic head frame will be fastened to the patient's skull with four pins. The localising box will be attached to the base ring prior to the patient undergoing a MRI scan (or CT scan for those sites that cannot perform MRI on day of surgery). From this information it will be possible to compute a three dimensional anatomical localisation of the putamen.

10.5.4 Preparation of Surgical Site

Following stereotactic MRI or CT, the base ring of the stereotactic apparatus will either (per hospital practice) be attached to the operating table (thereby fixing it in position so that the patient does not have to support the weight of the equipment) or a vacuum sand-filled cushion will be used to immobilise and support the patient's head. The patient's head will be positioned with up to 30° flexion to reduce CSF leakage during the entire surgical procedure at the discretion of the investigator. A small area of the scalp may be shaved and the surgical incision site scrubbed with either a povidone-iodine scrub (e.g. Betadine®) or a chlorhexidine scrub. The patient's head will be covered with sterile surgical drapes. All surgery will be performed using universal and local aseptic procedures. The scalp will be incised at each entry point, retracted and two burr holes, one either side of the brain, made in the skull along the planned trajectory, using an electric or pneumatic Codman drill or equivalent. A minimal dura incision will be made to prevent CSF loss and air entry into the intracranial cavity during the procedure.

For patients randomised to Part B, see Section 10.6 below for the timing and preparation of the scalp.

10.5.5 AXO-Lenti-PD Preparation

Detailed instructions regarding IMP preparation, handling, labelling and destruction can be found in the Pharmacy and Surgical Manuals that will be provided to sites.

For Part B patients randomised to ISP see Section 10.6 below.

The IMP kit number (and/or vial numbers) for Part A will be allocated once a patient is registered in the eCRF and an Axovant Sciences authorisation form received; the surgical team will advise the pharmacy on timings for delivery of the IMP to the theatre for each patient. The pharmacist or delegate will remove the appropriate number of vials of AXO-Lenti-PD from the -80°C storage and inspect the secondary container for any damage. If the tamper-evident seal on the secondary container is broken, Axovant Sciences should be notified as soon as possible, the vial will not be used and will be retained in quarantine at -80°C by pharmacy for reconciliation.

The unique vial numbers and the date and time of removal from the -80°C storage will be documented in the pharmacy log. The vials, whilst sealed in the tamper-evident containers, should be transported to the operating theatre in an appropriate transportation box at room temperature. The time of vial delivery to the theatre must be recorded in the pharmacy log or surgical notes.

Once in the operating theatre, the glass vial should be removed, inspected and placed in a vial tray. If the contents of the vials are still frozen then they should be exposed to room temperature until thawed. Immediately prior to use, the appearance of the contents of the vial, unique vial number and details of the labelling should be recorded in the source records in addition to the time of administration.

If there is a need for additional low or medium doses, a high dose IMP can be diluted on site with buffer solution to create a low or medium dose IMP. The dilution procedure is fully described in the OXB-102-01 Pharmacy and Surgical Manuals.

10.5.6 Locating Sites for AXO-Lenti-PD Administration

The stereotactic MRI and CT images if applicable will be used with specialist computer software to locate both right and left putamen and the sites into which AXO-Lenti-PD will be injected. The first infusion will be targeted to the AC level of the putamen, followed by a second and third infusion approximately 6 mm and 12 mm caudal from the first. Only one deposit of vector will be made per cannula tract, at a depth 10 mm above the inferior limit of the putamen. The coordinates for all doses may vary between each putamen as individual size and shape will differ between patients. All coordinates for each infusion will be recorded in the source data and transcribed into the eCRF for Part A patients (for Part B patients this will be recorded in blinded notes).

10.5.7 AXO-Lenti-PD Administration System

AXO-Lenti-PD administration will be made using an infusion device (AXO-Lenti-PD Administration System) in conjunction with standard surgical equipment including the stereotactic frame. The AXO-Lenti-PD Administration System is an assembly of various medical devices which can accurately deliver the required small quantities of AXO-Lenti-PD

vector into the specific targets in the patient brain using precise flow rates. Full details can be found in the AXO-Lenti-PD Administration System device IB.

The AXO-Lenti-PD Administration System includes the following device components:

a. Medfusion® 3500 V6 syringe pump (for AXO-Lenti-PD administration at a precise volume and flow rate)
b. AXO-Lenti-PD Delivery Syringe (to be loaded into the pump):
    - Cohorts 1 and 2: 1mL Luer Lok™ BD™ syringe
    - Cohort 3: 3mL Luer Lok™ BD™ syringe
c. BD™ Luer Tip cap (to prevent leakage of syringe contents)
d. BD™ blunt fill needle (to withdraw AXO-Lenti-PD from the vials)
e. SmartFlow® Neuro Ventricular Cannula (to transfer the AXO-Lenti-PD into the brain)

Manual microTargeting™ STar™ Drive System (to support the cannula and to enable accurate targeting of the precise location in the brain; minimising the risk of blood vessel damage) The AXO-Lenti-PD Administration System should be assembled and used according to the Surgical Manual and the user instructions for all components which will be provided.

A total of 6 sequential infusions will be made via three tracts in the first hemisphere and three tracts in the second hemisphere. When required, the contents of 6 or 7 vials for the first hemisphere should be drawn into the syringe and any air bubbles expelled. A minimum of 600 µL (Cohorts 1 and 2) or 1,100 µL (Cohort 3) should be present in the syringe before attaching to the cannula which will be sufficient for three infusions (when using one cannula for each of the three infusions and accounting for priming and dead volume loss in the administration system). This will be repeated for the second hemisphere so a total of 12 vials per patient will be required for Cohorts 1 and 2, and 18 vials per patient for Cohort 3. Additional vials will be available at pharmacy as part of risk mitigation should there be any issue during the surgical procedure which may require, for example, the re-assembly of the AXO-Lenti-PD Administration System with a new cannula should more than one cannula be required for three infusions. Spare components of the AXO-Lenti-PD Administration System will also be available at site.

Depending on surgical preference, prior to inserting the cannula to the microTargeting™ STar™ Drive System, a stylet may first be inserted into the microTargeting™ STar™ Drive System and then removed; the purpose of this is to make a channel down the target trajectory and move any blood vessels out of the way around the cortical sulcus part of the trajectory.

The syringe and cannula will be attached to the syringe pump and then threaded through the microTargeting™ Star™ Drive System which is fixed to the stereotactic frame.

AXO-Lenti-PD will be administered vertically from the superior surface of the head, with the cannula being angled to avoid the ventricles, caudate nucleus and middle cerebral artery. Depending on the location of each putamen, it is expected that the cannula will be inserted

into the brain tissue to around a depth of 65 mm ± 5mm and the infusions will be administered with a flow rate of 3 µL/min (Cohorts 1 and 2) or 5 µL/min (Cohort 3).

For Cohorts 1 and 2, the total surgery time (including the preparation of the scalp, stereotactic frame attachment and MRI/CT) is estimated to be 7 to 8 hours (includes 3 to 4 hours for infusion). For Cohort 3, the total surgery time is approximately is estimated to be up to 10 to 12 hours (includes 6 to 8 hours for infusion). The Surgical Manual provides additional information including contingencies and risk mitigations for the surgical procedure including details on spare equipment and IMP.

On removal of the cannula from the final infusion track the bore holes will be sealed with Craniosorb AFS (Codman®, USA) (or equivalent) to avoid non aesthetic skin defects in the long term, and the scalp incision sutured. The scalp will then be swabbed with Betadine yellow solution prior to a dressing being applied. The AXO-Lenti-PD Administration System will be disassembled and the base ring of the stereotactic head frame removed from the patient's skull by unscrewing the four pins. The entry holes from the stereotactic frame are small and should not require suturing.

A post-surgical MRI to assess surgical artefacts and confirm target localization should preferably be taken within 3 hours of surgery completion, but no longer than 24 hours after.

For all dose levels, the start and finish time of each infusion will be recorded in the surgical notes and later transcribed into the eCRF for Part A patients. At the end of the procedure the single use components (in addition to used vials once fully accounted and documented) will be destroyed according to hospital policy, and a certificate of destruction will be issued and kept in the study file. All other re-useable equipment will be cleaned according to the Operators Manuals. In the event there are any unused vials remaining after the surgical procedure these should be returned to pharmacy in accordance with Section 10.3.

All disposable surgical supplies; including gloves, masks, gowns, dressings and swabs used during the surgical procedure will be destroyed by incineration according to hospital policy at the end of the operation. The first dressings removed from the patient's wounds following surgery will also be disposed of by incineration.

10.5.8 Post-Surgical Monitoring

The patient will be transferred to the recovery room for post-operative observations, until they are cognitively, neurologically and physically stable.

Sites should follow their specific hospital policies and procedures during the post-surgical monitoring process.

Any differences in surgical procedures between sites will be documented in the final CSR.

10.6 Differences in Procedures for Part B Patients.

For patients in Part B of the study, they will be admitted the day before surgery and on Day 1 undergo all preoperative procedures including anaesthesia, fixation of the stereotactic frame and MRI/CT scan per above.

The IMP kit number of the secondary cartons (see Section 10.2) will be allocated via the IxRS and the surgical team will advise the pharmacy on timings for delivery to theatre for each patient. The preparation of the surgical site should not commence until the vials have been received into theatre as this will determine whether the burr holes are drilled or just skin incisions made.

When the vials are received in the operating theatre the pharmacist will also provide the randomisation allocation in writing from the IxRS to the surgical team to confirm the correct vials have been transferred to theatre. The neurosurgeon and operating staff will then establish if the patient is randomised to undergo ISP (and therefore check empty vials labelled ‘not for administration’ are present) or AXO-Lenti-PD administration (and check that vials containing AXO-Lenti-PD are present).

If the vials contain AXO-Lenti-PD, then burr holes will be drilled into the skull and AXO-Lenti-PD administered as for Part A patients and the details in the preceding sections. If the vials are empty, then only bilateral skin incisions at locations guided by the neuronavigation system under strict aseptic conditions will be performed. To reduce the risk of infection, no holes will be drilled in the skull. As the burr holes will be sealed with Craniosorb AFS (Codman®, USA) (or equivalent) in patients who receive AXO-Lenti-PD, Craniosorb AFS (or equivalent) will be placed over the bone at the skin incision site during the ISP in order to ensure blinding is maintained during postoperative assessments including imaging. The incisions in the scalp will be closed and the patient removed from anaesthesia after a similar time to those patients receiving AXO-Lenti-PD. This time will be varied between patients to aid in the blinding of the patient, their relatives and other personnel involved in the clinical study. All patients will then be transferred to the neurosurgical intensive care unit and monitored according to Section 10.5.8.

The unblinded neurosurgeon, surgical team or pharmacist will have no further involvement in the assessment of the patients in Part B and the maintenance of the blind is further detailed in the Surgical and Pharmacy Manuals.

11 ASSESSMENT OF SAFETY

Assessment of safety within this study will include:

- the assessment of safety to patients from AXO-Lenti-PD IMP and,
- the assessment of safety to patients, users or other persons from any defects, events or effects caused by or related to the AXO-Lenti-PD Administration System and,
- the assessment of safety of the surgical procedure and any other study procedure to patients (not including the AXO-Lenti-PD Administration System)

Additionally, any deficiencies of the AXO-Lenti-PD Administration System will be recorded in cases where those deficiencies could potentially lead to a safety issue.

The assessment of the AXO-Lenti-PD Administration System related events will be in accordance with ISO 14155:2011.

Detailed Safety Reporting Procedures will be provided to all sites.

11.1 Adverse Event Definition

11.1.1 For IMP

An AE is any untoward medical occurrence in a patient that develops or worsens in severity during the conduct of a clinical study of a pharmaceutical product and does not necessarily have a causal relationship to the IMP. This can, therefore, be any unfavourable and unintended physical sign, symptom, or laboratory parameter that develops or worsens in severity during the course of the study, whether or not considered related to the IMP. This includes any AEs resulting from the surgical and study procedures (but not including those directly involving the AXO-Lenti-PD Administration System).

Artefacts of the surgical procedure for administering AXO-Lenti-PD which can be detected on MRI scan, e.g., needle tracts, will not be reported as these are not AEs (unless associated with an AE).

11.1.2 For AXO-Lenti-PD Administration System

An AE is any untoward medical occurrence, unintended disease or injury, or untoward clinical signs (including abnormal laboratory findings) in patients, users or other persons, whether or not related to the AXO-Lenti-PD Administration System.

11.2 Procedures for Recording Adverse Events

Non-directive questioning should be adopted for eliciting AE information at all patient evaluation time points. Examples of such questions include: “How have you felt since your last clinic visit?” or “Have you had any new or changed health problems since you were last here?”.

All AEs must be described in the source documents and appropriate section of the eCRF and their seriousness and putative relationship (causality) to the IMP, AXO-Lenti-PD Administration System and/or protocol procedures (including surgery) noted. Adverse events are recorded following signature of the informed consent; this is to ensure that any AEs associated with the screening procedures are captured. Pre-existing conditions are to be recorded as concurrent medical conditions/history and would only be classed as an AE should there be a worsening or complication of the condition. For pre-existing clinical PD symptoms, only worsening or a complication requiring a change in their PD medication will be recorded as AEs. Changes in C-SSRS scores during the course of the study that indicate worsening should be evaluated by the investigator for clinical significance, and if clinically significant (e.g. alteration in medical care or intervention is required), an associated AE should be recorded, if present. The AE should be the primary underlying clinical manifestation assessed as clinically significant, and not the change in score itself.

Whenever possible, a diagnosis should be recorded on the AE eCRF rather than individual signs and symptoms. Only one AE term should be recorded in the event field on the AE eCRF. Correct medical terminology should be used; colloquialisms and abbreviations should be avoided. Details of AE data collection can be found in the data entry guidelines.

11.3 Abnormal Laboratory Values

A laboratory test result should be assessed as clinically significant or not clinically significant, and should be reported as an AE if it meets any of the following criteria:

- Accompanied by clinical symptoms
- Results in a medical intervention or a change in concomitant therapy
- Clinically significant in the investigator’s judgement

If a clinically significant laboratory abnormality is a sign of a disease or syndrome (e.g., haemoglobin below the lower limit of normal associated with anaemia), only the diagnosis should be recorded. If the abnormality is not a sign of a disease or syndrome, the abnormality itself should be recorded (e.g., low calcium).

11.4 Abnormal Vital Sign Values

A vital sign result should be reported as an AE if it meets any of the following criteria:

- Accompanied by clinical symptoms
- Results in a medical intervention or a change in concomitant therapy
- Clinically significant in the investigator’s judgement

If a clinically significant laboratory abnormality is a sign of a disease or syndrome (e.g. high blood pressure), only the diagnosis should be recorded (i.e., hypertension).

11.5 Relationship of an Adverse Event

The investigator, or medically qualified designee, is required to provide an assessment of relationship of AEs to both the IMP, the AXO-Lenti-PD Administration System and/or surgical or study procedures. Causal relationships will include: not related, unlikely (tabulated as not related), possibly, probably, or definitely related. The sponsor additionally will review all AEs and make an assessment of relationship. Should there be any discrepancy between site and sponsor assessment of AEs the most conservative (cautious) assessment will be taken.

11.5.1 For IMP

An event will be considered “not related” to use of the IMP if any of the following tests are met:

- There is an unreasonable temporal relationship between administration of the IMP and the onset of the event (e.g. the event occurred before administration of the IMP) for it to be considered product-related
- A causal relationship between the IMP and the event is biologically implausible (e.g. death as a passenger in an automobile accident)
- A clearly more likely alternative explanation for the event is present (e.g. typical adverse reaction to a concomitant drug and/or typical disease related event)

An event not meeting any of the above definitions for “not related” will be considered “related” to the IMP.

11.5.2 For AXO-Lenti-PD Administration System

For the AXO-Lenti-PD Administration System, AEs should be assessed as “related” if the investigator considers there is reasonable possibility that the event is due to the AXO-Lenti-PD Administration System itself. Otherwise, the relationship should be assessed as “not related”.

For the relationship to the AXO-Lenti-PD Administration System this includes AEs resulting from insufficient or inadequate instructions for use, deployment, implantation, installation, or operation, or any malfunction of the system. The definition also includes any event resulting from use error or from intentional misuse of the system.

11.5.3 For Surgical or Study Procedures

For the surgical or study procedures not involving the AXO-Lenti-PD Administration System, AEs should be assessed as “related” if the investigator considers there is reasonable possibility that the event is due to the procedure itself. Otherwise, the relationship should be assessed as “not related”.

11.6 Severity of an Adverse Event

For each AE, the severity (intensity), (based on the Common Terminology Criteria for AEs (CTCAE) v4.03), must be recorded as one of the following:

Mild: Asymptomatic or mild symptoms; clinical or diagnostic observations only; intervention not indicated

Moderate: Minimal, local or non-invasive intervention indicated

Severe: Significant impairment of function, hazardous to health (incapacitating)

All severe AEs recorded on the eCRF will be automatically notified to the sponsor for further evaluation by the Medical Monitor and SMC members as appropriate and in accordance with Section 8.4.8.

11.7 Serious Adverse Event Definition

Adverse events are classified as either serious or non-serious. Investigators are required to notify Axovant Sciences' pharmacovigilance service provider if a patient has a serious adverse event (SAE) in accordance with Section 11.9.

11.7.1 For IMP, Surgical or Study Procedures

An SAE is defined according to ICH E6/E2A criteria as any untoward medical occurrence that results in:

- Death
- Life-threatening AE (i.e. the patient was at immediate risk of death from the event as it occurred); does not include an event that, had it occurred in a more severe form, might have caused death
- Requires or prolongs in-patient hospitalisation
- Results in persistent or significant disability/incapacity (refers to a substantial disruption of one's ability to conduct normal life functions)
- Congenital anomaly/birth defect
- Important medical event that may not result in death, be life-threatening, or require hospitalisation, but may jeopardize the patient and may require medical or surgical intervention to prevent one of the outcomes listed in this definition

Investigators and sponsor will assess whether the SAE is related to the IMP, surgical or study procedures (not the AXO-Lenti-PD Administration System) according to these definitions.

11.7.2 For AXO-Lenti-PD Administration System

An SAE is defined according to ISO 14155:2011 as an AE that:

- led to death,
- led to serious deterioration in the health of the patient, that either resulted in
    - a life-threatening illness or injury, or
    - a permanent impairment of a body structure or a body function, or
    - in-patient or prolonged hospitalization, or
    - medical or surgical intervention to prevent life-threatening illness or injury or permanent impairment to a body structure or a body function

11.8 Elective Procedures

For the purpose of this protocol, the following conventions will apply for SAE reporting of elective surgery. A pre-scheduled elective procedure or a routinely scheduled treatment is not to be considered an SAE, even if the patient is hospitalised, provided the site stipulates that:

- The condition requiring the pre-scheduled elective procedure or routinely scheduled treatment was present before and did not worsen or progress between the patient's consent to participate in the clinical trial and the time of the procedure or treatment
- The pre-scheduled elective procedure or routinely scheduled treatment is the sole reason for admission and intervention
- Any untoward medical event occurring during the pre-scheduled elective procedure or routinely scheduled treatment should be recorded as an AE or SAE

Admission for social reasons such as respite care or for monitoring an existing condition is not considered a SAE unless it is concomitant with a worsening of the condition.

11.9 Reporting a Serious Adverse Event

All SAEs that occur during the study (including the protocol-defined follow-up period), regardless of judged relationship to the IMP, AXO-Lenti-PD Administration System or surgical/study procedures, must be immediately reported to the sponsor's pharmacovigilance provider. The report must be made using the eCRF system (or by email or fax on paper form as a back-up) within 24 hours of the investigator, any other agent's acting on behalf of the sponsor, or the sponsor's knowledge of the event (if the SAE is reported directly to the sponsor). The investigator completes the form to the greatest extent possible. As a minimum the study ID, patient ID, SAE term and reporter's ID should be completed. A copy of all relevant source documents including, but not limited to, hard copies of the corresponding completed AE, Medical History, and Concomitant Medications pages of the eCRF should also be

provided if the pharmacovigilance provider is unable to access the information from the eCRF system.

The timely provision of the SAE report to Axovant Sciences' pharmacovigilance provider should not be delayed if the information is partial or incomplete. The same procedure and timelines as indicated above should be used for follow-up information for all SAEs.

Each report of a SAE will be reviewed and evaluated to assess the nature of the event and the relationship of the event to the IMP and to underlying disease in addition to the AXO-Lenti-PD Administration System or surgical/study procedure. This will be done by the investigator and the sponsor (the latter will assess expectedness based on the information in the IB; all SAEs related to the AXO-Lenti-PD Administration System will be classed as unanticipated). Based on this assessment, a decision will be made concerning the need for reporting to the regulatory authorities and other committees as necessary (the most conservative (cautious) approach always being taken should there be any discrepancy in assessment between the investigator and sponsor).

The sponsor's pharmacovigilance provider will report all suspected unexpected serious adverse reactions (SUSARs) to the Regulatory Authorities in accordance with ICH GCP and any local pharmacovigilance regulatory reporting requirements, after either the investigator, any agent acting on behalf of the sponsor, or the sponsor being notified of the SUSAR.

In accordance with MedDev 2.7.3 and Directives 90/385/EEV Annex 7 and 93/42/EEC Annex X respectively, the sponsor's pharmacovigilance provider will also report to the Regulatory Authorities all SAEs (related or not).

If a female patient, or the female partner of a male patient treated with AXO-Lenti-PD becomes pregnant or is found to have a positive pregnancy test within three months of treatment with AXO-Lenti-PD, this matter must be reported immediately by the Investigator via the eCRF reporting form to the pharmacovigilance provider who, in turn, will report the finding to the sponsor. Pregnancy is not considered a SAE but will be reported in the same manner to ensure immediate notification.

11.10 Reporting Device Deficiencies

Any deficiency in the AXO-Lenti-PD Administration System defined as an inadequacy with respect to its identity, quality, durability, reliability, safety or performance which includes malfunctions, use errors, and inadequate labelling will be recorded in the eCRF and reported immediately to the sponsor and the equipment quarantined.

Device deficiencies that did not lead to an AE but could have led to a medical occurrence should also be reported e.g.

a) if suitable action had not been taken,

b) if intervention had not been made, or

c) if circumstances had been less fortunate,

The sponsor will review all device deficiencies and determine and document in writing whether they could have led to a serious adverse device effect (SADE) and report in accordance with regulations to all relevant parties.

11.11 Adverse Events of Special Interest (AESIs)

AESIs include the following:

- Development of any new / recurrent cancer*
- Development of infection (opportunistic and serious infections)
- Immunogenicity related reactions, including antibody responses to the gene expression protein*
- Prolonged and sustained AEs related to IMP e.g. sustained "ON" / "OFF" state phenomena

**Blood samples for qPCR and/or Immunological analysis will be taken to investigate any potential involvement of IMP in the event that a patient enrolled in the study experiences an AE of this nature.*

All AEs recorded on the AE eCRF will be evaluated by the investigator to identify any AESIs. This evaluation will be recorded in the eCRF and AESIs promptly notified to the sponsor and Axovant Sciences' pharmacovigilance provider.

11.12 Biopsies

In the event of a suspicious death relating to a SUSAR, per the regulatory recommendations for ATMPs, a tissue biopsy of the relevant organ may be carried out if feasible. More details about the procedure are provided in the Laboratory Manual. The patient will indicate their consent to this procedure (or not) on the informed consent form.

12 SAFETY MONITORING COMMITTEE (SMC)

An independent SMC will be formed prior to the study initiation to undertake ongoing review of the study safety data including the recommendation to dose escalate, administer AXO-Lenti-PD to further patients within a dose level, to move to Part B, to de-escalate the dose or to suspend/terminate enrolment. The primary role of the SMC is to ensure the protection and safety of patients participating in the study. The SMC will review the general progress of the study and assist in resolving any issues that may arise. Safety data will be provided at regular intervals to the SMC in the form of unblinded summary reports or data listings from an independent statistical centre designated by the sponsor. The composition and responsibilities, together with the agreed schedule for reviewing data, will be detailed in a SMC charter, a guidance document approved and signed by all SMC members prior to the start of the study.

13 STATISTICAL CONSIDERATIONS

13.1 Statistical Analysis Plans

A comprehensive statistical analysis plan (SAP) will be produced to detail the full analyses to be conducted, the SAP will be prepared before the first SMC review meeting. Minor changes to the statistical methods set out in this protocol need not be recorded as a protocol amendment but must be documented in the SAP and in the CSR.

13.2 Data Listings and Summaries

Treatment Groups are defined as follows:

Treatment Group	Part	Treatment
1	A	Dose Level 1
2	A	Dose Level 2
3	A	Dose Level 3
4	B	Optimal dose from Part A
5	B	Imitation Surgical Procedure

All eCRF data and derived study endpoints will be listed. Listings will be sorted by Treatment Group, Patient Number and Time point.

The standard summary statistics for categorical variables are the number and proportion (expressed as percentage) of patients in each category. The standard summary statistics for continuous variables are mean, standard deviation, median, minimum and maximum.

Baseline characteristics will be summarised by Treatment Group. Patient disposition will be summarised by Treatment Group and Time point. Study endpoints, together with, if appropriate, absolute and/or percentage change from baseline will be summarised by Treatment Group and Time point. A pooled summary will also be provided for Treatment Groups 1 to 4 inclusive (all treated patients). An additional summary will be provided pooling Treatment Group 4 and the corresponding Dose Level from Part A.

Figures may be generated as appropriate.

13.2.1 Conduct of Analysis

The statistical analysis will be undertaken by the sponsor. The statistical analysis and report will conform to the relevant ICH requirements.

SAS version 9.2 or later will be used for the analysis.

Summaries will be based on the safety population which will include all patients who received all or part of a dose of study treatment, including ISP.

13.3 Analysis Populations

13.3.1 Safety Analysis Set

The *Safety Analysis Set* comprises all patients who have started general anaesthesia for either active or ISP.

The Safety Analysis Set is the principal analysis set for the analysis of safety data.

13.3.2 Activity Evaluable Analysis Set

The *Activity Evaluable Analysis Set* comprises all patients who successfully received active treatment (Treatment Groups 1 - 4) or who have successfully undergone ISP (Treatment Group 5) and also have a baseline and at least one post-baseline UPDRS score available. This dataset will exclude any patients that are replaced according to Section 8.4.4.

The Activity Evaluable Analysis Set is the principal analysis set for the analysis of signs of activity.

13.4 Missing Data

There will be no imputation for missing data. Summary statistics will be based on all available data.

13.5 Study Conduct

Data concerning the conduct of the study (e.g. allocation to analysis sets, inclusion/exclusion criteria, patient disposition/withdrawals, compliance, protocol violations, study termination/duration) will be listed and summarised descriptively.

13.6 Analysis of Safety

13.6.1 Safety Endpoints

The Safety Endpoints are:

- The incidence, causality and severity of AEs

- Laboratory parameters
- Vital signs
- Brain MRI
- ECG
- Physical examination
- Survival

13.6.2 Safety Summaries

Unless otherwise specified, the standard summary statistics will be used to summarise safety data.

The primary Time point for the assessment of safety data is three months after surgery.

13.6.3 Adverse Events

The number and percentage of patients with treatment emergent AEs will be summarised. The AEs recorded prior to general anaesthesia will be deemed non-treatment emergent, will not be summarised and will be separated from the treatment emergent AEs in the final listings.

An overall summary will include the number and percentage of patients with:

- A fatal AE
- At least one serious AE
- At least one severe AE
- At least one related AE
- At least one AE of special interest
- Without any AE's

Additional tables will show the number and percentage of patients with treatment emergent AEs, fatal treatment emergent AEs, serious treatment emergent AEs, severe treatment emergent AEs, related treatment emergent AEs, severe related treatment emergent AEs, and treatment emergent AEs of special interest broken down by System Organ Class, High Level Term and Preferred Term.

13.6.4 Duration of Survival

Patient deaths, together with survival from day of surgery, will be listed and summarised. If the number of deaths is at least five, then Kaplan-Meier survival probabilities will be presented.

13.7 Analysis of Activity Endpoints

13.7.1 Activity Endpoints

The Activity Endpoints are:

- The change in Unified Parkinson's Disease Rating Scale (UPDRS) scores for each part compared to baseline in defined "OFF" and "ON" medication states
- The change in time during the waking day the patient is in the "OFF" state compared to baseline as assessed by Hauser patient diaries
- The changes from baseline in the patients' total score on activity of daily living using the PD Questionnaire 39 (PDQ-39)
- The changes from baseline in health related quality of life using the EQ-5D-5L
- The change in dyskinesia rating scale from baseline using the Rush Dyskinesia Rating Scale (RDRS) in "OFF" and "ON" states
- The change from baseline in cognition (Montreal Cognitive Assessment (MoCA)
- The change from baseline in depression/mood (HADS)
- The change from baseline in apathy using the Starkstein Apathy Scale
- The change from baseline in impulsivity (QUIP-RS)
- The change in Clinical Global Impression (CGI) Scale from baseline
- The change from baseline in dementia using the Mattis Dementia Rating Scale–2™ (DRS-2)
- The change from baseline in the Hoehn and Yahr Scale in "ON" and "OFF" states
- The change from baseline in the L-DOPA Equivalent Daily Dose (LEDD)
- The change from baseline in L-DOPA dose

13.7.2 Activity Summaries

Unless otherwise specified, the standard summary statistics will be used to summarise activity.

The primary Time point for the assessment of activity data is six months after surgery.

13.8 Other Analyses

13.8.1 Biodistribution and Vector Shedding Analysis

The Biodistribution Endpoint is

- Potential distribution in the blood and urine of AXO-Lenti-PD as assessed by polymerase chain reaction (qPCR)

The Vector Shedding Endpoint is:

- Potential vector shedding in the urine of AXO-Lenti-PD as assessed by reverse transcriptase PCR (RT-PCR)

Unless otherwise specified, the standard summary tables will be used to summarise biodistribution and vector shedding.

13.8.2 Immunology Analysis

The Immunology Endpoint is:

- The incidence of humoral responses to AXO-Lenti-PD administration.

Unless otherwise specified, the standard summary tables will be used to summarise immunology.

13.8.3 Exploratory Endpoints

The Exploratory Endpoints are:

- The percentage change from baseline in the dopamine levels using [^{11}C]-(+)-PHNO PET BP_{ND}
- Correlation between the percentage change from baseline in the dopamine levels using [^{11}C]-(+)-PHNO PET BP_{ND} at "OFF" state and the percentage change from baseline in UPDRS scores at "OFF" state
- The change from baseline in Area Under the Curve (AUC) response of UPDRS Part 3 to L-DOPA challenge
- The change in Non-Motor Symptom Scale (NMSS) from baseline
- The change in Patient Global Impression (PGI) Scale from baseline
- Exploratory research on blood sampling for PD-related research, including but not limited to genomic analysis

13.8.4 Exploratory Analyses

Exploratory Analyses will include but not be limited to summarising the Exploratory Endpoints. Any Exploratory Analyses not specified in the Protocol or the SAP must be clearly described as such in the CSR, as appropriate.

Unless otherwise specified, the standard summary statistics will be used to summarise exploratory endpoints.

13.9 Timing Analysis

There are no formal interim analyses.

Unblinded listings and summaries will be submitted periodically to the SMC. The timing of the SMC submissions will be specified in the SMC Charter.

During Part B, blinded listings and summaries will be generated periodically to allow routine data reporting to regulatory agencies and IRB/IECs. No changes in study conduct are anticipated to result from the generation of these listings.

The CSR will report analyses triggered by all patients either reaching the 6-month activity assessment or withdrawing before 6 months.

During the follow-up period (from the last patient reaching 6 months or withdrawing to the last patient reaching 15 years after surgery or withdrawing) annual summaries of safety, activity and survival will be generated.

13.10 Sample Size

This is an exploratory Phase I/II study and the primary objective is to evaluate safety. No formal sample size calculation has been performed. The sample size is typical of Phase I/II studies of a similar nature.

13.11 Safety Monitoring Committee Data Review

Listings and tables will be provided as appropriate for review by the SMC in accordance with the SMC Charter. The SMC may make recommendations relating to the study including but not limited to recommendations relating to the dosing of future patients (Section 8.1).

The SMC listings and summaries will be a subset of the listings and summaries intended for the CSR.

13.12 Deviations from the Planned Statistical Analysis

All deviations from the planned statistical analysis will be documented in the CSR.

14 QUALITY CONTROL AND QUALITY ASSURANCE

14.1 Data Collection and Source Documents

As part of the responsibilities assumed by participating in the study, the investigator agrees to maintain adequate and accurate medical records for the patients treated under this protocol. Medical records include source documents/data including, but not limited to, the patient's clinic and/or office chart, hospital chart, informed consent forms, treatment notes, laboratory reports, pharmacy records, radiographs, recorded data from automated instruments, and any other records maintained to conduct and evaluate the clinical trial.

Source documents/data (paper or electronic) are those original documents in which patient data are recorded and documented for the first time, and should be attributable (signed and dated). Before study initiation, the source documents/data required for the study must be defined, as well as any data that will be entered directly into the eCRFs (i.e., no prior written or electronic record of the data).

The investigator will ensure that original source documents/data (or a certified copy) are retained at the site at all times.

For patients who provide informed consent and were not entered/randomised into the study, the reason the patient was not entered/randomised, e.g. did not meet one or more inclusion criteria will be documented in the source notes. Any patients who withdraw consent or who are lost to follow-up etc. will also be documented.

14.2 Deviations from Protocol and GCP

The investigator agrees to conduct the study in accordance with the protocol and GCP. Any protocol and/or GCP deviations will be documented in accordance with Section 9.1. The deviations will be reviewed by the monitor and the investigator will assist the monitor in ensuring that corrective and preventative action (e.g. re-training of site staff) is undertaken. Major protocol deviations, defined as potentially impacting on patient safety or study integrity, will be reported to the sponsor for review/follow-up and to the IRB/IEC per applicable local requirements. The sponsor will not grant protocol waivers for ineligible patients.

14.3 Study Monitoring and Access to Source Documents

The primary source document for this study will be the patients' medical records. If separate research records are maintained by the investigator, both the medical record and the research records will be monitored/audited for the purposes of the study.

The investigator and institutions involved in the study will permit study-related monitoring and provide direct access to all study records and facilities. Adequate time and space for monitoring visits should be made by the investigator and/or other investigator site staff.

The monitor will visit study facilities at periodic intervals, in addition to maintaining necessary contact through telephone, e-mail, and letter. The monitor will assess, but not limited to, patient enrolment and informed consent procedures; IMP storage, dispensing, administration and accountability; compliance with protocol procedures; completeness and accuracy of data entered onto the eCRFs against original source documents; and the occurrence and reporting of AEs/SAEs. All aspects of the study will be carefully monitored for compliance with the protocol, applicable regulatory requirements, GCP, and the site's standard operating procedures.

The monitor will discuss the conduct and progress of the study with the investigator and other site staff. The investigator must cooperate with the monitor to ensure that corrective action is taken to resolve any problems noted in the course of the monitoring, and that the preventative measures are put into place to prevent recurrence of issues.

The unblinded monitor will have full access to all information whereas the blinded monitor will not have access to any blinded notes or eCRF elements restricted for blinding purposes.

14.4 Audit and Inspection of the Study

During the conduct of the study, the sponsor or its representative may conduct audits of any data and any facility participating in the study. The investigator and institutions involved in the study will permit such study-related audits and provide direct access to all study records and facilities. The investigator must maintain a comprehensive and centralised filing system of all study-related documentation that is suitable for audit by the sponsor or its designated monitors and auditors. The investigator agrees to participate in audits conducted at a convenient time in a reasonable manner.

Government regulatory authorities may also perform inspections either during or after the study. In the event of an inspection by any regulatory authority, the investigator should promptly notify the sponsor. The investigator agrees to cooperate fully with inspections conducted by regulatory authorities and to allow representatives of the regulatory authority access to all study records.

15 REGULATORY AND ETHICAL CONSIDERATIONS

15.1 Good Clinical Practice

The study will be conducted in accordance with ethical principles that have their origin in the Declaration of Helsinki and are consistent with ICH-GCP, any applicable laws and requirements, and any conditions required by a regulatory authority and/or IRB/IEC that approves this study to be conducted in its territory. The assessment of the AXO-Lenti-PD Administration System related events will be in accordance with ISO 14155:2011 and relevant sections of MedDev 2.7.3 and Directives 90/385/EEV Annex 7 and 93/42/EEC Annex X.

15.2 Investigator Responsibilities

The investigator is responsible for the conduct of the trial at his/her site and should ensure that all persons assisting with the trial are adequately qualified, trained and informed about the protocol, any amendments to the protocol, the study treatments, and their trial related duties and functions.

The investigator must maintain a list of sub-investigators and other appropriately qualified persons to whom he/she has delegated significant trial-related duties.

15.3 Patient Information and Consent

Before enrolment into the clinical study, written, personally signed/dated informed consent must be obtained from the patient/legal representative after the nature, scope, and possible consequences of the clinical trial have been explained. The consent form must also be personally counter-signed/dated by the investigator or delegate that conducted the informed consent. The patient’s medical records shall document the informed consent process and that written informed consent was obtained prior to participation in the study.

The investigator will not undertake any measures specifically required for the clinical trial until valid consent has been obtained.

The patient/legal representative will receive a copy of the full patient information and the signed consent form, and the original will be maintained at the site. Any site-specific variation on filing of original/copies of signed informed consent forms will be documented at site. All patients participating in a trial involving an ATMP will receive from the investigator a patient alert card, which identifies the trial /investigator and which has been previously been agreed by the sponsor and approved by the IRB/IEC.

The patient information and consent forms should be revised whenever there are changes to study procedures or when new information becomes available that may affect the willingness of the patient to participate. Patients must be re-consented to the most current version of the information and consent forms as soon as is practical during their participation in the study.

If the patient has a primary physician, and if the patient agrees to them being informed, the investigator should inform the patient's primary physician about their participation in the trial.

15.4 Confidentiality

Patient names or other personal identifiable information will not be supplied to the sponsor or representatives of the sponsor. A patient number will be assigned, recorded in the eCRF, and used when referring to patient data/information. If the patient name appears on any other document (e.g. laboratory reports), it must be removed prior to providing the document to the sponsor. Study data/information stored on a computer will be stored in accordance with local data protection and privacy laws and ensure that patient confidentiality is maintained.

Patients will be informed as part of the informed consent process that representatives of the sponsor, IRB/IEC, or regulatory authorities may inspect their medical records to verify the trial information collected, and that all personal information made available for review will be handled in strictest confidence and in accordance with local data protection and privacy laws.

The investigator will maintain a patient identification list (patient numbers with the corresponding patient names and other relevant identifiers) to allow records to be identified. This list will not be filed in the sponsor files. The patient's identity and personal data will be safeguarded according to national data protection and privacy requirements.

15.5 Protocol Amendments

Neither the investigator nor the sponsor will make changes to this clinical study protocol without obtaining prior written agreement, unless urgent safety measures are necessary to avoid hazard to a patient. In this case the necessary measures will be taken prior to the usual approvals (see Section 15.6).

If a protocol amendment is required, this will be enacted through a formal documented protocol amendment procedure and any substantial amendments must receive approval from all the authorities who approved the original protocol, before being implemented. The patient information and consent forms must also be reviewed and amended if necessary, and must also receive approval from all the authorities who approved the original information and consent forms, before being implemented.

15.6 Urgent Safety Measures

The regulations governing clinical trials state that the sponsor and Investigator are required to take appropriate urgent safety measures to protect patients against any immediate hazards that may affect the safety of patients, and that the appropriate regulatory bodies should be notified according to their respective regulations. According to the European Union (EU) Clinical Trial Directive 2001/20/EC, "…in the light of the circumstances, notably the occurrence of any new event relating to the conduct of the trial or the development of the investigational medicinal product where that new event is likely to affect the safety of the patients, the sponsor

and the Investigator shall take appropriate urgent safety measures to protect the patients against any immediate hazard. The sponsor shall forthwith inform the competent authorities of those new events and the measures taken and shall ensure that the IRB/IEC/REB is notified at the same time." Investigators are therefore required to report any events that may require the implementation of urgent safety measures to the sponsor and the sponsor's pharmacovigilance provider.

15.7 Trial Approval

Before the first patient is enrolled in the study, all ethical and legal requirements must be met. This includes obtaining written approval from the IRB/IEC and the regulatory authority. The sponsor will be responsible for obtaining the appropriate approvals from the regulatory authorities and will provide the investigator with the necessary approval documentation.

The IRB/IEC will be provided, by or on behalf of the investigator, with copies of the protocol, amendments, patient information and consent forms for review. Other documents and supportive information will be provided in accordance with IRB/IEC requirements and relevant legislation. The submission/approval documentation should document what was submitted and approved (including version numbers and dates).

The IRB/IEC and regulatory authorities will be informed of all subsequent amendments, periodic updates, relevant new information and other changes, in accordance with local legal requirements. Protocol amendments will not be implemented until the necessary submissions have been made, and approvals obtained in line with national requirements.

The investigator must keep a record of all communication with the IRB/IEC. This also applies to any communication between the investigator and the authorities.

The investigator will provide copies of all IRB/IEC approvals, re-approvals and other relevant communication/documentation to the sponsor and/or its CRO representatives. This includes:

- A written statement from the IRB/IEC indicating that it complies with relevant legislation, including ICH-GCP. (If this is included in the IRB/IEC approval letter, there is no need for a separate written statement to be obtained).
- The IRB/IEC membership list at the meeting where the clinical trial was reviewed. If the IRB/EC does not allow access to the membership list it must be confirmed in writing by the IRB/IEC that they were quorate and appropriately constituted, per ICH-GCP.
- The Investigator must ensure any requests from the IRB/IEC for additional information /safety reports are communicated to the sponsor and fulfilled.

In addition, unless confirmed in writing by the IRB/IEC or local law as not required, the investigator must also submit to the IRB/IEC:

- Expedited safety reports received (i.e. 7 and 15day / SUSAR reports), as soon as possible

- Periodic reports on the progress of the study, as required, during the conduct of the study
- Any other significant information arising during the study that is deemed by the sponsor/Investigator as relevant to the IRB/IEC

Other trial approvals (R&D committees, radiation committees) will also be sought as necessary.

15.8 Trial Closure

The study site will be closed once the final patient for that site has completed their final visit for the study, all patient data for that site has been reviewed, all data queries addressed and the database locked. After site closure the site will be required to submit the CSR to the IRB/IEC.

Completion or premature termination of the study will be reported by the sponsor to the regulatory agency and by the sponsor or by the investigator to the IRB/IEC as required by local regulation or by the IRB/IEC.

Study materials must be returned, disposed of or retained as directed by the sponsor.

Study documents (including patient records, copies of data submitted to the sponsor and pharmacy records) must be kept secured in accordance with the specific data retention periods that are described in the clinical study site agreement and based upon local requirements for a period not less than 30 years. Study documents must not be destroyed without prior written approval of the sponsor.

15.9 Financial Disclosure

The clinical trial is fully funded by Axovant Sciences and details of this are in the clinical site agreements.

Before the start of the study, the investigator will disclose to the sponsor any proprietary or financial interests he or she might hold in the IMPs or the sponsor company as outlined in the financial disclosure form provided by the sponsor. The investigator agrees to update this information in case of significant changes during the study or within one year of its completion. The investigator also agrees that, where required by law or regulation, the sponsor may submit this financial information to domestic or foreign regulatory authorities in applications for marketing authorisations.

Similar information will be provided by each sub-investigator to whom the investigator delegates significant study-related responsibilities.

16 INSURANCE INDEMNITY

Axovant Sciences has a specialist insurance policy in place which would operate in the event of any patient suffering harm as a result of their involvement in the clinical trial. Evidence in the form of a certificate of insurance will be provided to each participating site.

17 PUBLICATION POLICY

Publication by the site of any data from this study must be carried out in accordance with the clinical study site agreements. A description of this clinical trial will be available on the relevant clinical trial registries.

Appendix 1 World Medical Association Declaration of Helsinki

Ethical Principles for Medical Research Involving Human Subjects

The most recent version of the declaration can be accessed via the link below:

http://www.wma.net/en/30publications/10policies/b3/

Appendix 2 Positron Emission Tomography Scans

Positron Emission Tomography is a highly specialised imaging technique using short lived radiolabelled compounds to produce powerful images of the body's biological function. The compound used in this procedure will be (+)-PHNO labelled with a radioactive isotope. The radiolabelled compound is injected systemically, into the patient. In this study, [^{11}C]-(+)-PHNO will be used to provide information on the dopaminergic function in the striatum of the patients pre-operatively and at the endpoint of the study (six months post AXO-Lenti-PD administration), with an optional scan at 12 months post AXO-Lenti-PD administration. All the PET studies will be performed in an "OFF" state, i.e., all anti-parkinsonian medications stopped for at least 12 hours before scanning. Additional optional "ON" state scans will also be performed at baseline, 6 and 12 months.

[^{11}C]-(+)-PHNO Study

The analysis of changes in dopamine D_2/D_3 receptors' (D_2/D_3R) availability to the PET ligand ['^{11}C]-(+)-PHNO can be used to indirectly measure extracellular dopamine changes in the living human brain. When endogenous dopamine binds to D_2/D_3R, these receptors are unavailable for the binding of the agonist [^{11}C]-(+)-PHNO, and this phenomenon allows dopamine levels to be estimated indirectly from changes in ^{11}C]-(+)-PHNO BP_{ND}. Importantly, as an agonist D_2/D_3R PET ligand, [^{11}C]-(+)-PHNO is more sensitive to changes in dopamine levels than antagonist PET ligands such as [^{11}C]-Raclopride, because dopamine being an agonist binds preferentially to the G-protein coupled ("high affinity") state of the D_2/D_3R, as does the agonist (+)-PHNO, while the antagonist raclopride binds equally to both the G-protein coupled ("high affinity") and the G-protein uncoupled ("low affinity") receptors.[52, 53, 54] Conventionally, specific D_2/D_3R binding of [^{11}C]-(+)-PHNO BP_{ND} is computed from regional brain time activity curves (TAC) using a simplified tissue reference model and the cerebellum as a reference region (as it has very low density of D_2/D_3R, so its TAC reflects non-specific binding levels in other brain regions). If synaptic dopamine concentrations increase, there is a reduction in BP_{ND} that is proportional to the changes in availability of D_2/D_3R due to occupancy by endogenous dopamine.

In this study, an elevation of dopamine synaptic concentrations should be achieved after AXO-Lenti-PD administration compared to pre-operative levels. Accordingly, this change in extracellular dopamine concentrations should be reflected by a proportional reduction of the BP_{ND} of [^{11}C]-(+)-PHNO after surgery when compared to pre-operative levels. A reasonable

goal of the present study would be to increase by ~50% the amount of dopamine available in the synaptic cleft in the putamen of the patients treated with AXO-Lenti-PD. This would induce a significant reduction (normalisation) of the post-operative BP values in the putamen compared to the pre-operative values. In addition, a dose effect could potentially be observed, which would be evidenced by a significant negative correlation between the amplitude of the difference between the putamenal BP values (pre-operative - post operative) and the dose of vector administered.

Additional optional PET scans will be performed in the "ON" medication state at baseline, 6 months and 12 months. The difference between the baseline "OFF" and "ON" medication state scans will indicate the level of change of $[^{11}C]$-(+)-PHNO BP_{ND} following a dose of L-DOPA oral administration, i.e., how much L-DOPA can be converted to dopamine and the consequent reduction this may have in D_2/D_3R availability. This level of change will be compared to the "OFF" medication state scans at baseline and 6 months and optionally 12 months. This will provide a measure of the change in dopamine synthesis and release mediated by AXO-Lenti-PD compared to a dose of L-DOPA.

In addition, comparison of the change in $[^{11}C]$-(+)-PHNO BP_{ND} in the "OFF" and "ON" medication states at baseline with that longitudinally at 6 months and optionally at 12 months will provide an insight into the biological effect of AADC activity from the AXO-Lenti-PD transduced neurons when converting exogenous L-DOPA to dopamine before and following AXO-Lenti-PD striatal administration.

All details regarding PET data acquisition and analysis will be provided in a separate Imaging Manual, which will include details of image acquisition and image reconstruction, and Imaging Report, which will include details of anatomical and kinetic analysis of the PET emission data.

18 REFERENCES

1 Information on Parkinson's disease. Available at European Parkinson's Disease Association website: http://www.epda.eu.com/en/pd-info/

2 American Academy of Neurology Guidelines Summary for patients and their families: medical and surgical treatment for motor fluctuations and dyskinesias in Parkinson's disease, 2006

3 Medtronic DBS Therapy for Parkinson's Disease and Essential Tremor Clinical Summary, 2015

4 Ponce, F and Lozano, AM. Deep brain stimulation state of the art and novel stimulation targets Prog. Brain Res. (2010); 184:311-24

5 NICE CG35, 2006

6 Azzouz, M., Martin-Rendon, E., Barber, R.D., et al. Multicistronic lentiviral vector-mediated striatal gene transfer of aromatic L-amino acid decarboxylase, tyrosine hydroxylase, and GTP cyclohydrolase I induces sustained transgene expression, dopamine production, and functional improvement in a rat model of Parkinson's disease. J. Neurosci. (2002);22:10302-10312

7 Muramatsu, S., Fujimoto, K., Ikeguchi, K., et al. Behavioral recovery in a primate model of Parkinson's disease by triple transduction of striatal cells with adeno-associated viral vectors expressing dopamine-synthesizing enzymes. Hum. Gene Ther. (2002);13:345-354.

8 Bankiewicz, K.S., Eberling, J.L., Kohutnicka, M, et al. Convection-enhanced delivery of AAV vector in parkinsonian monkeys; in vivo detection of gene expression and restoration of dopaminergic function using pro-drug approach. Exp. Neurol. (2002);164:2-14.

9 Bensadoun, J.C., Pereira de Almeida, L., et al. Comparative study of GDNF delivery systems for the CNS: polymer rods, encapsulated cells, and lentiviral vectors. J. Control Release. (2003);87:107-115.

10 Jarraya B, Boulet S, Ralph S, et al Dopamine Gene Therapy for Parkinson's Disease in a Nonhuman Primate Without Associated Dyskinesia Sci Transl Med (2009);1, 2ra4

11 Saenz, D.T. and Poeschla, E.M. FIV: from lentivirus to lentivector. J. Gene Med.(2004);6:S95-104

12 Azzouz, M., Martin-Rendon, E., Barber, R.D., et al. Multicistronic lentiviral vector-mediated striatal gene transfer of aromatic L-amino acid decarboxylase, tyrosine hydroxylase, and GTP cyclohydrolase I induces sustained transgene expression, dopamine production, and functional improvement in a rat model of Parkinson's disease. J. Neurosci. (2002);22:10302-10312

13 Palfi S, Gurruchaga JM, Ralph GS et al. Long-term safety and tolerability of ProSavin, a lentiviral vector-based gene therapy for Parkinson's disease: a dose escalation,

open-label, phase 1/2 trial. Lancet 2014; 383 (9923): 1138-46 (published online 10 Jan 2014)

14 Hannah J. Stewart,, G. Scott Ralph, Liang Fong-Wong, Iain Strickland, Laura McCloskey, Lucy Barnes, Ian Blount, Owen Wells, Christelle J.M. Truran, Alan J. Kingsman, Stéphane Palfi, and Kyriacos A. Mitrophanous , Optimizing Transgene Configuration and Protein Fusions to Maximize Dopamine Production for the Gene Therapy of Parkinson's Disease Human Gene Therapy Clinical Development. September 2016, 27(3): 100-110

15 Grez M, Reichenbach J, Schwäble J, et al. Gene therapy of chronic granulomatas disease: The engraftment dilemma. Mol. Ther., (2011);19, PP: 28-35.

16 Boztug, K, Schmidt, M, Schwarzer, A, Banerjee, PP, et al. Stem-Cell Gene Therapy for the Wiskott–Aldrich Syndrome. NEJM, (2010);363, PP: 1918-1927.

17 Hacein-Bey-Abina, S, Hauer J., Lim, A, Picard C, et al. Efficacy of Gene Therapy for X-Linked Severe Combined Immunodeficiency. NEJM, (2010);363, PP: 355-364.

18 Howe S J, Mansour MR, Schwarzwaelder K, et. al., Insertional mutagenesis combined with acquired somatic mutations causes leukemogenesis following gene therapy of SCID-X1 patients J. Clin. Invest., (2008);118, PP: 3143-3150.

19 Lang, A.E. and Lozano, A.M. Parkinson’s disease. First of two parts. New Eng. J. Med. 339:1044-1053, (1998)

20 Piggott, M.A., Marshall, E.F., Thomas, N., Lloyd, S., Court, J.A., Jaros, E., Burn, D., Johnson, M., Perry, R.H., McKeith, I.G., Ballard, C. and Perry, E.K. Striatal dopaminergic markers in dementia with Lewy bodies, Alzheimer’s and Parkinson’s diseases: rostrocaudal distribution. Brain. 122:1449-1468, (1999)

21 Gerlach, M., Gsell, W., Kornhuhber, J., Jellinger, K., Frieger, V., Pantucek, F., Vock, R. and Riederer, P. A post mortem study on neurochemical markers of dopaminergic, GABAergic and glutamatergic neurons in basal ganglia-thalamocortical circuits in Parkinson syndrome. Brain Res. 741:142-152, (1996)

22 Nutt J.G. Continuous dopaminergic stimulation; is it the answer to motor complications of levodopa? Mov. Disord. 22(1):1-9, (2007)

23 Miller F.G. Sham surgery: An ethical analysis. Science Engineering Ethics 10 (1) :157-166, (2004)

24 Shetty N., Friedman J.H., Keiburtz K., Marshall F.J. and Oakes D. The placebo response in Parkinson’s disease. Parkinson Study Group. Clin. Neuropharmacol. 22(4):207-212, (1999)

25 Goetz C.G., Leurgans S., Raman R. and Stebbins G.T. Objective changes in motor function during placebo treatment in PD. Neurology. 54:710-714, (2000)

26 De la Fuente-Fernandez R., Ruth T.J., Sossi V., Schulzer M., Calne D.B. and Stoessl J. Expectation and dopamine release : mechanism of the placebo effect in Parkinson’s disease. Science. 293:1164-1166, (2001)

27 Freeman T.B., Vawter D.E., Leaverton P.E., Godbold J.H., Hauser R.A., Goetz C.G. and Olanow C.W. Use of placebo surgery in controlled trials of a cellular-based therapy for Parkinson's disease. New Eng. J. Med. 341:988-992, (1999)

28 Freed C.R., Greene P.E., Breeze R.E., Tsai W.Y., DuMouchel W., Kao R., Dillon R.N., Winfield H., Culver S., Trojanowki J.Q., Eidelberg D. and Fahn S. Transplantation of embryonic dopamine neurons for severe Parkinson's disease. New. Engl. J. Med. 344:710-719, (2001)

29 Olanow CW, Goetz CG, Kordower JH, Stoessl AJ, Sossi V, Brin MF, Shannon KM, Nauert GM, Perl DP, Godbold J, Freeman TB.A double-blind controlled trial of bilateral fetal nigral transplantation in Parkinson's disease. Ann. Neurol. 54(3):403-414, (2003).

30 Marks, W.J., Bartus, R.T., Siffert, J., Davis, C.S., Lozano, A., Boulis, N. et al. Gene delivery of AAV2-neurturin for Parkinson's disease: a double-blind, randomised, controlled trial. The Lancet Neurology, 9(12):1164 – 1172, (2010)

31 LeWitt, P.A., Rezai, A.R., Leehey, M.A., Ojemann, S.G., Flaherty, A.W., Eskandar, E.N et al. AAV2-GAD gene therapy for advanced Parkinson's disease: a double-blind, sham-surgery controlled, randomised trial. The Lancet Neurology.10(4): 309 – 319, (2011)

32 Freed C.R., Greene P.E., Breeze R.E., Tsai W.Y., DuMouchel W., Kao R., Dillon R.N., Winfield H., Culver S., Trojanowki J.Q., Eidelberg D. and Fahn S. Transplantation of embryonic dopamine neurons for severe Parkinson's disease. New. Engl. J. Med. 344:710-719, (2001)

33 McRae C., Cherin E., Yamazaki G., Diem G., Vo A.H., Russell D., Ellgring J.H., Fahn S., Greene P., Dillon S., Winfield H., Bjugstad K.B. and Freed C.R. Effects of perceived treatment on quality of life and medical outcomes in a double-blind placebo surgery trial. Arch. Gen. Psychiatry. 61:412-420, (2004)

34 Christine CW, Bankiewicz KS, Van Laar AD, Richardson RM, Ravina B, Kells AP, Boot B, Martin AJ, Nutt J, Thompson ME, Larson PS. Magnetic resonance imaging-guided phase 1 trial of putaminal AADC gene therapy for Parkinson's disease. Ann Neurol. 85(5):704-714, (2019).

35 Yin D, Valles FE, Fiandaca MS, Forsayeth J, Larson P, Starr P, Bankiewicz KS. Striatal volume differences between non-human and human primates. J Neurosci Methods. 2009 Jan 30;176(2):200-5.

36 Committee For Medicinal Products for Human Use (CHMP). Guideline on Follow-Up of Patients Administered with Gene Therapy Medicinal Products. European Medicines Agency, (2009)

37 Hauser, R.A, Friedlander J, Zesiewicz T.A., Adler C.H., Seeberger L.C., O'Brien C.F., Molho E.S., Factor S.A. A home diary to assess functional status in patients with Parkinson's disease with motor fluctuations and dyskinesia. Clin Neuropharmacol. 23(2):75-81, (2000)

38 Fahn, S., Elton, R.L., Members of the UPDRS Development Committee: Unified Parkinson's Disease Rating Scale. In: Fahn S, Marsden CD, Calne DB, Lieberman A, eds: Recent developments in Parkinson's disease. Florham Park, NJ: Macmillan Health Care Information;153–163, (1987)

39 Goetz, C.G., Stebbins, G.T., Shale, H.M., Lang, A.E., Chernick, D.A., Chmura, T.A., Ahlskog, J.E. and Dorflinger, E.E. Utility of an objective dyskinesia rating scale for Parkinson's disease: Inter- and intrarater reliability assessment. Mov. Disord. 9:390-394, (1994)

40 Jenkinson, C., Fitzpatrick, R., and Peto, V. The Parkinson's disease questionnaire. User manual for the PDQ-39, PDQ-8 and PDQ summary index. Health Services Research Unit, Department of Public Health. University of Oxford (1998)

41 Herdman, M., Gudex, C., Lloyd, A., Janssen, M.F., Kind, P., Parkin, D., Bonsel, G., Badia, X. Development and preliminary testing of the new five-level version of EQ-5D (EQ-5D-5L). Quality of Life Research. 20(10): 1727–1736, (2011)

42 Zigmond, A.S., Snaith, R.P. The hospital anxiety and depression scale. Acta Psychiatr Scand. 67(6):361-70, (1983)

43 Nasreddine, Z.S., Phillips, N.A., Bédirian, V., Charbonneau, S., Whitehead, V., Collin, I., Cummings, J.L.C., Chertkow, H. The Montreal Cognitive Assessment, MoCA: A Brief Screening Tool For Mild Cognitive Impairment. J Am Geriatr Soc 53:695–699, (2005).

44 Weintraub, D., Stewart, S., Shea, J.A., Lyons, K.E., Pahwa, R., Driver-Dunckley, E.D., Adler, C.H., Potenza, M.N., Miyasaki, J., Siderowf, A.D., Duda, J.E., Hurtig, H.I., Colcher, A., Horn, S.S., Stern, M.B. and Voon, V. Validation of the Questionnaire for Impulsive-Compulsive Disorders in Parkinson's Disease (QUIP). Mov Disord. 24(10):1461–1467, (2009)

45 Strauss, E., Sherman, E. M. S., Spreen, O. (2006). A Compendium of neuropsychological tests: Administration, norms, and commentary. (3rd. ed.). New York, NY. Oxford University Press (2006)

46 Chaudhuri, K., Martinez-Martin, P., Brown, R., Sethi, K., Stocchi, F., Odin, P., Ondo, W., Abe, K., MacPhee, G., MacMahon, D., Barone, P., Rabey, M., Forbes, A., Breen, K., Tluk, S., Naidu, Y., Olanow, W., Williams, A., Thomas, S., Rye, D., Tsuboi, Y., Hand, A., Schapira, A. (2007). The metric properties of a novel non-motor symptoms scale for Parkinson's disease: Results from an international pilot study Movement Disorders 22(13), 1901-1911. https://dx.doi.org/10.1002/mds.21596

47 Starkstein, S.E., Mayberg, H.S., Preziosi, T. J., Leiguarda, R., and Robinson, R.G. Reliability, Validity and Clinical Correlates of Apathy in Parkinson's disease. J Neuropsychiatry 4(2):134-139, (1992)

48 Hoehn, M.M., Yahr, M.D. Parkinsonism: Onset, progression and mortality. Neurology. 17: 427-442, (1967)

49 Guy W: ECDEU Assessment Manual for Psychopharmacology —Revised (DHEW Publ No ADM 76–338). Rockville, MD, U.S. Department of Health, Education, and Welfare,

Public Health Service, Alcohol, Drug Abuse, and Mental Health Administration, NIMH Psychopharmacology Research Branch, Division of Extramural Research Programs, 1976, pp 218–222

50 Guy W: ECDEU Assessment Manual for Psychopharmacology —Revised (DHEW Publ No ADM 76–338). Rockville, MD, U.S. Department of Health, Education, and Welfare, Public Health Service, Alcohol, Drug Abuse, and Mental Health Administration, NIMH Psychopharmacology Research Branch, Division of Extramural Research Programs, 1976, pp 218–222

51 European Commission. Detailed Guidelines on Good Clinical Practise Specific to Advanced Therapy Medicinal Products. (2009)

52 Shotbolt P, Tziortzi AC, Searle GE, Colasanti A, van der Aart J, Abanades S, Plisson C, Miller SR, Huiban M, Beaver JD, Gunn RN, Laruelle M, Rabiner EA. Within-subject comparison of [(11)C]-(+)-PHNO and [(11)C]raclopride sensitivity to acute amphetamine challenge in healthy humans. J Cereb Blood Flow Metab. 2012 Jan;32(1):127-36

53 Caravaggio F, Nakajima S, Borlido C, Remington G, Gerretsen P, Wilson A, Houle S, Menon M, Mamo D, Graff-Guerrero A. Estimating endogenous dopamine levels at D2 and D3 receptors in humans using the agonist radiotracer [(11)C]-(+)-PHNO. Neuropsychopharmacology. 2014 Nov;39(12):2769-76

54 Payer DE, Guttman M, Kish SJ, Tong J, Adams JR, Rusjan P, Houle S, Furukawa Y, Wilson AA, Boileau I. D3 dopamine receptor-preferring [11C]PHNO PET imaging in Parkinson patients with dyskinesia. Neurology. 2016 Jan 19;86(3):224-30
